# Computer-Aided Studies for Novel Arylhydantoin 1,3,5-Triazine Derivatives as 5-HT_6_ Serotonin Receptor Ligands with Antidepressive-Like, Anxiolytic and Antiobesity Action In Vivo

**DOI:** 10.3390/molecules23102529

**Published:** 2018-10-03

**Authors:** Rafał Kurczab, Wesam Ali, Dorota Łażewska, Magdalena Kotańska, Magdalena Jastrzębska-Więsek, Grzegorz Satała, Małgorzata Więcek, Annamaria Lubelska, Gniewomir Latacz, Anna Partyka, Małgorzata Starek, Monika Dąbrowska, Anna Wesołowska, Claus Jacob, Katarzyna Kieć-Kononowicz, Jadwiga Handzlik

**Affiliations:** 1 Department of Medicinal Chemistry, Institute of Pharmacology, Polish Academy of Sciences, Smętna 12, PL 31-343 Cracow, Poland; kurczab@if-pan.krakow.pl (R.K.); satala@if-pan.krakow.pl (G.S.); 2 Department of Technology and Biotechnology of Drugs, Jagiellonian University, Medical College, Medyczna 9, PL 30-688 Cracow, Poland; s8wealii@stud.uni-saarland.de (W.A.); dlazewska@cm-uj.krakow.pl (D.Ł.); mwiecek@cm-uj.krakow.pl (M.W.); annamaria.lubelska@doctoral.uj.edu.pl (A.L.); glatacz@cm-uj.krakow.pl (G.L.); mfkonono@cyf-kr.edu.pl (K.K.-K.); 3 Division of Bioorganic Chemistry, School of Pharmacy, University of Saarland, Campus B2 1, D-66123 Saarbruecken, D-66123 Saarland, Germany; c.jacob@mx.uni-saarland.de; 4 Department of Pharmacodynamics, Jagiellonian University, Medical College, Medyczna 9, PL 30-688 Cracow, Poland; magda.dudek@uj.edu.pl; 5 Department of Clinical Pharmacy, Jagiellonian University, Medical College, Medyczna 9, PL 30-688 Cracow, Poland; mj.wiesek@gmail.com (M.J.-W.); annairena.partyka@uj.edu.pl (A.P.); awesolowska@cm-uj.krakow.pl (A.W.); 6 Department of Inorganic Chemistry, Jagiellonian University, Medical College, Medyczna 9, PL 30-688 Cracow, Poland; m.starek@uj.edu.pl (M.S.); monika.1.dabrowska@uj.edu.pl (M.D.)

**Keywords:** serotonin receptors, 5-HT_6_ ligands, 1,3,5-triazine, hydantoin, docking, obesity, antidepressive, ADMET in vitro

## Abstract

This study focuses on the design, synthesis, biological evaluation, and computer-aided structure-activity relationship (SAR) analysis for a novel group of aromatic triazine-methylpiperazines, with an hydantoin spacer between 1,3,5-traizine and the aromatic fragment. New compounds were synthesized and their affinities for serotonin 5-HT_6_, 5-HT_1A_, 5-HT_2A_, 5-HT_7_, and dopamine D_2_ receptors were evaluated. The induced-fit docking (IFD) procedure was performed to explore the 5-HT_6_ receptor conformation space employing two lead structures. It resulted in a consistent binding mode with the activity data. For the most active compounds found in each modification line, anti-obesity and anti-depressive-like activity in vivo, as well as “druglikeness” in vitro, were examined. Two 2-naphthyl compounds (**18** and **26**) were identified as the most active 5-HT_6_R agents within each lead modification line, respectively. The 5-(2-naphthyl)hydantoin derivative **26**, the most active one in the series (5-HT_6_R: *K*_i_ = 87 nM), displayed also significant selectivity towards competitive G-protein coupled receptors (6–197-fold). Docking studies indicated that the hydantoin ring is stabilized by hydrogen bonding, but due to its different orientation, the hydrogen bonds form with S5.44 and N6.55 or Q6.58 for **18** and **26**, respectively. Compound **26** exerted anxiolytic-like and antidepressant-like activities. Importantly, it demonstrated anti-obesity properties in animals fed palatable feed, and did not show toxic effects in vitro.

## 1. Introduction

The serotonin receptor 5-HT_6_ is one of the recently discovered members of the 5-HTRs family [1]. It is quite unique, since it includes a short third cytoplasmatic loop in parallel, with a long C-terminal tail, and one introne located in the middle of the third cytoplasmatic loop [2,3]. The localization of 5-HT_6_R is limited to the central nervous system, especially as it is located in the brain areas involved in learning and memory processes. Relatively limited, but intensive, research efforts in this area led to several families of potent 5-HT_6_R ligands [4,5], which have been proposed for potential treatment in the cognitive dysfunction associated with Alzheimer’s disease, anxiety or depression [5,6,7]. Over 20 compounds have qualified for clinical trials, most of which in studies following a cure for Alzheimer’s disease [5]. Nonetheless, none has reached the pharmaceutical market yet. Recent lines of evidence also point towards promising properties of some 5-HT_6_R ligands in the battle against obesity [8,9]. Therefore, the search for new ligands for the 5-HT_6_R, in particular, those with an action directed against obesity, seems to be a challenge for current medicinal chemistry.

**Figure 1:**
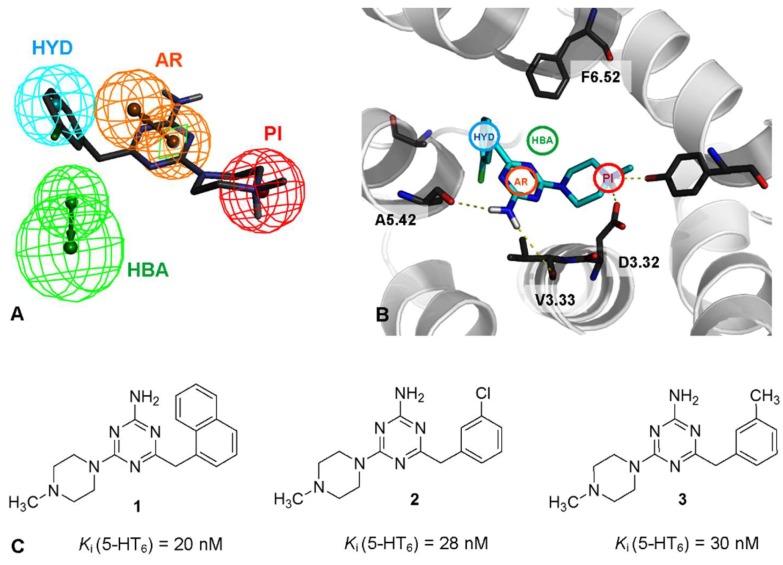
Modified pharmacophore model based of the 1,3,5-triazine with mapped compound **2**, including four key pharmacophore features, namely: A positive ionizable group (PI, red), an aromatic ring (AR, orange), a hydrogen bond acceptor group (HBA, green) and hydrophobic group (HYD, blue) (**A**). The lead compound **2** mapped onto the binding site of 5-HT_6_R (**B**). Examples of the most active 1,3,5-traizne 5-HT_6_ ligands found recently **1**–**3** (**C**).

Lines of evidence have identified several groups of chemical compounds, which displayed significant affinities for the serotonin receptors 5-HT_6_ [4,10,11]. The structure-activity relationship (SAR) analysis performed for the 5-HT_6_ ligands obtained previously enabled to identify pharmacophore features, including a triangle topology with tops of: A bulky hydrophobic area (HYD), positive ionizable nitrogen (PI), and hydrogen bond acceptor (HBA), as well as a central aromatic fragment (AR) (Figure 1) [12].

Taking into consideration the pharmacophore features, our previous studies have successfully found a new group of potent 5-HT_6_R agents among methylpiperazine derivatives of benzyl 2-amino-1,3,5-triazines which fit 3 out of 4 pharmacophore features. Two closed HBAs were the missing feature [13]. The computer-aided SAR analysis allowed us to modify the previous pharmacophore features and it has underlined the notable importance of aromatic substituents separated by a short linker from the 1,3,5-triazine. Thus, a bulky aromatic moiety, represented by the naphthyl (**1**) or *m*-chloro- (**2**) or *m*-methyl- (**3**) substituted phenyl ring (Figure 1) was beneficial for the kind of 5-HT_6_R affinity evaluated experimentally [13].

**Figure 2:**
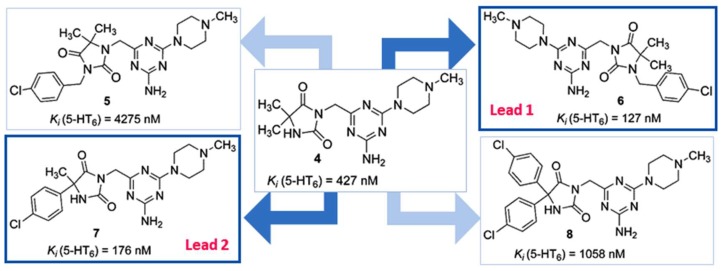
The rationale for the design of novel lead structures of the 5-HT_6_R ligand among hydantoin 1,3,5-triazine derivatives.

Taking into account both, the need to search for the 5-HT_6_R agents with new pharmacological possibilities and the results of previous SAR studies, we decided to explore a novel group of triazine-methylpiperazine derivatives (Figure 2), in which the spacer between 1,3,5-traizine and aromatic fragment is represented by a differently positioned hydantoin system containing both, two closed HBAs and HYD/AR features. In the first step, five pilot compounds (**4**–**8**) were synthesized and evaluated with regard to their affinity for the 5-HT_6_R. These initial studies resulted in two parallel lead structures **5** and **7** (Figure 2). Chemical modifications of the lead structures subsequently provided a series of compounds **9**–**27** (Table 1). Therefore, these studies are focused on the design, synthesis and biological evaluation, including affinity and selectivity for the 5-HT_6_R, as well as computer-aided SAR discussion for an entire series of hydantoin-triazines (**4**–**27**). For representative compounds, anti-obesity and anti-depressive activity in vivo, as well as their “drugability” properties, were also examined.

## 2. Results

### 2.1. Identification of Lead Structures

In the first step, a nonaromatic 5,5-dimethylhydantoin 1,3,5-triazine derivative **4** was found as a moderate 5-HT_6_R ligand after the radioligand binding assay and was therefore selected as an initial lead structure (Figure 2). Then, four different hydantoin moieties were taken into consideration as lead modifications, including: two *N*-substituted *p*-chlorobenzyl derivatives **5** and **6**, with respect to position 3-*N* (**5**) and 1-*N* (**6**), the 5-(4-chlorophenyl)-5-methyl derivative **7** and 5,5-*bis*(4-chlorophenyl)hydantoin **8** (Figure 2).

Compounds **5**–**8** were synthesized and their affinity for the 5-HT_6_R was evaluated in the radioligand binding assays. Results displayed an increase of the 5-HT_6_R affinity in the case of compounds **6** and **7**, whereas both **5** and **8** were much less active than the initial lead **4**. Interestingly, the structure-activity relationship indicated a crucial role for the position of the 1,3,5-triazine substitution at hydantoin ring. Thus, 1,3,5-triazine linked by a methylene group at position 3 of hydantoin seems to be profitable (**6**), whilst that fragment situated at position 1 of hydantoin caused a huge decrease in activity (**5**). Taking into account these results, both 1-(4-chlorobenzyl)-3-((4-amino-6-(4-methylpiperazin-1-yl)-1,3,5-triazin-2-yl)methyl)-5,5-hydantoin (**6**), and the 3-((4-amino-6-(4-methylpiperazin-1-yl)-1,3,5-triazin-2-yl)methyl)-5-(4-chlorophenyl)-5-methylhydantoin (**7**) were selected in parallel as two particularly promising lead structures (Lead 1 and Lead 2, Figure 2). These two leads were subsequently modified within their aromatic area to generate a library of two distinct series of derivatives, i.e., compounds **9**–**18** and compounds **19**–**26**, respectively (Table 1).

### 2.2. Chemical Synthesis of Compounds ***5**–**26***

**Scheme 1:**
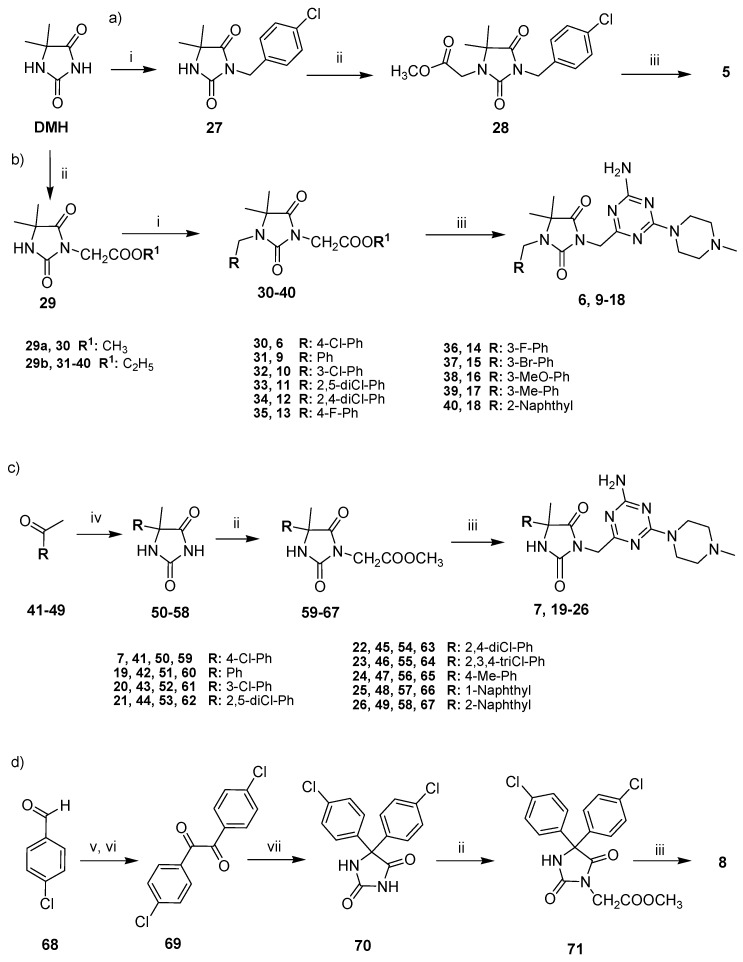
Synthesis of compounds **5**–**26** (Table 1); (**a**) 3-arylmethylhydantoin product **5**; (**b**) 1-arylmethylhydantoin derivatives (**6**, **9**–**18**); (**c**) 5-aryl-5-methylhydantoin derivatives (**7**, **19**–**26**); (**d**) 5,5-diarylhydantoin derivative and triazines (**8**). Reagents and conditions: (i) RCH_2_Cl, K_2_CO_3_, TEBA, acetone, 3–5 h reflux; (ii) BrCH_2_COOMe, K_2_CO_3_, TEBA, acetone, 5–7 h reflux; (iii) 1-(imino(4-methylpiperazin-1-yl)methyl)guanidine hydrochloride, MeONa, reflux for 3–5 h, then rt, 10–20 h; (iv) KCN, (NH_4_)_2_CO_3_, 50% EtOH, 55 °C; (v) KCN, EtOH, 1 h reflux; (vi) HNO_3_ conc., 3 h reflux; (vii) EtONa, urea, 2 h reflux, HCl.

The final compounds **5**–**26** were obtained using four different synthesis pathways (Scheme 1a–d). The synthesis of 5,5-dimethylhydantoin triazine derivative **4** was described earlier [14]. As for the syntheses of the 3-benzyl derivative (**5**) and the 1-arylmethyl ones (**6**, **9**–**18**), the commercial 5,5-dimethylhydantoin (DMH) was a starting material, which was substituted with an appropriate arylmethyl halide and methyl bromoacetate in a different order, respectively (a and b, Scheme 1). The reactions were performed as basic two-phase alkylations in acetone in the presence of a catalytic amount of benzyltriethylammonium chloride (TEBA). In case of **5**, an alkylation with 4-chlorobenzyl at position 3 was the first step (**27**) [15], followed by the introduction of the methyl acetate fragment at position 1 (**28**, Scheme 1a). In contrast, the methyl ester **29a** for the synthesis of **6** and the ethyl ester **29b**, i.e., the common first intermediate for the synthesis of **9**–**18,** were obtained by an alkylation of 5,5-dimethylhydantoin with a suitable alkyl bromoacetate at position 3. The ester **29** was substituted using commercial arylmethyl halides to give compounds **30**–**40**. Although a variety of purification methods was applied, including extraction, crystallization with charcoal and chromatography, only compounds **33**, **34** and **40** gave precipitates (purity > 90%). Compounds **30**–**32** and **35**–**39** were obtained as glue-like residues with varying contents of the target product (44–81%) and were used for final cyclization in the crude form. In the case of 5-aryl-5-methylhydantoin derivatives (**7**, **19**–**26**), standard Bucherer-Bergs cyclic condensation of suitable commercial ketones (**41**–**49**) was the first synthetic step providing 5-aryl-5-methylhydantoins **50**–**58**. Next, esters **59**–**67** were synthesized via *N*-alkylation of **50**–**58** in the hydantoin position 3 with methyl bromoacetate, under similar conditions to those for **29** and purification methods as those for **30**–**40** (Scheme 1c). Among the esters (**59**–**67**), compounds **60** and **66** were obtained in glue forms (purity 76–85%) with the remaining compounds (**59**, **61**–**65** and **67**) yielding white crystals (purity 95–100%). The ester intermediate for compound **8** was obtained within a 3-step synthesis, starting from the acyloin condensation of commercial 4-chlorobenzaldehyde (**68**), followed by oxidation to benzyl compound **69**. The benzyl intermediate was used to perform a cyclic condensation with urea providing 5,5-*bis*(4-chlorophenyl)hydantoin (**70**), followed by an alkylation with methyl bromoacetate in position 3 of the hydantoin, in the aforementioned conditions of two-phase alkylation, to produce ester **71** (Scheme 1d). All ester hydantoin intermediates (**28**, **30**–**40**, **59**–**67**, and **71**) were converted into the 1,3,5-triazine final products (**5**–**26**) via cyclic condensation with 1-(imino(4-methylpiperazin-1-yl)methyl)guanidine, which proceeded in sodium methanolate under the conditions corresponding to those for a series of benzyl-1,3,5-triazines described previously [13,14].

### 2.3. Molecular Modeling

Molecular docking was performed to gain an insight into the binding mode of the library of compounds synthetized (**5**–**26**) to the recently developed 5-HT_6_R homology models [13] built on the β_2_ adrenergic receptor template and optimized for the structures of Lead 1 and 2. The molecular docking indicated that newly synthesized compounds, generally, exhibited a very consistent binding mode with recently reported 5-HT_6_ ligands [16,17,18]. An influence of the topology of aromatic substituents and the position of the 1,3,5-triazine substitution at the hydantoin ring on the binding has been observed (Figure 2) and was in good agreement with results of the radioligand binding assay (see below).

### 2.4. Pharmacology

#### 2.4.1. Radioligand Binding Assay

Compounds **5**–**26** were evaluated with respect to their affinity and selectivity for the target 5-HT_6_R in radioligand binding assays based on the human serotonin receptors 5-HT_6_, 5-HT_1A_, 5-HT_2A_, 5-HT_7b_R and dopaminergic D_2L_R, all of whom were expressed stably in HEK-293 cells. Five selective radioligands were employed (Table 1). Results expressed as *K*_i_ indicate that all derivatives of both leads displayed submicromolar affinities for the target 5-HT_6_R. More active agents (**6**, **7**, **18**, **23**, **25** and **26**) exerted *K*_i_(5-HT_6_R) values lower than 200 nM. Compound **26** was the most active one (*K*_i_ = 87 nM) and also highly selective over 5-HT_1A_R, 5-HT_2A_R, and D_2L_R, with some selectivity over 5-HT_7_R (6-fold). Furthermore, a slightly less active 5-HT_6_R agent, compound **25**, was highly selective with respect to all competitive GPCRs tested (Table 1). Most of the compounds displayed much weaker affinities for 5-HT_1A_, 5-HT_2A_ and D_2L_R receptors than for 5-HT_6_R. Notably, compounds **8**–**11**, **14**, **17** and **24** were more potent for 5-HT_7_R than for the target 5-HT_6_R, in particular, compound **14** (5-HT_7_R; *K*_i_ = 88 nM), which was also selective toward 5-HT_7_R (Table 1).

#### 2.4.2. Behavioral Tests In Vivo

The most potent 5-HT_6_R agent identified in the radioligand binding assays, i.e., compound **26**, was selected for behavioral assays to determine its antidepressant- and anxiolytic-like properties in vivo in male Wistar rats.

##### Antidepressant-Like Activity of Compound **26**

**Figure 3:**
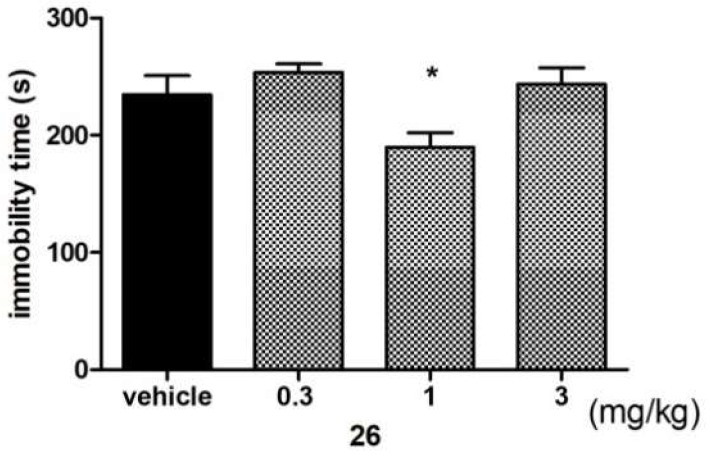
Influence of compound **26** on the immobility time of rats in the forced swim test. Compound **26** was administered i.p. 60 min before the test. The animals were observed for 5 min. The data are presented as the mean ± SEM of 6–8 rats. The data were statistically evaluated by one-way ANOVA followed by Bonferroni’s post-hoc test, * *p* < 0.05 vs. vehicle group.

In the forced swim test (FST), compound **26** significantly decreased immobility time about 20% (ANOVA: F(3,28) = 3.7674, *p* < 0.05) vs. vehicle treated group. A U-shaped dose-response in the FST test for compound **26** was observed (Figure 3).

This effect seems to be typical for some antidepressants with different mechanisms of action also producing U-shaped dose-response curves in some animal models and evaluated for antidepressant-like activity [19,20]. Moreover, this effect was also observed for other 5-HT_6_ ligands (i.e., SB258585) [21].

##### Anxiolytic-Like Activity of Compound **26**

**Figure 4:**
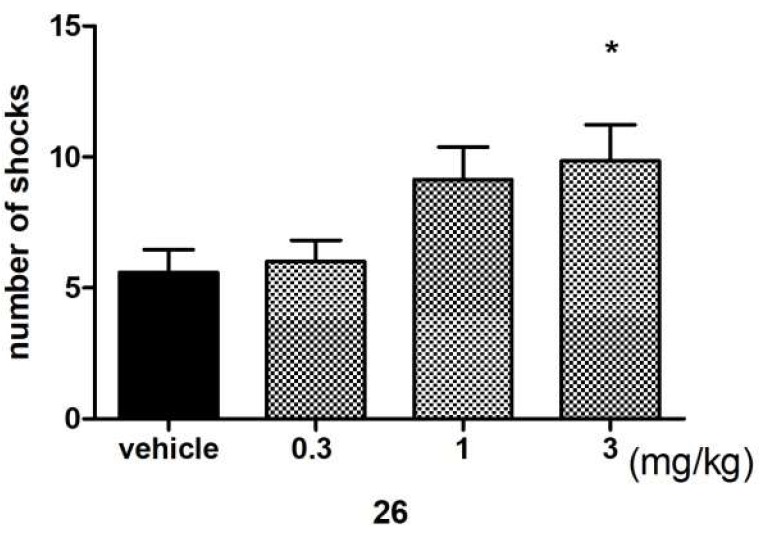
Compound **26** increases the number of shocks accepted in the Vogel conflict test in rats. Compound **26** was administered i.p. 60 min before the test. The animals were monitored for 5 min. The data are presented as the mean ± SEM of 6–8 rats. The data were statistically evaluated by one-way ANOVA followed by Bonferroni’s post-hoc test, * *p* < 0.05 vs. vehicle group.

Anxiolytic-like activity was assessed using a Vogel conflict drinking test. As demonstrated in Figure 4, compound **26**, administered at a dose of just 3 mg/kg, has significantly increased (by 76%) the number of accepted shocks (ANOVA: F(3,22) = 3.4967, *p* < 0.05) during the 5 min experimental session. Thus, compound **26** (at a dose of 3 mg/kg) exerted anxiolytic-like activity in this behavioral test.

#### 2.4.3. Metabolic Assays In Vivo

**Figure 5:**
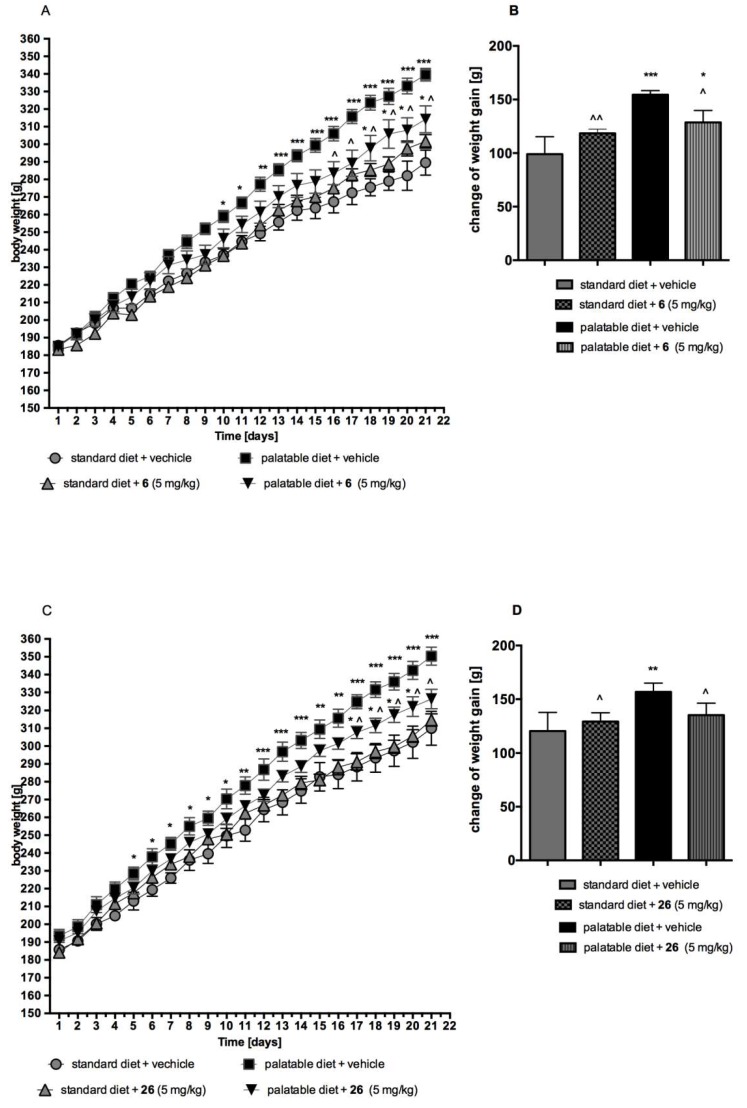
Influence of long-term administration of **6** or **26** on the body weight in male Wistar rats. The change in body weight in Wistar rats fed palatable diet or standard diet and in Wistar rats fed palatable diet or standard diet treated for 21 days with **6** (**A**,**B**) or **26** (**C**,**D**). Results are means ± SEM, *n* = 6. Multiple comparisons were performed by two-way ANOVA, Sidak post-hoc (**A**,**C**) or by one-way ANOVA, Sidak post-hoc (**B**,**D**); * *p* < 0.05, ** *p* < 0.01, *** *p* < 0.001 significant vs. control rats fed standard diet; ^ *p* < 0.05, ^^ *p* < 0.01 significant vs. control rats fed palatable diet.

Compounds **6** and **26**, representing the most potent 5-HT_6_R agents of both groups, A (**6**) and B (**26**), were evaluated with regard to their influence on the body weight of male Wistar rats within metabolic assays in vivo (Figure 5). Animals that were fed palatable feed, and treated with **6**, exhibited significantly less weight gain when compared to animals in the control group consuming a standard feed. From day 16 of the experiment, there was a significant difference between the groups. The body weight of rats treated with **6** having access to preferential feed differed significantly from the weight of control animals fed standard feed. Results are shown in Figure 3A,B.

Similarly, animals fed palatable feed and treated with **26** showed significantly less weight gain than animals in the control group consuming a preferential feed (Figure 5C,D), but there was a significant difference between the groups from day 12 of the experiment,. Additionally, the body weight of rats treated with **26** and having access to preferential feed did not differ significantly from the weight of control animals fed standard feed. No effects on body weight were noted in animals treated either with **6** or with **26** and consuming standard feed.

### 2.5. “Drug-Like” Properties

#### 2.5.1. Lipophilicity

**Figure 6:**
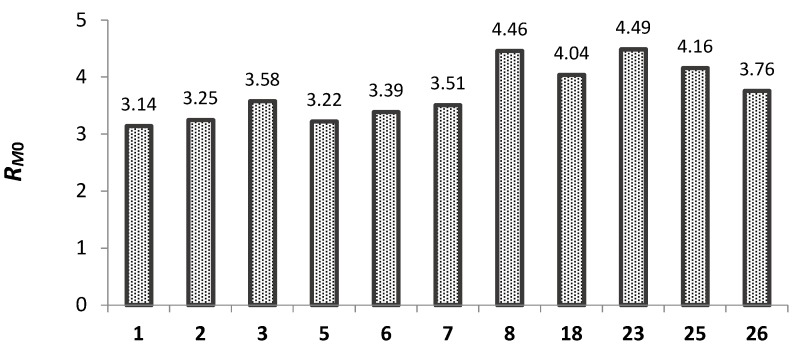
Lipophilicity parameters evaluated using R-TLC: *R_M_*_0_ values for compounds **1**–**3**, **5**–**8**, **18**, **23**, **25** and **26**.

The lipophilicity for compounds considered in the search for lead structures (**5**–**8**) in particular for the most active compounds found as derivatives of both lead modifications (**18**, **23**, **25** and **26**) was determined with the standard RP-TLC method [22]. For comparison, the active arylmethyl-triazine compounds previously found (**1**–**3**) were also examined. Stationary phase RP-18 and mixtures of water/methanol (1:9, *v*/*v*) as a mobile phase were employed. *R_M_* values were calculated from the *R_F_* data obtained. It was found that the *R_M_* parameters decreased linearly with increasing amounts of the organic modifier (methanol) in the mobile phases tested. The regression coefficients (*r*^2^) determined for all compounds were higher than 0.95. On the basis of the linear relationship between *R_M_* values and the volume fraction of methanol, values of *R_M_*_0_ in the systems analyzed corresponding to 100% of water were obtained by extrapolation. *R_M_*_0_ values received for the compounds tested ranged from 3.14 to 4.49 (Figure 6).

The most lipophilic character is associated with compound **23** (*R_M_*_0_ = 4.49), while the least lipophilic hydantoin compound is a weak 5-HT_6_R agent **5** (*R_M_*_0_ = 3.22). The arylmethyl derivatives previously investigated (**1**–**3**) displayed slightly less lipophilic properties than those of the most active hydantoin agents (**18**–**26**). Overall, these results indicate a good “drug-like” lipophilicity for the entire series of compounds investigated, taking into consideration either classical rules (Rules of Five, Ghose or Pfizer) or the latest CNS MPO approach [23].

#### 2.5.2. Toxicity In Vitro

**Figure 7:**
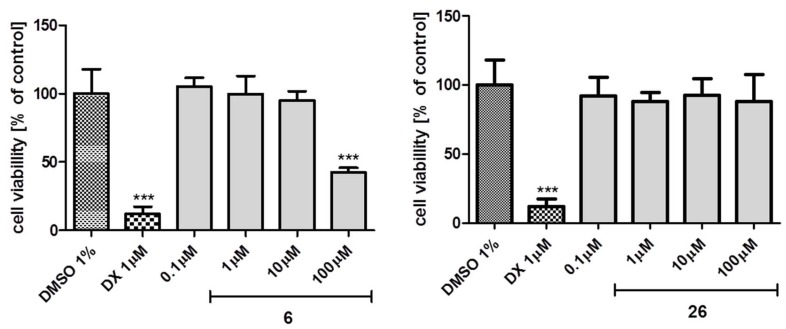
The influence of doxorubicin (DX) and 5-HT_6_R ligands on the viability of cultured HEK-293 cells. Statistical significance was evaluated by one-way ANOVA, followed by Bonferroni’s comparison test (*** *p* < 0.001 compared with the negative control).

The preliminary evaluation of the safety profile for the most promising 5-HT_6_R ligands **6** and **26** was performed in vitro with the HEK-293 eukaryotic cell line. The compounds examined were dissolved in culture growth media and incubated with cells for 72 h. Next, the MTS test was applied to determine the viability of the cells. As shown in Figure 7, a statistically significant decrease of viability (*** *p* < 0.001) was observed only for compound **6** and only at the highest concentration used, i.e., 100 μM. The reference cytostatic drug doxorubicin (DX) at the very low concentration 1 μM was also cytotoxic. Thus, the results obtained confirm a low or no significant toxicity for compounds **6** and **26**, respectively.

#### 2.5.3. Blood-Brain Barrier Permeability

In order to estimate an ability of the library synthetized (**4**–**26**) to penetrate the blood-brain barrier (BBB), the permeability QPlogBB parameter was calculated (Table 2) using QikProp from Schrodinger Suite 2017 [24]. QikProp predictions are for orally delivered drugs, and thus some natural neurotransmitters, e.g., dopamine and serotonin, are CNS negative, because they are extremely polar to cross the blood-brain barrier. Recommended values range for compounds that penetrate BBB is from −3.0 to 1.2. All the hydantoin-triazines investigated (**4**–**26**) are predicted as BBB-permeable (Table 2) with QPlogBB values from −0.98 (**25**) to −0.33 (**12**).

## 3. Computer-Aided SAR Analysis

In order to expand the current knowledge about structural properties responsible for 5-HT_6_R activity in the group of recently discovered aryl derivatives of 1,3,5-triazine, this study took into account the crucial role of the linker, which was found by comparative SAR analysis for the previously active arylmethyl- and inactive aryl-triazine molecules [13]. The new series of hydantoin-triazines (**5**–**26**) were designed as an extension of the methylene linker of the most active 2-arylmethyl-1,3,5-triaines, via an introduction of the inflexible, cyclic hydantoin insert, containing also hydrogen bond acceptors, and occurring in two topological variants found to be profitable in the preliminary lead identification (**4**–**8**). Both leads (**6** and **7**) and their derivatives (**9**–**18** and **19**–**26**, respectively) displayed significant submicromolar affinities for the target 5-HT_6_ receptors, much more potent than those of previously investigated linker-free aryl-triazines [13], but slightly less potent if compared to the arylmethyl ones (**1**–**3**, Figure 1). Interestingly, the influence of the topology of the aromatic moieties in the group of 1,3,5-triazine derivatives seems to be distinctly linker-dependent. Although the bulky aromatic area of the naphthalene was beneficial for all the groups considered, it was distinctly predominant in the case of the 5-arylhydantoin (**25**, **26** vs. **7** and **19**–**24**; Table 1), comparable with the 3-methylphenyl and 3-chlorophenyl compounds in the group arylmethyl triazines (**1**–**3**, Figure 1), and slightly less favourable than the 4-chlorophenyl substituent within the 5,5-dimethylhydantoin group (**18** vs. **6**, Table 1). Furthermore, the preferable *m*-substitution within the phenyl ring, found for the benzyl 1,3,5-triazine 5-HT_6_R agents earlier [13], has not been confirmed in this study, either for the 5,5-dimethyl-1-benzylhydantoin- (**6** vs. **10**) or for the 5-phenyl-5-methylhydantoin triazine compounds (**7** vs. **20**). Consequently, an influence of both variants of the hydantoin linkers (1,3,5-substitution, group A or 3,5-substitution, group B) on the 5-HT_6_R activity was dependent on the properties of the aromatic moiety. Thus, both variants appear to be comparable, although the most active compound (**26**) is a member of the 5-aryl-5-methylhydantoin derivatives (group B).

**Figure 8:**
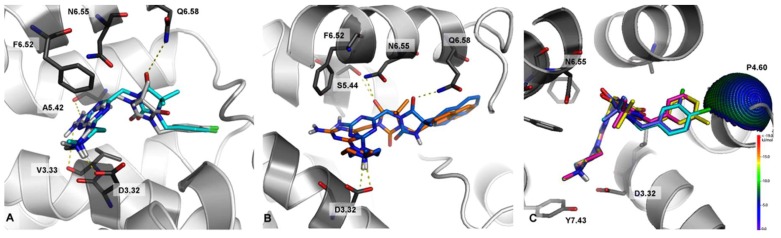
Molecular docking results of the representative library members for the 5-HT_6_ receptor. (**A**) Comparison of the binding mode of compounds **6** (cyan) and **7** (gray). (**B**) Binding mode of **18** (orange) and **26** (blue). (**C**) A superposition of the poses of compounds **9** (magenta), **17** (yellow), **6** (cyan), and **10** (violet) against the putative halogen binding pocket interaction spheres for the 5-HT_6_ receptor. The chlorine-oxygen theoretical interaction spheres illustrate the projected qualities of the formed ligand-receptor halogen bonds. Key residues in the binding site are presented as thick dark-grey sticks. The dotted yellow lines represent hydrogen bonds with polar residues.

Results of our docking studies have provided data useful to explain SAR on the molecular level (Figure 8).

In general, all of the newly synthesized derivatives of Lead 1 and Lead 2 exhibited a binding mode to 5-HT_6_R, which was very consistent with derivatives of 1,3,5-tiazines studied previously [13]. In addition to the crucial salt bridge interaction with D3.32, all of the docked ligands formed CH–π aromatic interactions with F6.52, and partially with F6.51. Additionally, the –NH_2_ group of the 1,3,5-triazine fragment was, usually, hydrogen-bonded with the carbonyl oxygen of A5.42 and V3.33 (Figure 8A). The methylene linker connecting 1,3,5-triazine and hydantoin rings, due to its tetrahedral conformation, forced the terminal aromatic group into a hydrophobic cavity formed by transmembrane helices 3–5 and extracellular loop 2. Thus, the structure-activity relationship in this series is determined by the different substituents at the terminal aromatic ring, and by the different orientation of hydantoin ring induced by its different positioning within the compound’s structure. A comparison of the analogues with different positions of the 1,3,5-triazine substitution at the hydantoin ring indicated that linking by the methylene group at position 3 of hydantoin is profitable (**6**, *K*_i_ = 127 nM), whereas a significant decrease of activity was noted for the derivative where the 1,3,5-triazine was linked with hydantoin via position 1 (**5**, *K*_i_ = 4275 nM). The analysis of the binding mode showed that a differently positioned hydantoin ring induces its different spatial orientation in the binding site (Figure 8A), and only the complex of **6** with the receptor is stabilized additionally by a hydrogen bond with a Q6.58 side chain.

Interestingly, within the group A and B, the best activity showed 2-naphthyl derivatives (**18** and **26**, respectively; Figure 8B). It should be noted, that apart from the length of the linker and the positioning of the hydantoin ring, the difference of activity between **18** and **26** is negligible—a comparison of its binding modes indicated that the most significant difference is noted for the hydantoin ring interaction. In both derivatives, the hydantoin ring is stabilized by hydrogen bonding, but due to its different orientation, the hydrogen bonds form with S5.44 and N6.55 or Q6.58 for **18** and **26**, respectively. It is worth to note that the shift of the chlorine atom from 3- to 4-position resulted in a 4- (for modification B) to 5-fold (for modification A) increase of affinity for 5-HT_6_R compared to its unsubstituted analogues. This relationship was confirmed by molecular docking (Figure 8C) revealing that only the presence of chlorine atom in 4-position stabilized the L–R complex through the formation of halogen bond with the carbonyl oxygen of P4.60 residue (Cl∙∙∙O distance = 3.31 Å, σ-hole angle = 151.47°). As the halogen bond appeared to possess a highly directional nature, to explain the decrease in activity by shifting the chlorine atom from position 3 to 4 in the phenyl ring, the interaction sphere [25,26] was plotted onto the relevant backbone carbonyl oxygens (Figure 8C). The 4-chloro substituent was positioned within the energetically favorable areas of the sphere, whereas the 3-chloro substituent pointed outside of the sphere, indicating no ability to form halogen bond.

## 4. Experimental Section

### 4.1. Chemistry

Reagents were purchased from Alfa Aesar (Karlsruhe, Germany) or Sigma Aldrich (Darmstadt, Germany). Methanol was dried over calcium oxide Reaction progress was verified using thin layer chromatography (TLC), which was carried out on 0.2 mm Merck silica gel 60 F254 plates. Spots were visualized by UV light or treatment with Dragendorff reagent. Melting points (m.p.) were determined using MEL-TEMP II apparatus (LD Inc., Long Beach, CA, USA) and are uncorrected. The ^1^H-NMR and ^13^C-NMR spectra were obtained on a Mercury-VX 300 Mz spectrometer (Varian, Palo Alto, CA, USA) in DMSO-*d*_6_ or CDCl_3_. Chemical shifts in ^1^H-NMR spectra were reported in parts per million (ppm) on the δ scale using the solvent signal as an internal standard. Data are reported as follows: Chemical shift, multiplicity (s, singlet; br.s., broad singlet; d, doublet; t, triplet; m, multiplet), coupling constant *J* in Hertz (Hz), number of protons, proton’s position (Ind–indole, Naph—naphthalene, Ph—phenyl, Pp—piperazine). LC-MS were carried out on a system Water TQ Detector (Water Corporation, Milford, CT, USA) consisting of a Waters Acquity UPLC, coupled to a Waters TQD mass spectrometer. Retention times (t_R_) are given in minutes. The UPLC/MS purity of all final compounds was determined (%).

Synthesis of compounds **27**, **29**, **50**–**58**, **63**, **65** and **68**–**70** was obtained by our [15,27] or other research groups [28,29,30,31,32] earlier and are reported in Chemical Abstract Database (see Supplementary Information).

#### 4.1.1. Synthesis of Methyl 2-(3-(4-chlorobenzyl)-5,5-dimethyl-2,4-dioxoimidazolidin-1-yl)acetate (**28**)

A mixture of 3-(4-chlorobenzyl)-5,5-dimethylimidazolidine-2,4-dione **27** (20 mmol, 5.05 g), TEBA (2.60 mmol, 0.60 g), K_2_CO_3_ (58 mmol, 8 g) in acetone (100 mL) was heated under reflux for 30 min. Methyl bromoacetate (20 mmol, 3.06 g) in acetone (20 mL) was added. The mixture was heated under reflux for 5 h and stirred at room temperature overnight. The inorganic precipitate was separated by filtration. The filtrate was evaporated, and the residue was crystallized with methanol. White solid, m.p. 78–80 °C, yield: 58% (11.60 mmol, 3.77 g), C_15_H_17_ClN_2_O_4_ (MW 324.76). ^1^H-NMR (300 MHz, DMSO-*d*_6_) δ = 7.38–7.41 (d def., 2H, Ar-3,5-H), 7.22–7.25 (d def., 2H, Ar-2,6-H), 4.57 (s, 2H, Ar-CH_2_), 4.18 (s, 2H, CH_2_COO), 3.68 (s, 3H, OCH_3_), 1.30 (s, 6H, 2× 5-CH_3_) ppm. LC/MS^+^: Purity: 98.40%, t_R_ = 6.14, (ESI) *m*/*z* [M + H]^+^ 325.11.

#### 4.1.2. General Procedure for the Synthesis of Alkyl 2-(1-arylmethyl-5,5-dimethyl-2,4-dioxoimidazolidin-3-yl)acetate (**30**–**40**)

A mixture of alkyl 2-(5,5-dimethyl-2,4-dioxoimidazolidin-3-yl)acetate **29** (12 mmol, **29a**: 2.40 g or **29b**: 2.58 g), TEBA (1.20 mmol, 0.36 g), K_2_CO_3_ (12 mmol, 4.80 g) in acetone (60 mL) was heated under reflux for 30 min. The appropriate arylmethyl halide (12 mmol) in acetone (15 mL) was added. The mixture was heated under reflux for 5–7 h and stirred at room temperature for further 24–48 h. The inorganic precipitate was separated by filtration. The filtrate was collected, the solvent then evaporated and the residue was dissolved in 30 mL of DCM and washed with 1% NaOH (2 × 30 mL) and H_2_O (2 × 30 mL). The organic phase was dried on anhydrous Na_2_SO_4_ overnight, separated from the drying agent and the solvent was evaporated. Then, Methods A or B were applied for purification. Method A: The residue was crystallized with EtOH and charcoal to give crystals of the intermediates. Method B: If Method A was applied, but no crystal appeared, the ethanolic solution was concentrated to give crude ester intermediates in glue form. No ester intermediate (**30**–**40**) was isolated in pure form (purity < 80%). The purity and identity of crude intermediates were evaluated using LC-MS. Taking into consideration the results of LC-MS a desirable ester was used for the synthesis of the final product in the amount suitable to its concentration in the crude product obtained (40–80%)

*Ethyl 2-(1-(4-chlorobenzyl)-5,5-dimethyl-2,4-dioxoimidazolidin-3-yl)acetate* (**30**). 1-Chloro-4-(chloromethyl)benzene (1.93 g) and **29a** were used. Method A. White crystals, m.p. 88–90 °C, yield: 68% (8.16 mmol, 2.65 g), C_15_H_17_ClN_2_O_4_ (MW 324.76). ^1^H-NMR (300 MHz, DMSO-*d*_6_) δ = 7.36–7.37 (d def., 4H, Ar), 4.52 (s, 2H, Ar-CH_2_), 4.25 (s, 2H, CH_2_COO), 3.70 (s, 3H, OCH_3_), 1.25 (s, 6H, 2× CH_3_) ppm. LC/MS^+^: Purity: 81%, t_R_ = 6.31, (ESI) *m*/*z* [M + H]^+^ 325.18.

*Ethyl 2-(1-benzyl-5,5-dimethyl-2,4-dioxoimidazolidin-3-yl)acetate* (**31**). Benzyl chloride (1.52 g) and **29a** were used. Method B. Glue mixture containing 44% of **31**, yield: 61.37%, C_15_H_18_N_2_O_4_ (MW 304.15). LC/MS^+^: Purity: 44%, t_R_ = 6.09, (ESI) *m*/*z* [M + H]^+^ 304.14.

*Ethyl 2-(1-(3-chlorobenzyl)-5,5-dimethyl-2,4-dioxoimidazolidin-3-yl)acetate* (**32**). 1-Chloro-3-(chloromethyl) benzene (1.93 g) and **29a** were used. Method B. Sticky liquid, yield: 85.22%, C_15_H_19_ClN_2_O_4_ (MW 338.59). ^1^H-NMR (300 MHz, DMSO-*d*_6_) δ = 7.41–7.33 (m, 1H, Ph-5-H), 7.31–7.28 (m, 3H, Ph-2,4,6-H), 4.54 (s, 2H, Ph-CH_2_), 4.23 (s, 2H, CH_2_COO), 4.10 (q, *J* = 7.05 Hz, 2H, CH_3_–CH_2_), 1.26 (s, 6H, 2× CH_3_), 1.16 (t, *J* = 7.35 Hz, 3H, CH_3_–CH_2_) ppm. LC/MS^+^: Purity: 67.03%, t_R_ = 6.73, (ESI) *m*/*z* [M + H]^+^ 339.59.

*Ethyl 2-(1-(2,5-dichlorobenzyl)-5,5-dimethyl-2,4-dioxoimidazolidin-3-yl) acetate* (**33**). 1-Bromo-2,5-(di-chloro methyl) benzene (3.60 g) and **29a** were used. Method A. yellowish Solid, m.p. 98 °C, yield: 98.21%, C_16_H_18_C_l2_N_2_O_4_ (MW 373.04). ^1^H-NMR (300 MHz, DMSO-*d*_6_) δ = 7.52–7.41 (m, 1H, Ph-6-H), 7.39–7.40 (m, 2H, Ph-3,4-H), 4.58 (s, 2H, Ph-CH_2_), 4.24 (s, 2H, CH_2_COO), 4.10 (q, *J* = 7.00 Hz, 2H,CH_3_–CH_2_), 1.29 (s, 6H, 2× CH_3_), 1.17 (d, *J* = 7.00 Hz, 3H, CH_3_–CH_2_) ppm. LC/MS^+^: Purity: 93.90%, t_R_ = 7.28, (ESI) *m*/*z* [M + H]^+^ 373.03.

*Ethyl 2-(1-(2,4-dichlorobenzyl)-5,5-dimethyl-2,4-dioxoimidazolidin-3-yl)acetate* (**34**). 1-Chloro-2,4-(di-chloro methyl) benzene (2.34 g) and **29a** were used. Method B. White Solid, m.p. 105 °C, yield: 91.52%, C_16_H_18_C_l2_N_2_O_4_ (MW 373.04). ^1^H-NMR (300 MHz, DMSO-*d*_6_) δ = 7.66 (d, *J* = 2.0 Hz, 1H, Ph-3-H), 7.47–7.37 (m, 2H-Ph-5,6-H), 4.60 (s, 2H-Ph-CH_2_), 4.26 (s, 2H, CH_2_COO), 4.16 (q, *J* = 7.1 Hz, 2H–CH_3_–CH_2_), 1.29 (s, 6H, 2× CH_3_), 1.21 (t, *J* = 7.1 Hz, 3H, CH_3_) ppm. ^13^C-NMR (75 MHz, DMSO-*d*_6_) δ = 175.83, 167.53, 154.84, 134.60, 133.22, 133.18, 130.71, 129.32, 127.98, 62.51, 61.83, 22.68, 14.38 ppm. LC/MS^+^: Purity: 91.74%, t_R_ = 7.52, (ESI) *m*/*z* [M + H]^+^ 374.04.

*Ethyl 2-(1-(4-fluorobenzyl)-5,5-dimethyl-2,4-dioxoimidazolidin-3-yl)acetate* (**35**). 4-Flouro benzyl chloride (2.17 g) and **29a** were used. Method B. Sticky liquid, yield: 90.72%, C_16_H_19_FN_2_O_4_ (MW 322.99). LC/MS^+^: Purity: 69.51%, t_R_ = 7.52, (ESI) *m*/*z* [M + H]^+^ 323.99.

*Ethyl 2-(1-(3-fluorobenzyl)-5,5-dimethyl-2,4-dioxoimidazolidin-3-yl)acetate* (**36**). 3-Flouro benzyl chloride (1.74 g) and **29a** were used. Method A. Sticky liquid, yield: 77.06%, C_16_H_19_FN_2_O_4_ (MW 322.99). ^1^H-NMR (300 MHz, DMSO-*d*_6_) δ = 7.41–7.36 (m, 1H, Ph-5-H), 7.19–7.13 (m, 3H, Ph-2,4,6-H), 4.55 (s, 2H, Ph-CH_2_), 4.23 (s, 2H, CH_2_COO), 4.10 (q, *J* = 7.05 Hz, 2H, CH_3_–CH_2_), 1.26 (s, 6H, 2× CH_3_), 1.16 (t, *J* = 7.05 Hz, 3H, CH_3_–CH_2_) ppm. LC/MS^+^: Purity: 69.43%, t_R_ = 6.23, (ESI) *m*/*z* [M + H]^+^ 323.99.

*Ethyl 2-(1-(3-bromobenzyl)-5,5-dimethyl-2,4-dioxoimidazolidin-3-yl)acetate* (**37**). 3-Bromo benzyl chloride (3.75 g) and **29a** were used. Method B. Sticky liquid, yield: 60%, C_16_H_19_BrN_2_O_4_ (MW 383.24). LC/MS^+^: Purity: 78.26%, t_R_ = 6.85, (ESI) *m*/*z* [M + H]^+^ 384.24.

*Ethyl 2-(1-(3-methoxybenzyl)-5,5-dimethyl-2,4-dioxoimidazolidin-3-yl)acetate* (**38**). 3-Methoxy benzyl chloride (1.88 g) and **29a** were used. Method B. Sticky liquid, yield: 68.94%, C_17_H_22_N_2_O_5_ (MW 334.38). LC/MS^+^: Purity: 88.03%, t_R_ = 6.65, (ESI) *m*/*z* [M + H]^+^ 335.38.

*Ethyl 2-(1-(3-methylbenzyl)-5,5-dimethyl-2,4-dioxoimidazolidin-3-yl)acetate* (**39**). 3-Methyl benzyl chloride (1.69 g) and **29a** were used. Method B. Sticky liquid, yield: 61.05%, C_17_H_22_N_2_O_4_ (MW 318.38). LC/MS^+^: Purity: 81.94%, t_R_ = 6.12, (ESI) *m*/*z* [M + H]^+^ 319.38.

*Ethyl 2-(1-(2-naphthyl)-5,5-dimethyl-2,4-dioxoimidazolidin-3-yl)acetate* (**40**). 2-(Chloromethyl)naphthalene (2.65 g) and **29a** were used. Method B. White solid, m.p. 95 °C, yield: 81.47%, C_20_H_22_N_2_O_4_ (MW 354.19). ^1^H-NMR (300 MHz, DMSO-*d*_6_) δ = 7.90–7.84 (m, 4H, naft-1,4,5,8-H), 7.52–7.45 (m, 3H, naft-3,6,7-H), 4.71 (s, 2H, Ph-CH_2_), 4.26 (s, 2H, CH_2_COO), 4.16–4.11 (q, *J* = 7.35 Hz, 2H, CH_3_–CH_2_), 1.26 (s, 6H, 2× CH_3_), 1.17(d, *J* = 7.00 Hz, 3H, CH_3_–CH_2_) ppm. LC/MS^+^: Purity: 90.56%, t_R_ = 7.08, (ESI) *m*/*z* [M + H]^+^ 355.15.

#### 4.1.3. General Procedures for the Synthesis of Methyl 2-(5-arylhydantoin-3-yl)acetates (**60**–**67** and **71**)

A mixture of a suitable arylhydantoin **50**–**58** (Scheme 1) or **70** (15–20 mmol) with K_2_CO_3_ (6–8 g), TEBA (0.45–0.60 g) in acetone (75–100 mL) was stirred and refluxed for 30 min. Then, the solution of methyl bromoacetate (15–20 mmol, 2.30–3.06 g) in acetone (15–20 mL) was added. The mixture was stirred and refluxed for 3–6 h, then, stirred at room temperature overnight. The inorganic components were separated by filtration. The filtrate was concentrated, and the residue was purified using the similar methods as those for compounds 30–40, but MeOH in place of EtOH was used for crystallization.

*Methyl 2-(5-methyl-2,4-dioxo-5-phenylimidazolidin-3-yl)acetate* (**60**). Compound **51** (2.85 g) and methyl bromoacetate (2.30 g) were used. Sticky liquid, yield: 48.6%, C_13_H_14_N_2_O_4_ (MW 262.26). ^1^H-NMR (300 MHz, DMSO-*d*_6_) δ = 9.06 (s, 1H, N1–H), 7.47–7.50 (d, *J* = 7.00 Hz, 2H, Ph-2,6-H), 7.33–7.42 (m, 3H Ph-3,4,5-H), 4.19 (s, 2H, N3–CH_2_), 3.67 (s, 3H, O-CH_3_), 1.70 (s, 3H, CH_3_) ppm. LC/MS^+^: Purity: 85.92%, t_R_ = 4.43, (ESI) *m*/*z* [M + H]^+^ 263.11.

*Methyl 2-(5-(3-chlorophenyl)-5-methyl-2,4-dioxoimidazolidin-3-yl)acetate* (**61**). Compound **52** (4.49g) and methyl bromoacetate (3.06 g) were used. White Chrystal, m.p. 102 °C, yield: 51.10%, C_13_H_13_ClN_2_O_4_ (MW 296.71). ^1^H-NMR (300 MHz, DMSO-*d*_6_) δ = 9.13 (s, 1H, N1–H), 7.51 (s, 1H, Ph-6-H), 7.43–7.47 (m, 3H, Ph-2,4,5-H), 4.20 (s, 2H, N3-CH_2_), 3.66 (s, 3H, O–CH_3_), 1.70 (s, 3H, CH_3_) ppm. LC/MS^+^: Purity: 100%, t_R_ = 5.25, (ESI) *m*/*z* [M + H]^+^ 297.07.

*Methyl 2-(5-(2,5-dichlorophenyl)-5-methyl-2,4-dioxoimidazolidin-3-yl)acetate* (**62**). Compound **53** (4.14 g) and methyl bromoacetate (2.45 g) were used. White solid, m.p. 214 °C, yield: 83.19%, C_13_H_12_C_l2_N_2_O_4_ (MW 331.15). ^1^H-NMR (300 MHz, DMSO-*d*_6_) δ = 8.75 (s, 1H, N1-H), 7.69 (s, 2H, Ph-4-H), 7.52 (s, 2H, Ph-3,6-H), 4.25 (s, 2H, N3–CH_2_), 3.68 (s, 3H, O–CH_3_), 1.80 (s, 3H, CH_3_) ppm. LC/MS^+^: Purity: 99.10%, t_R_ = 5.41, (ESI) *m*/*z* [M + H]^+^ 330.69.

*Methyl 2-(5-(2,3,4-trichlorophenyl)-5-methyl-2,4-dioxoimidazolidin-3-yl)acetate* (**64**). Compound **54** (5.18 g) and methyl bromoacetate (3.06 g) were used. White solid, m.p. 172 °C, yield: 37.76%, C_13_H_12_C_l2_N_2_O_4_ (MW 331.15). ^1^H-NMR (300 MHz, DMSO-*d*_6_) δ = 8.75 (s, 1H, N1–H), 7.68–7.71 (d, *J* = 8.8 Hz, 1H, Ph-3-H), 7.65–7.66 (s, 1H, Ph-5-H), 7.48–7.52 (m, 1H, Ph-6-H), 4.25 (s, 2H, N3–CH_2_), 3.68 (s, 3H, O–CH_3_), 1.78 (s, 3H, CH_3_) ppm. LC/MS^+^: Purity: 96.70%, t_R_ = 5.49, (ESI) *m*/*z* [M + H]^+^ 330.76.

*Methyl 2-(5-methyl-5-(naphthalen-1-yl)-2,4-dioxoimidazolidin-3-yl)acetate* (**66**). 5-Methyl-5-(naphthalen-1-yl)imidazolidine-2,4-dione **57** (20 mmol, 4.80 g), K_2_CO_3_ (8 g), TEBA (0.60 g) in acetone (100 mL) and methyl bromoacetate (20 mmol, 3.06 g) in acetone (20 mL) were stirred and refluxed for 6 h. Method B. White glue-crystals, m.p. 105–109 °C, yield: 78% (15.60 mmol, 4.86 g), C_17_H_16_N_2_O_4_ (MW 312.11). ^1^H-NMR (300 MHz, DMSO-*d*_6_) δ = 8.98 (s, 1H, NH), 7.94–8.01 (m, 3H, Ar-4,5,8), 7.75 (d, *J* = 7.44 Hz, 1H, Ar-2), 7.48–7.57 (m, 3H, Ar-3,6,7), 4.33–4.39 (m, 2H, –CH_2_CO), 3.73 (s, 3H, –OCH_3_), 1.94 (s, 3H, 5-CH_3_) ppm. LC/MS^+^: Purity: 65.46%, t_R_ = 5.47, (ESI) *m*/*z* [M + H]^+^ 312.95.

*Methyl 2-(5-methyl-5-(naphthalen-2-yl)-2,4-dioxoimidazolidin-3-yl)acetate* (**67**). 5-Methyl-5-(naphthalen-2-yl)imidazolidine-2,4-dione **58** (15 mmol, 3.60 g), K_2_CO_3_ (6 g), TEBA (0.45 g) in acetone (75 mL) and methyl bromoacetate (15 mmol, 2.30 g) in acetone (15 mL) were stirred and refluxed for 5 h. Method A. White crystals, m.p. 134–138 °C, yield: 58% (8.7 mmol, 2.72 g), C_17_H_16_N_2_O_4_ (MW 312.11). ^1^H-NMR (300 MHz, DMSO-*d*_6_) δ = 9.15 (s, 1H, NH), 8.02 (s, 1H, Ar-1-H), 7.60 (dd, *J*_1_ = 8.72 Hz, *J*_2_ = 1.80 Hz, 2H, Ar-6,7-H), 7.87–7.99 (m, 3H, Ar-4,5,8-H), 7.51–7.57 (m, 1H, Ar-3), 4.23 (s, 2H, –CH_2_CO), 3.66 (s, 3H, –OCH_3_), 1.81 (s, 3H, 5-CH_3_) ppm. LC/MS^+^: Purity: 76.61%, t_R_ = 5.63, (ESI) *m*/*z* [M + H]^+^ 312.82.

*Methyl 2-(5,5-bis(4-chlorophenyl)-2,4-dioxoimidazolidin-3-yl)acetate* (**71**). 5,5-*bis*(4-Chlorophenyl)-imidazolidin-2,4-dione **70** (15 mmol, 4.80 g), K_2_CO_3_ (6 g), TEBA (0.45 g) in acetone (75 mL) and methyl bromoacetate (15 mmol, 2.30 g) in acetone (15 mL) were stirred and refluxed for 4 h. Method A. Creamy crystals, m.p. 120–121 °C, yield: 62%, C_18_H_14_C_l2_N_2_O_4_ (MW 392.03). ^1^H-NMR (300 MHz, DMSO-*d*_6_) δ = 7.53 (d, *J* = 8.7 Hz, 4H-ph), 7.37 (d, *J* = 8.7 Hz, 4H-ph), 4.31 (s, 2H–CH_2_), 3.70 (s, 3H-CH_3_), 1.04 (d, *J* = 6.1 Hz, 1H-NH) ppm. LC/MS^+^: Purity: 91.91%, t_R_ = 7.26, (ESI) *m*/*z* [M + H]^+^ 393.10.

#### 4.1.4. General Procedure for the Synthesis of 1,3,5-Triazine Final Products (**5**–**26**)

Sodium (8–10 mmol) was dissolved in 10 mL of absolute methanol, then 4-methylpiperazin-1-yl biguanidine × 2HCl (4–5 mmol) and an appropriate ester **28**, **30**–**40**, **59**–**67** or **71** (4–5 mmol) were added. The reaction mixture was refluxed for 15–30 h. After cooling to room temperature, water (10 mL) was added and the mixture was stirred for 0.5 h. The triazine product precipitated (**5**–**26**) was separated and purified using Methods C, D or E. Method C: Crystallization with methanol. Method D: crystallization with methanol and charcoal. Method E: crystallization with ethanol.

*3-(4-Chlorobenzyl)-1-((4-amino-6-(4-methylpiperazin-1-yl)-1,3,5-triazin-2-yl)methyl)-5,5-dimethylimidazolidine-2,4-dione* (**5**). Ester **28** (4.30 mmol, 1.40 g) and 4-methylpiperazin-1-yl biguanidine × 2HCl (4.30 mmol, 1.11 g) were used (48 h refluxed). Method C. White solid, m.p. 166–169 °C, yield: 34% (0.67 g), C_21_H_27_N_8_O_2_Cl (MW 458.95). ^1^H-NMR (300 MHz, DMSO-*d*_6_) δ 7.34–7.52 (m, 2H, Ph-3,5-H), 7.21–7.32 (m, 2H, Ph-2,6-H), 6.82 (br. s., 2H, NH_2_), 4.55 (s, 2H, Ph-CH_2_), 4.19 (s, 2H, TR-CH_2_), 3.72–3.37 (br s, 4H, Pp-2,6-H), 2.15 (s, 7H, Pp-3,5-H + Pp-CH_3_), 1.31 (s, 6H, 2× CH_3_) ppm. ^13^C-NMR (75 MHz, DMSO-*d*_6_) δ = 176.83, 173.77, 167.04, 164.65, 154.86, 136.23, 132.52, 129.73, 129.03, 62.11, 54.67, 46.19, 44.19, 42.75, 41.08, 22.84 ppm. LC/MS^+^: Purity: 99.46%, t_R_ = 3.75, (ESI) *m*/*z* [M + H]^+^ 459.23.

*1-(4-Chlorobenzyl)-3-((4-amino-6-(4-methylpiperazin-1-yl)-1,3,5-triazin-2-yl)methyl)-5,5-dimethylimidazolidine-2,4-dione* (**6**). Ester **30** (4.30 mmol, 1.40 g) and 4-methylpiperazin-1-yl biguanidine × 2HCl (4.30 mmol, 1.11 g) were used (50 h refluxed). Method C. White solid, m.p. 204–205 °C, yield: 19% (0.38 g), C_21_H_27_N_8_O_2_Cl (MW 458.95). ^1^H-NMR (300 MHz, DMSO-*d*_6_) δ = 7.38 (s, 4H, Ph-2,3,5,6-H), 6.87 (br. s., 2H, NH_2_), 4.51 (s, 2H, Ph-CH_2_), 4.27 (s, 2H, TR-CH_2_), 3.55 (br. s., 4H, Pp-2,6-H), 2.15 (s, 7H, Pp-3,5-H + Pp-CH_3_), 1.26 (s, 6H, 2× CH_3_) ppm. LC/MS^+^: Purity: 100%, t_R_ = 3.94, (ESI) *m*/*z* [M + H]^+^ 459.23.

*3-((4-Amino-6-(4-methylpiperazin-1-yl)-1,3,5-triazin-2-yl)methyl)-5-(4-chlorophenyl)-5-methylimidazolidine-2,4-dione* (**7**). Ester **59** (5 mmol, 1.48 g) and 4-methylpiperazin-1-yl biguanidine × 2HCl (4.30 mmol, 1.11 g) were used (13.5 h refluxed). Method C. White solid, m.p. 218–220 °C, yield: 23% (0.42 g), C_19_H_23_N_8_O_2_Cl (MW 430.89). ^1^H-NMR (300 MHz, DMSO-*d*_6_) δ = 9.02 (s, 1H, Hyd-1-H), 7.51–7.69 (m, 2H, Ph-3,5-H), 7.36–7.51 (m, 2H, Ph-2,6-H), 6.83 (br. s., 2H, NH_2_), 4.21 (s, 2H, TR-CH_2_), 3.53 (br. s., 2H, Pp-2,6-He), 3.27–3.32 (m, 2H, Pp-2,6-Ha), 2.13 (s, 7H, Pp-3,5-H + Pp-CH_3_), 1.68 (s, 3H, Hyd-CH_3_) ppm. ^13^C-NMR (75 MHz, DMSO-*d*_6_) δ = 175.25, 171.94, 166.94, 164.54, 155.78, 139.22, 133.15, 128.87, 127.93, 62.99, 54.57, 46.16, 42.63, 42.45, 25.92 ppm. LC/MS^+^: Purity: 100%, t_R_ = 3.23, (ESI) *m*/*z* [M + H]^+^ 431.19.

*3-((4-Amino-6-(4-methylpiperazin-1-yl)-1,3,5-triazin-2-yl)methyl)-5,5-bis(4-chlorophenyl)imidazolidine-2,4-dione* (**8**). Ester **71** (4.80 mmol, 1.90 g) and 4-methylpiperazin-1-yl biguanidine × 2HCl (4 mmol, 1.03 g) were used (30 h refluxed). Method C. White solid, m.p. 266–268 °C, yield: 40% (0.83 g), C_24_H_24_N_8_O_2_Cl_2_ (MW 526.14). ^1^H-NMR (300 MHz, DMSO-*d*_6_) δ = 9.79 (s, 1H, Hyd-1-H), 7.34–7.59 (m, 8H, 2× Ph-2,3,5,6-H), 6.85 (br. s, 2H, NH_2_), 4.30 (s, 2H, TR-CH_2_), 3.55 (br. s, 2H, Pp-2,6-He), 3.09 (br. s, 2H, Pp-2,6-Ha), 1.76–2.35 (m, 7H, Pp-3,5-H + Pp-CH_3_) ppm. ^13^C-NMR (75 MHz, DMSO-*d*_6_) δ = 173.01, 171.74, 166.92, 164.52, 155.38, 138.75, 133.57, 129.12, 129.01, 68.59, 54.58, 46.16, 42.77, 42.53 ppm. LC/MS^+^: Purity: 100%, t_R_ = 3.23, (ESI) *m*/*z* [M]^+^ 527.16.

*3-((4-Amino-6-(4-methylpiperazin-1-yl)-1,3,5-triazin-2-yl)methyl)-1-benzyl-5,5-dimethylimidazolidine-2,4-dione* (**9**). Ester **31** (3 mmol, 2.03 g) and 4-methylpiperazin-1-yl biguanidine × 2HCl (3 mmol, 0.77 g) were used (36 h refluxed). Method D. White solid, m.p. 206 °C, yield: 3.94%, C_21_H_28_N_8_O_2_ (MW 424.23). ^1^H-NMR (300 MHz, DMSO-*d*_6_) δ = 7.36–7.25 (m, 5H-Ph), 6.87 (s, 2H, NH_2_), 4.52 (s, 2H, CH_2_), 4.28 (s, 2H, TR-CH_2_), 3.62 (s, 4H, Pp-2,6-H), 2.15–2.23 (m, 7H, Pp-3,5-H, Pp-CH_3_), 1.25 (m, 6H, 2× CH_3_) ppm. LC/MS^+^: Purity: 98.30%, t_R_ = 3.50, (ESI) *m*/*z* [M]^+^ 425.20.

*1-(3-Chlorobenzyl)-3-((4-amino-6-(4-methylpiperazin-1-yl)-1,3,5-triazin-2-yl)methyl)-5,5-dimethylimidazolidine-2,4-dione* (**10**). Ester **32** (5 mmol, 2.52 g) and 4-methylpiperazin-1-yl biguanidine × 2HCl (5 mmol, 0.92 g) were used (6 h refluxed), and stirred for another 24 h in a room temperature. Method D. White solid, m.p. 168 °C, yield: 24.81%, C_21_H_28_N_8_O_2_ (MW 458.19). ^1^H-NMR (300 MHz, DMSO-*d*_6_) δ = 7.42 (s, 1H, Ph-6-H), 7.33 (m, 3H, Ph-2,4,5-H), 6.86 (s, 2H, NH_2_), 4.52 (s, 2H–CH_2_), 4.27(s, 2H, TR-CH_2_), 3.58 (s, 4H, Pp-2,6-H), 2.13 (m, 7H, Pp-3,5-H, Pp-CH_3_), 1.27 (s, 6H, 2× CH_3_) ppm. LC/MS^+^: Purity: 96.12%, t_R_ = 5.34, (ESI) *m*/*z* [M]^+^ 459.23.

*1-(2,5-Dichlorobenzyl)-3-((4-amino-6-(4-methylpiperazin-1-yl)-1,3,5-triazin-2-yl)methyl)-5,5-dimethylimidazolidine-2,4-dione* (**11**). Ester **33** (5 mmol, 2.01 g) and 4-methylpiperazin-1-yl biguanidine × 2HCl (5 mmol, 0.92 g) were used (6 h refluxed), and stirred for another 144 h in room a temperature. Method D. White solid, m.p. 163 °C, yield: 27.32%, C_21_H_26_C_l2_N_8_O (MW 492.16). ^1^H-NMR (300 MHz, DMSO-*d*_6_) δ = 7.53–7.50 (s, 1H, Ph-4-H), 7.41–7.38 (m, 2H, Ph-3,6-H), 6.84 (s, 2H, NH_2_), 4.58 (s, 2H, CH_2_), 4.29 (s, 2H, TR-CH_2_), 3.63 (s, 4H, Pp-2,6-H), 2.23–2.15 (m, 7H, Pp-3,5-H, Pp-CH_3_), 1.30 (s, 6H, 2× CH_3_) ppm. LC/MS^+^: Purity: 100%, t_R_ = 4.40, (ESI) *m*/*z* [M]^+^ 493.13.

*1-(2,4-Dichlorobenzyl)-3-((4-amino-6-(4-methylpiperazin-1-yl)-1,3,5-triazin-2-yl)methyl)-5,5-dimethylimidazolidine-2,4-dione* (**12**). Ester **34** (5 mmol, 2.03 g) and 4-methylpiperazin-1-yl biguanidine × 2HCl (5 mmol, 0.92 g) were used (3 h refluxed), and stirred for another 24 h in room a temperature. Method D. White solid, m.p. 123 °C, yield: 23.98%, C_21_H_26_C_l2_N_8_O (MW 492.16). ^1^H-NMR (300 MHz, DMSO-*d*_6_) δ = 7.65–7.64 (s, 1H, Ph-6-H), 7.40–7.41 (m, 2H, Ph-3,5-H), 6.88 (s, 2H, NH_2_), 4.58 (s, 2H, CH_2_), 4.28 (s, 2H, TR-CH_2_), 3.62 (s, 4H, Pp-2,6-H), 2.52–2.26 (m, 7H, Pp-3,5-H, Pp-CH3), 1.29 (s, 6H, 2× CH_3_) ppm. LC/MS^+^: Purity: 100%, t_R_ = 4.56, (ESI) *m*/*z* [M]^+^ 493.13.

*1-(4-Fluorobenzyl)-3-((4-amino-6-(4-methylpiperazin-1-yl)-1,3,5-triazin-2-yl)methyl)-5,5-dimethylimidazolidine-2,4-dione* (**13**). Ester **35** (5 mmol, 2.32 g) and 4-methylpiperazin-1-yl biguanidine × 2HCl (5 mmol, 0.92 g) were used (10 h refluxed), and stirred for another 24 h in room a temperature. Method D. White solid, m.p. 180 °C, yield: 9.23%, C_21_H_27_FN_8_O_2_ (MW 442.22). ^1^H-NMR (300 MHz, DMSO-*d*_6_) δ = 7.38–7.43 (m, 2H, Ph-2,6-H), 7.11–7.17 (m, 2H, Ph-3,5-H), 6.86 (s, 2H, NH_2_), 4.51 (s, 2H, CH_2_), 4.27 (s, 2H, TR-CH_2_), 3.57 (s, 4H, Pp-2,6-H), 2.14–2.23 (m, 7H, Pp-3,5-H, Pp-CH_3_), 1.26 (m, 6H, 2× CH_3_) ppm. LC/MS^+^: Purity: 100%, t_R_ = 3.68, (ESI) *m*/*z* [M]^+^ 443.28.

*1-(3-Fluorobenzyl)-3-((4-amino-6-(4-methylpiperazin-1-yl)-1,3,5-triazin-2-yl)methyl)-5,5-dimethylimidazolidine-2,4-dione* (**14**)**.** Ester **36** (5 mmol, 2.34 g) and 4-methylpiperazin-1-yl biguanidine × 2HCl (5 mmol, 0.92 g) were used (10 h refluxed), and stirred for another 24 h in room a temperature. Method D. White solid, m.p. 207 °C, yield: 9.32%, C_21_H_27_FN_8_O_2_ (MW 442.22). ^1^H-NMR (300 MHz, DMSO-*d*_6_) δ = 7.34 (m, 1H, Ph-6-H), 7.13 (m, 3H, Ph-2,4,5-H), 6.86 (s, 2H, NH_2_), 4.53 (s, 2H, CH_2_), 4.27 (s, 2H, TR-CH_2_), 3.58 (s, 4H, Pp-2,6-H), 2.14 (m, 7H, Pp-3,5-H, Pp-CH_3_), 1.27 (s, 6H, 2× CH_3_) ppm. LC/MS^+^: Purity: 100%, t_R_ = 3.59, (ESI) *m*/*z* [M]^+^ 443.22.

*1-(3-Bromobenzyl)-3-((4-amino-6-(4-methylpiperazin-1-yl)-1,3,5-triazin-2-yl)methyl)-5,5-dimethylimidazolidine-2,4-dione* (**15**). Ester **37** (5 mmol, 2.51 g) and 4-methylpiperazin-1-yl biguanidine × 2HCl (5 mmol, 0.92 g) were used (4 h refluxed), and stirred for another 24 h in room a temperature. Method D. White powder, m.p. 125 °C, yield: 56%, C_21_H_27_BrN_8_O_2_ (MW 502.14). ^1^H-NMR (300 MHz, DMSO-*d*_6_) δ = 7.59 (s, 1H, Ph-4-H), 7.51–7.44 (m, 1H, Ph-2-H), 7.38 (d, *J* = 7.8 Hz, 1H, Ph-6-H), 7.30 (t, *J* = 7.7 Hz, 1H, Ph-5-H), 6.88 (s, 2H, NH_2_), 4.54 (s, 2H, CH_2_), 4.29 (s, 2H, TR-CH_2_), 3.77–3.44 (m, 4H, Pp-2,6-H), 2.16 (m, 7H, Pp-3,5-H, Pp-CH_3_), 1.29 (s, 6H, 2× CH_3_) ppm. LC/MS^+^: Purity: 97.66%, t_R_ = 4.07, (ESI) *m*/*z* [M]^+^ 505.16.

*1-(3-Methoxybenzyl)-3-((4-amino-6-(4-methylpiperazin-1-yl)-1,3,5-triazin-2-yl)methyl)-5,5-dimethylimidazolidine-2,4-dione* (**16**). Ester **38** (5 mmol, 2.42 g) and 4-methylpiperazin-1-yl biguanidine × 2HCl (5 mmol, 0.92 g) were used (10 h refluxed), and stirred for another 24 h in room a temperature. Method D. White solid, m.p. 111 °C, yield: 13.79%, C_22_H_30_N_8_O_3_ (MW 454.24). ^1^H-NMR (300 MHz, DMSO-*d*_6_) δ = 7.25–7.20 (m, 1H, Ph-3-H), 6.93 (m, 2H, Ph-2,4-H), 6.84–6.82 (m, 1H, Ph-6-H, 2H, NH_2_), 4.49 (s, 2H, CH_2_), 4.28 (s, 2H, TR-CH_2_), 3.71 (s, 3H, CH_3_), 3.59 (s, 4H, Pp-2,6-H), 2.15(m, 7H, Pp-3,5-H, Pp-CH_3_), 1.25 (m, 6H, 2× CH_3_) ppm. LC/MS^+^: Purity: 97.60%, t_R_ = 3.55, (ESI) *m*/*z* [M]^+^ 455.24.

*1-(3-Methylbenzyl)-3-((4-amino-6-(4-methylpiperazin-1-yl)-1,3,5-triazin-2-yl)methyl)-5,5-dimethylimidazolidine-2,4-dione* (**17**). Ester **39** (5 mmol, 2.61 g) and 4-methylpiperazin-1-yl biguanidine × 2HCl (5 mmol, 0.92 g) were used (5 h refluxed) and stirred for another 24 h in room a temperature. Method D. White solid, m.p. 154 °C, yield: 13.79%, C_22_H_30_N_8_O (MW 438.25). ^1^H-NMR (300 MHz, DMSO-*d*_6_) δ = 7.22–7.07 (m, 4H, Ph), 6.86 (s, 2H, NH_2_), 4.48 (s, 2H, CH_2_), 4.27 (s, 2H, TR-CH_2_), 3.59 (s, 4H, Pp-2,6-H), 2.27–2.15(m, 10H, Pp-3,5-H, Pp-CH_3_, Ph-CH_3_), 1.25 (s, 6H, 2× CH_3_) ppm. LC/MS^+^: Purity: 98.88%, t_R_ = 3.94, (ESI) *m*/*z* [M]^+^ 439.23.

*3-((4-Amino-6-(4-methylpiperazin-1-yl)-1,3,5-triazin-2-yl)methyl)-5,5-dimethyl-1-(naphthalen-1-ylmethyl)imidazolidine-2,4-dione* (**18**). Ester **40** (5 mmol, 1.96 g) and 4-methylpiperazin-1-yl biguanidine × 2HCl (5 mmol, 0.92 g) were used (5 h refluxed) and stirred for another 24 h in room a temperature. Method D. White powder, m.p. 116 °C, yield: 13.79%, C_25_H_30_N_8_O_2_ (MW 474.25). ^1^H-NMR (300 MHz, DMSO-*d*_6_) δ = 7.88–7.84 (m, 4H, naft-1,4,5,8-H), 7.50–7.48 (m, 3H, naft-3,6,7-H), 6.86 (s, 2H, NH_2_), 4.70 (s, 2H, CH_2_), 4.30 (s, 2H, TR-CH_2_), 3.55 (s, 4H, Pp-2,6-H), 2.22–2.02 (m, 7H, Pp-3,5-H, Pp-CH_3_), 1.29 (s, 6H, 2× CH_3_) ppm. LC/MS^+^: Purity: 100%, t_R_ = 4.31, (ESI) *m*/*z* [M]^+^ 475.25.

*3-((4-Amino-6-(4-methylpiperazin-1-yl)-1,3,5-triazin-2-yl)methyl)-5-methyl-5-phenylimidazolidine-2,4-dione* (**19**). Ester **60** (5 mmol, 1.31g) and 4-methylpiperazin-1-yl biguanidine × 2HCl (5 mmol, 0.92 g) were used (4.5 h refluxed) and stirred for another 24 h in room a temperature. Method C. White solid, m.p. 270 °C, yield: 50%, C_19_H_24_N_8_O_2_ (MW 396.45). ^1^H-NMR (300 MHz, DMSO-*d*_6_) δ = 9.00 (s, 1H, N1-H), 7.54–7.56 (d, *J* = 7.5 Hz, 2H, Ph-2,6-H), 7.39–7.43 (t, *J* = 7.25 Hz, Ph-3,5-H), 7.33–7.36 (d, *J* = 7.5 Hz,1H, Ph-4-H), 6.85 (s, 2H, NH_2_), 4.24 (s, 2H, N3-CH_2_), 3.61 (s, 2H, Pp-2,6-Hb), 3.40–3.50 (m, 2H, Pp-2,6-Ha), 2.16–2.24 (s, 4H, Pp-3,5-H), 2.16 (s, 3H, N–CH_3_), 1.73 (s, 3H, 5-CH_3_) ppm. ^13^C-NMR (75 MHz, DMSO-*d*_6_) δ = 175.62, 172.08, 166.96, 164.61, 155.92, 140.14, 128.88, 128.31, 125.98, 63.40, 54.64, 46.20, 42.72, 42.47, 25.82 ppm. LC/MS^+^: Purity: 96.38%, t_R_ = 2.62, (ESI) *m*/*z* [M]^+^ 397.22.

*3-((4-Amino-6-(4-methylpiperazin-1-yl)-1,3,5-triazin-2-yl)methyl)-5-(3-chlorophenyl)-5-methylimidazolidine-2,4-dione* (**20**). Ester **61** (5 mmol, 1.49 g) and 4-methylpiperazin-1-yl biguanidine × 2HCl (5 mmol, 0.92 g) were used (5 h refluxed). Method C. White solid, m.p. 234 °C, yield: 56.80%, C_19_H_23_ClN_8_O_2_ (MW 430.89). ^1^H-NMR (300 MHz, DMSO-*d*_6_) δ = 9.00 (s, 1H, N–H), 7.54–7.58 (m, 2H, Ph-2,6-H), 7.45–7.49 (m, 2H, Ph-4,5-H), 6.85 (s, 2H, NH_2_), 4.25 (s, 2H, N3–CH_2_), 3.60 (s, 2H, Pp-2,6-Hb), 3.30 (s, 2H, Pp-2,6-Ha), 2.26 (s, 2H, Pp-3,5-Hb), 2.16 (s, 3H, N–CH_3_), 2.09 (s, 2H, Pp-3,5-Ha), 1.72 (s, 3H, 5-CH_3_) ppm. ^13^C-NMR (75 MHz, DMSO-*d*_6_) δ = 175.08, 171.94, 166.96, 164.55, 155.74, 142.65, 133.63, 130.92, 128.38, 125.80, 124.85, 63.11, 54.60, 46.19, 42.67, 42.50, 26.21 ppm. LC/MS^+^: Purity: 100%, t_R_ = 3.23, (ESI) *m*/*z* [M]^+^ 431.12.

*3-((4-Amino-6-(4-methylpiperazin-1-yl)-1,3,5-triazin-2-yl)methyl)-5-(2,5-dichlorophenyl)-5-methylimidazolidine-2,4-dione* (**21**). Ester **62** (5 mmol, 1.65 g) and 4-methylpiperazin-1-yl biguanidine × 2HCl (5 mmol, 0.92 g) were used (3.5 h refluxed). Method C. White solid, m.p. 166 °C, yield: 79.10%, C_19_H_22_C_l2_N_8_O_2_ (MW 465.34). ^1^H-NMR (300 MHz, DMSO-*d*_6_) δ = 8.66 (s, 1H, N1–H), 7.72 (s, 1H, Ph-4-H), 7.54 (s, 2H, Ph-3,6-H), 6.89 (s, 2H, NH_2_), 4.30 (s, 2H, N3–CH_2_), 3.67 (m, 4H, Pp-2,6-H), 2.29 (s, 4H, Pp-3,5-H), 2.19 (s, 3H, N–CH_3_), 1.87 (s, 3H, 5-CH_3_) ppm. ^13^C-NMR (75 MHz, DMSO-*d*_6_) δ = 174.90, 172.31, 167.02, 164.70, 156.23, 137.56, 133.13, 132.49, 132.26, 130.68, 130.05, 62.65, 54.71, 46.21, 42.90, 42.49, 25.18 ppm. LC/MS^+^: Purity: 99.11%, t_R_ = 3.53, (ESI) *m*/*z* [M]^+^ 465.15.

*3-((4-Amino-6-(4-methylpiperazin-1-yl)-1,3,5-triazin-2-yl)methyl)-5-(2,4-dichlorophenyl)-5-methylimidazolidine-2,4-dione* (**22**). Ester **63** (5 mmol, 1.66 g) and 4-methylpiperazin-1-yl biguanidine × 2HCl (5 mmol, 0.92 g) were used (4.5 h refluxed). Method C. White solid, m.p. 228 °C, yield: 76.56%, C_19_H_22_C_l2_N_8_O_2_ (MW 465.34). ^1^H-NMR (300 MHz, DMSO-*d*_6_) δ = 8.65 (s, 1H, N1–H), 7.71–7.73 (d, 1H, Ph-3-H), 7.67–7.68 (s, 1H, Ph-5-H), 7.50–7.53 (d, 1H, Ph-6-H), 6.89 (s, 2H, NH_2_), 4.30 (s, 2H, N3–CH_2_), 3.67 (s, 4H, Pp-2,6-H), 2.29 (s, 4H, Pp-3,5-H), 2.19 (s, 3H, N–CH_3_), 1.86 (s, 3H, 5-CH_3_) ppm. ^13^C-NMR (75 MHz, DMSO-*d*_6_) δ = 175.12, 172.34, 167.02, 164.70, 156.25, 134.70, 134.66, 134.57, 131.70, 130.83, 127.80, 62.49, 54.71, 46.21, 42.88, 42.47, 25.29 ppm. LC/MS^+^: Purity: 100%, t_R_ = 3.66, (ESI) *m*/*z* [M]^+^ 465.08.

*3-((4-Amino-6-(4-methylpiperazin-1-yl)-1,3,5-triazin-2-yl)methyl)-5-(2,3,4-trichlorophenyl)-5-methylimidazolidine-2,4-dione* (**23**). Ester **64** (5 mmol, 1.83 g) and 4-methylpiperazin-1-yl biguanidine × 2HCl (5 mmol, 0.92 g) were used (4 h refluxed). Method C. White crystal, m.p. 256 °C, yield: 83.68%, C_19_H_21_C_l3_N_8_O_2_ (MW 499.78). ^1^H-NMR (300 MHz, DMSO-*d*_6_) δ = 7.74 (s, 2H, Ph-5,6-H), 6.89 (s, 2H, NH_2_), 4.32 (s, 2H, N3–CH_2_), 3.67 (s, 4H, Pp-2,6-H), 2.29 (s, 4H, Pp-3,5-H), 2.19 (s, 3H, N–CH_3_), 1.88 (s, 3H, 5-CH_3_) ppm. ^13^C-NMR (75 MHz, DMSO-*d*_6_) δ = 175.94, 172.32, 167.01, 164.70, 156.33, 136.85, 133.94, 133.58, 132.24, 129.48, 129.09, 63.11, 54.71, 46.21, 42.90, 42.59, 25.56 ppm. LC/MS^+^: Purity: 98.93%, t_R_ = 4.07, (ESI) *m*/*z* [M]^+^ 499.05.

*3-((4-Amino-6-(4-methylpiperazin-1-yl)-1,3,5-triazin-2-yl)methyl)-5-(4-methylphenyl)-5-methylimidazolidine-2,4-dione* (**24**). Ester **65** (5 mmol, 1.38 g) and 4-methylpiperazin-1-yl biguanidine × 2HCl (5 mmol, 0.92 g) were used (4.5 h refluxed). Method C. White solid, m.p. 240 °C, yield: 49.27%, C_20_H_26_N_8_O_2_ (MW 410.47). ^1^H-NMR (300 MHz, DMSO-*d*_6_) δ = 7.41–7.43 (d, *J* = 8.3 Hz, 2H, Ph-2,6), 7.20–7.22 (d, *J* = 7.9 Hz, 2H, Ph-3,5-H), 6.84 (s, 2H, NH_2_), 4.23 (s, 2H, N3–CH_2_), 3.59 (s, 4H, Pp-2,6-H), 2.30 (s, 3H, Ph-CH_3_), 2.16 (s, 4H, Pp-3,5-H), 2.10 (s, 3H, N–CH_3_), 1.69 (s, 3H, 5-CH_3_) ppm. ^13^C-NMR (75 MHz, DMSO-*d*_6_) δ = 175.74, 172.10, 166.96, 164.61, 155.91, 137.49, 137.29, 129.39, 125.87, 63.21, 54.65, 46.20, 42.70, 42.44, 25.83, 21.03 ppm. LC/MS^+^: Purity: 98.31%, t_R_ = 3.10, (ESI) *m*/*z* [M]^+^ 411.18.

*3-((4-Amino-6-(4-methylpiperazin-1-yl)-1,3,5-triazin-2-yl)methyl)-5-methyl-5-(naphthalen-1-yl)imidazolidine-2,4-dione* (**25**). Ester **66** (5 mmol, 1.56 g) and 4-methylpiperazin-1-yl biguanidine × 2HCl (5 mmol, 0.92 g) were used (5 h refluxed). Method E. White solid, m.p. 208 °C, yield: 32.87%, C_23_H_26_N_8_O_2_ (MW 446.50). ^1^H-NMR (300 MHz, DMSO-*d*_6_) δ = 8.95 (s, 1H, N1-H), 8.05–8.07 (m, 1H, Naft-8-H), 7.96–8.00 (t, 2H, Naft-4,5-H), 7.76–7.78 (d, *J* = 7.5 Hz; 1H, Naft-2-H), 7.51–7.55 (t, *J* = 7.7 Hz, 3H, Naft-3,6,7-H), 6.90 (s, 2H, NH_2_), 4.40 (s, 2H, N3–CH_2_), 3.63 (s, 4H, Pp-2,6-H), 2.17 (s, 4H, Pp-3,5-H), 2.07 (s, 3H, N–CH_3_), 2.02 (s, 3H, 5-CH_3_) ppm. ^13^C-NMR (75 MHz, DMSO-*d*_6_) δ = 176.31, 172.32, 167.07, 164.73, 156.03, 134.61, 134.21, 130.87, 130.22, 129.62, 126.92, 126.43, 126.10, 125.43, 125.11, 64.18, 54.66, 46.20, 42.88, 42.71, 26.74 ppm. LC/MS^+^: Purity: 99.06%, t_R_ = 3.55, (ESI) *m*/*z* [M]^+^ 447.20.

*3-((4-Amino-6-(4-methylpiperazin-1-yl)-1,3,5-triazin-2-yl)methyl)-5-methyl-5-(naphthalen-2-yl)imidazolidine-2,4-dione* (**26**). Ester **67** (5 mmol, 1.56 g) and 4-methylpiperazin-1-yl biguanidine × 2HCl (5 mmol, 0.92 g) were used (3 h refluxed). Method E. White solid, m.p. 144 °C, yield: 34.57%, C_23_H_26_N_8_O_2_ (MW 446.50). ^1^H-NMR (300 MHz, DMSO-*d*_6_) δ = 9.12 (s, 1H, N1–H), 8.07 (s, 1H, Naft-1-H), 7.93–7.99 (m, 3H, Naft-4,5,8-H), 7.72–7.74 (d def., 1H, Naft-3-H), 7.54–7.57 (m, 2H, Naft-6,7-H), 6.85 (s, 2H, NH_2_), 4.27 (s, 2H, N3–CH_2_), 3.40–3.60 (m, 2H, Pp-2,6-Hb), 3.05–3.20 (m, 2H, Pp-2,6-Ha), 2.09 (s, 4H, Pp-3,5-H), 2.00 (s, 3H, N–CH_3_), 1.83 (s, 3H, 5-CH_3_) ppm. ^13^C-NMR (75 MHz, DMSO-*d*_6_) δ = 175.56, 172.01, 166.95, 164.53, 155.92, 137.75, 132.96, 132.81, 128.62, 128.57, 127.89, 126.95, 126.90, 124.51, 124.33, 63.56, 53.90, 46.00, 42.56, 42.47, 25.94 ppm. LC/MS^+^: Purity: 99.11%, t_R_ = 3.49, (ESI) *m*/*z* [M]^+^ 447.20.

### 4.2. Molecular Modeling

#### 4.2.1. Optimization of the 5-HT_6_R Binding Site Using Induced-Fit Docking Procedure

The 5-HT_6_R homology model (build on β_2_ adrenergic template) was optimized using the induced-fit docking (IFD) [33] protocol from Schrödinger. The IFD combines flexible ligand docking, using the Glide algorithm [34], with receptor structure prediction and side chain refinement in Prime. The structures of Lead 1 and Lead 2 were used as inputs to IFD. In each case, the centroid of the grid box was anchored on D3.32 and allowed on residues refinement within 12 Å of ligand poses. The ten top-scored L–R complexes were inspected visually to select those showing the closest compliance with the common binding mode for monoaminergic receptor ligands [35]. The final validation of the receptor conformations selected was performed by docking (Glide SP mode) the synthetized library, retaining only those with a coherent binding mode for the whole set.

#### 4.2.2. Molecular Docking

The 5-HT_6_R models selected in IFD procedure were used to study the binding mode of the library synthesized. 3-dimensional structures of the ligands were prepared using LigPrep v3.6 (Schrödinger, New York, NY, USA) [36], and the appropriate ionization states at pH = 7.4 ± 1.0 were assigned using Epik v3.4 (Schrödinger, New York, NY, USA) [37]. Compounds with unknown absolute configuration were docked in all configurations. The Protein Preparation Wizard was used to assign the bond orders, appropriate amino acid ionization states and to check for steric clashes. The receptor grid was generated (OPLS3 force field [38]) by centering the grid box with a size of 12 Å on the D3.32 side chain. Automated flexible docking was performed using Glide at SP level [39].

#### 4.2.3. Plotting Interaction Spheres for Halogen Bonding

The halogen bonding web server was used (accessed on 22 February 2018, http://www.halogenbonding.com/) to visualize (plotting interaction spheres) the potential contribution of halogen bonding to ligand–receptor complexes.

### 4.3. Lipophilicity Study

#### 4.3.1. Thin-Layer Chromatography

The mobile phases were prepared by mixing the respective amounts of water and organic modifier (methanol) in a range from 40 to 90% (*v*/*v*) in 5% increments. TLC was performed on Silica gel 60RP-18 F_254_ plates (7 × 10 cm) plates (Merck, Darmstadt, Germany). Methanol was used to prepare the solutions of the substances. Solutions (10 µL) of the analyzed compounds were applied to the plates as 5 mm bands, 10 mm apart and 10 mm from the lower edge and sides of the plates, by using Linomat V applicator (Camag, Basel, Switzerland). The vertical chamber (Sigma–Aldrich, St. Louis, MI, USA), 20 × 10 × 18 cm in size, was saturated with mobile phase for 20 min. Development was carried out over 9 cm from the starting line at a temperature of 20 °C. Next, the plates were dried at room temperature, and the spots were observed under ultraviolet light at 254 nm and/or 366 nm (UV lamp, Camag, Basel, Switzerland). In each case, sharp and symmetric spots without a tendency for tailing were obtained. Each experiment was run in triplicate and mean *R_F_* (retardation factor) values were calculated.

Starting from the *R_F_* values, the *R_M_* parameters were computed as described in the formula:

*R_M_* = log(1/*R_F_* − 1)

The linear correlations between the *R_M_* values of the substances and the concentration of methanol in mobile phases were calculated for each compound with the Soczewiński-Wachtmeister equation [22]:

*R_M_* = *R_M_*_0_ + aC

where C is the concentration of the organic modifier (in %) in the mobile phase, a is the slope, and *R_M_*_0_ is the concentration of organic modifier extrapolated to zero.

#### 4.3.2. Statistical Methods

Statistical analysis was performed using Statistica v10 software (StatSoft, Tulsa, OK, USA). The correlation coefficients (*r*, *r*^2^), and the standard errors of the slope, interception, and estimate (S_a_, S_b_, S_e_) were used as the basis for testing the linearity of regression plots.

### 4.4. Studies In Vitro

#### 4.4.1. Radioligand Binding Assay

Radioligand binding assays were used to determine the affinity of the synthesized compounds for human serotonin 5-HT_6_R, which were stably expressed in HEK-293 cells. This procedure was accomplished via the displacement of [3H]-LSD (85.2 Ci mmol^−1^) for 5-HT_6_R. Each compound was tested in triplicate at 7 to 8 different concentrations (10^−11^ to 10^−4^ M). The inhibition constants (*K*_i_) were calculated using the Cheng–Prusoff equation [40], and the results are expressed as the mean of at least two independent experiments.

#### 4.4.2. Toxicity In Vitro Test

The ATCC CRL 1573 HEK-293 cells were seeded in 96-well plates (NuncTM) at a concentration of 1 × 104 cells/well in 200 μL in Dulbecco’s Modified Eagle’s Medium (Gibco^®^) supplemented with 10% fetal bovine serum (Gibco^®^), and cultured at 37 °C in an atmosphere containing 5% of CO_2_ for 24 h to reach 60% confluence. The 10 mM stock solutions of 5-HT_6_R ligands 6 and 26 in DMSO were diluted into fresh growth medium and added into the microplates at the final concentrations 0.1 μM –100 μM. Total DMSO concentration did not exceed 1% in growth media. The cytostatic drug doxorubicin (Sigma-Aldrich) at the concentration 1 μM was used as a reference. After 72 h of incubation 20 μL of CellTiter 96^®^ AQueous Non-Radioactive Cell Proliferation Assay (MTS) (Promega^®^) was added to each well and the cells were incubated under the same conditions for 2 h. The absorbance of the samples was measured using a microplate reader EnSpire (PerkinElmer, Waltham, MA, USA) at 490 nm. Statistical significance was analyzed by GraphPad Prism™ software v. 5.01 (GraphPad Software, La Jolla, CA, USA) using One-way ANOVA and Bonferroni’s Multiple Comparison Post Test.

### 4.5. Studies In Vivo

#### 4.5.1. Animals

The experiments were performed on male Wistar rats (200–220 g—behavioural test or 170–190 g—metabolic assay) obtained from an accredited animal facility at the Jagiellonian University Medical College, Poland. The animals were housed in a group of four in a controlled environment (ambient temperature 21 ± 2 °C; relative humidity 50–60%; 12 h light/dark cycles (lights on at 8:00). Standard laboratory food (LSM-B) and filtered water were freely available (behavioral tests). Animals were assigned randomly to treatment groups. All the experiments were performed by two observers unaware of the treatment applied between 9:00 and 14:00 on separate groups of animals. All animals were used only once.

The experimental protocols and procedures described in this manuscript were approved by the I Local Ethics Commission in Cracow (no 293/2015) and complied with the European Communities Council Directive of 24 November 1986 (86/609/EEC) and were in accordance with the 1996 NIH Guide for the Care and Use of Laboratory Animals.

#### 4.5.2. Drugs

Compounds were suspended in 1% Tween 80 immediately before administration in a volume of 2 mL/kg—behavioral tests or 1 mL/kg—body weight measures. Compounds were administered intraperitoneally (i.p.) 60 min before testing—behavioral tests. Control animals received vehicle (1% Tween 80) according to the same schedule.

#### 4.5.3. Behavioral Procedures in Rats

##### Forced Swim Test (FST Test)

The experiment was carried out according to the method of Porsolt et al. [41]. On the first day of an experiment, the animals were gently individually placed in Plexiglas cylinders (40 cm high, 18 cm in diameter) containing 15 cm of water maintained at 23–25 °C for 15 min. On removal from water, the rats were placed for 30 min in a Plexiglas box under 60-W bulb to dry. On the following day (24 h later), the rats were replaced in the cylinder and the total duration of immobility was recorded during the whole 5-min test period. The immobility was assigned when no additional activity was observed other than that necessary to keep the rat’s head above the water. Fresh water was used for each animal.

##### Vogel Conflict Drinking Test

The testing procedure was based on a method of Vogel et al. [42] and used the Anxiety Monitoring System “Vogel test” produced by TSE Systems (Germany). It was consisted of polycarbonate cages (dimensions 26.5 × 15 × 42 cm), equipped with a grid floor made from stainless steel bars and drinking bottles containing tap water. Experimental chambers were connected to PC software by control chassis and on electric shocks’ generator. On the first day of the experiment, the rats were adapted to the test chambers and drink water from the bottle spout for 10 min. Afterward, the rats were returned to their home cages and were given 30 min free access to water followed by a 24 h water deprivation period. The adaptation session and water deprivation protocols were repeated on the second day of the experiment. On the third day, the rats were placed again in the test chambers 60 min after compound **26** and diazepam administration and were given free access to the drinking tube. Recording data started immediately after the first lick and rats were punished with an electric shock (0.5 mA, lasting 1 s) delivered to the metal drinking tube every 20 licks. The number of licks and the number of shocks received during a 5 min experimental session were recorded automatically.

#### 4.5.4. Metabolic Assays In Vivo

##### The Effect of compound **6** or **26** on Body Weight by Non-Obese Rats Fed Palatable Diet (Model of Excessive Eating)

In order to determine the anorectic activity of **6** or **26**, the model of excessive eating was used [43,44]. Male Wistar rats (170–190 g) were housed in a pair in plastic cages in constant temperature facilities exposed to a light-dark cycle. Water and food were available ad libitum. Two groups of 6 rats were fed with diets consisting of milk chocolate with nuts, cheese, salted peanuts, and 7% condensed milk and also had access to standard feed (Labofeed B, Morawski Manufacturer Feed, Kcynia, Poland) and water ad libitum for 3 weeks. Palatable control group (palatable diet + vehicle) received vehicle (1% Tween 80, intraperitoneally), while palatable test group (palatable diet + 6 or palatable + 26) was injected (intraperitoneally) with 6 or 26 at the dose of 5 mg/kg b.w. dissolved in 1% Tween 80 (1 mL/kg). Body weights were measured daily, immediately prior to administration of drugs.

The palatable diet contained: 100 g peanuts—614 kcal; 100 mL condensed milk—131 kcal; 100 g milk chocolate—529 kcal; 100g cheese (Greek type)—270 kcal. The standard diet contained 100 g feed—280 kcal.

##### The Effect of compound **6** or **26** on Body Weight by Non-Obese Rats Fed Only with Standard Diet

Male Wistar rats (170–190 g) were housed in a pair in plastic cages in constant temperature facilities exposed to a light-dark cycle. Control group (standard diet + vehicle) received vehicle (1% Tween 80, intraperitoneally), while the test group (standard diet + 6 or standard diet + 26) was injected (intraperitoneally) with **6** or **26** at the dose of 5 mg/kg b.w. dissolved in 1% Tween 80 (1 mL/kg). Body weights were measured daily immediately prior to administration of drugs.

### 4.6. Statistical Analysis

#### 4.6.1. Lipophilicity

Statistical analysis was performed using Statistica v10 software (StatSoft, Tulsa, OK, USA). The correlation coefficients (*r*, *r*^2^), and the standard errors of the slope, interception, and estimate (S_a_, S_b_, S_e_) served as the basis for testing the linearity of regression plots.

#### 4.6.2. Studies In Vitro and In Vivo

Statistical calculations were performed using GraphPad Prism 6 software (GraphPad Software, La Jolla, CA, USA). Results are provided as arithmetic means with a standard error of the mean. The data of behavioral studies were evaluated by an analysis of variance one-way ANOVA followed by Bonferroni’s post hoc test (statistical significance set at *p* < 0.05). Statistical significance of body weight was calculated using one-way ANOVA with Sidak’s multiple comparison test post-hoc or two-way ANOVA with Sidak’s multiple comparison test post-hoc. Differences were considered statistically significant at: *p* ≤ 0.05, *p* ≤ 0.01, *p* ≤ 0.001.

## 5. Conclusions

These comprehensive studies, including: Computer-aided design, synthesis, pharmacological screening in vitro and in vivo, “drugability” experiments and an extensive docking-based SAR analysis, have provided an insight into chemical properties, ligand–receptor interactions and potential therapeutic perspectives for the new group of 1,3,5-triazine molecules. Lead structures with two topological variants of the hydantoin linker have been identified, followed by the synthesis of numerous lead modifications. As result, a new series of 18 active 5-HT_6_ agents, with submicromolar affinities confirmed in the radioligand binding assays, has been obtained (groups A and B). Computer-aided SAR analysis indicates the significance of both, linker and aromatic moiety properties, and their mutual dependences, for the 5-HT_6_R affinity of the 1,3,5-triazine derived compounds. Thus, the combination of 1-unsubstituted hydantoin (group B) with the β-naphthyl moiety at position 5 (**26**) was found as the most profitable, followed by either the corresponding α-naphthyl analogue (**25**) and the tri-substituted combination of 1-(4-chlorobenzyl)-3-((4-amino-6-(4-methylpiperazin-1-yl)-1,3,5-triazin-2-yl)methyl)-5,5-dimethylimidazolidine-2,4-dione (**6**, group A). The most active agent (**26**) displayed also antidepressant-like and anxiolytic-like activities in vivo, whereas the best members of both groups (**6** and **26**) demonstrated anti-obesity properties in animals fed palatable feed, as well as lipophilicity favorable for CNS drugs with a low risk of toxicity in vitro.

The interesting pharmacological properties together with a promising “drugability” designate this group of chemical molecules as a good starting point for further consideration in order to explore molecular mechanisms of the 5-HT_6_R–ligand interaction, and even to search of new drug candidates for the treatment of depression and obesity. In this context, 3-((4-Amino-6-(4-methylpiperazin-1-yl)-1,3,5-triazin-2-yl)methyl)-5-methyl-5-(naphthalen-2-yl)imidazolidine-2,4-dione (**26**) can be selected as a new lead structure.

## Figures and Tables

**Table 1:**
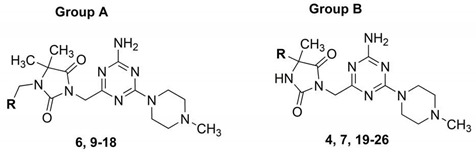
Structures and affinities for serotonin/dopamine receptors of hydantoin-triazine compounds **4**–**26**.

Cpd	Gr	R	*K*_i_ (nM) ^a^				
			5-HT_6_ [^3^H]-LSD	D_2_ [^3^H]-Raclopride	5-HT_1A_ [^3^H]-8-OH-DPAT	5-HT_2A_ [^3^H]-Ketanserin	5-HT_7_ [^3^H]-5-CT
4	B	Methyl	427	22,330	9.99 × 10^6^	nt	35,790
5	-	-	4275	5007	15,430	nt	1620
6	A	4-Chlorophenyl	127	4098	23,300	nt	3711
7	B	4-Chlorophenyl	176	31,240	28,070	nt	938
8	-	-	1058	6855	3052	nt	594
9	A	Phenyl	592	8783	42,630	1596	350
10	A	3-Chlorophenyl	271	4631	13,570	328	159
11	A	2,5-dichlorophenyl	597	2867	4155	809	279
12	A	2,4-dichlorophenyl	205	681	7693	3589	1116
13	A	4-Fluorophenyl	663	6557	48,360	3548	953
14	A	3-Fluorophenyl	447	6577	37,090	239	88
15	A	3-Bromophenyl	570	6677	12,930	1327	571
16	A	3-Methoxyphenyl	692	15,490	23,910	2036	3587
17	A	3-Methylphenyl	895	5207	16,990	817	420
18	A	2-Naphthyl	182	888	5718	1907	1525
19	B	Phenyl	726	41,040	31,620	12,150	1096
20	B	3-Chlorophenyl	418	16,780	4101	7396	791
21	B	2,5-dichlorophenyl	403	11,550	26,190	11,050	1565
22	B	2,4-dichlorophenyl	457	15,090	13,980	10,770	10,130
23	B	2,3,4-trichlorophenyl	195	4738	6028	3433	8905
24	B	4-Methylphenyl	667	23,300	22,320	24,410	564
25	B	1-Naphthyl	121	7811	7146	8080	3367
26	B	2-Naphthyl	87	4247	14,160	17,170	514
Ref.			7 ^b^	9 ^b^	20 ^c^	-	18 ^d^

^a^ Tested experimentally in the radioligand binding assay, binding affinity, *K*_i_, expressed as the average of at least two independent experiments; nt—not tested. ^b–d^ Reference ligands for GPCRs investigated, ^b^ olanzapine, ^c^ buspirone, ^d^ clozapine.

**Table 2:** The blood-brain barrier permeability parameter (QPlogBB) for compounds **4**–**26**.

Cpd	QPlogBB	Cpd	QPlogBB	Cpd	QPlogBB
**4**	−0.97	**12**	−0.33	**20**	−0.63
**5**	−0.60	**13**	−0.73	**21**	−0.46
**6**	−0.60	**14**	−0.46	**22**	−0.47
**7**	−0.89	**15**	−0.65	**23**	−0.38
**8**	−0.62	**16**	−0.92	**24**	−0.84
**9**	−0.84	**17**	−0.54	**25**	−0.98
**10**	−0.40	**18**	−0.63	**26**	−0.92
**11**	−0.37	**19**	−0.75		

## Supplementary Materials

The supplementary materials are available online.
