# Supplementary material for: Computer-Aided Studies for Novel Arylhydantoin 1,3,5-Triazine Derivatives as 5-HT6 Serotonin Receptor Ligands with Antidepressive-Like, Anxiolytic and Antiobesity Action In Vivo
Source: Molecules. 2018 Oct 3;23(10):2529. doi: 10.3390/molecules23102529 (PMC6222450; doi:10.3390/molecules23102529)
Supplement: Supplementary file 1 [file molecules-23-02529-s001.pdf]

Supplementary

Computer-aided studies for novel arylhydantoin 1,3,5-triazine derivatives as 5-HT$_6$ serotonin receptor ligands with antidepressive-like, anxiolytic and antiobesity action *in vivo*

Rafał Kurczab[1†], Wesam Ali[2,6†], Dorota Łażewska[2], Magdalena Kotańska[3], Magdalena Jastrzębska-Więsek[4], Grzegorz Satała[1], Małgorzata Więcek[2], Annamaria Lubelska[2], Gniewomir Latacz[2], Anna Partyka[4], Małgorzata Starek[5], Monika Dąbrowska[5], Anna Wesołowska[4], Claus Jacob[6], Katarzyna Kieć-Kononowicz[2], Jadwiga Handzlik[2,*]

Charcteristics of intermediates obtained before27, 29, 50-58, 63, 65 and **68-70**

Cpd	Cas number	Article
27	860787-34-4<br>3-(4-chlorobenzyl)-5,5-dimethyl-1H-imidazole-2,4(3H,5H)-dione	SAR-studies on the importance of aromatic ring topologies in search for selective 5-HT7 receptor ligands among phenylpiperazine hydantoin derivatives.<br>By Handzlik, Jadwiga et al<br>From European Journal of Medicinal Chemistry, 78, 324-339; 2014
29	179409-69-9, Aldlab Chemicals Building Blocks United States<br>methyl 2-(4,4-dimethyl-2,5-dioxoimidazolidin-1-yl)acetate	Preparation of substituted β-keto esters as intermediates for photographic yellow couplers<br>Yamakawa, Kazuyoshi; Sato, Tadahisa<br>Assignee Fuji Photo Film Co Ltd, Japan<br>1996
50	5397-13-7<br>5-(4-Chloro-phenyl)-5-methyl-imidazolidine-2,4-dion	Safari J. and Javadian L., Montmorillonite K-10 as a catalyst in the synthesis of 5, 5- disubstituted hydantoins under ultrasound irradiation, J. Chem. Sci. 125 (2013) 981– 987.
51	6843-49-8<br>5-Methyl-5-phenylhydantoin	Safari J. and Javadian L., Montmorillonite K-10 as a catalyst in the synthesis of 5, 5- disubstituted hydantoins under ultrasound irradiation, J. Chem. Sci. 125 (2013) 981– 987.
52	6946-01-6<br>5-(3-chlorophenyl)-5-methyl-2,4-imidazolidinedione	Safari J. and Javadian L., Montmorillonite K-10 as a catalyst in the synthesis of 5, 5- disubstituted hydantoins under ultrasound irradiation, J. Chem. Sci. 125 (2013) 981– 987.
53	795314-76-0<br>5-(2,5-dichlorophenyl)-5-methylimidazolidine-2,4-dione	Werbel LM, Elslager EF, Islip PJ and Closier MD, Antischistosomal effects of 5-(2,4,5-trichlorophenyl)hydantoin and related compounds, J Med Chem. 20 (1977):1569-1572.
54	64464-19-3<br>5-(2,4-dichlorophenyl)-5-methyl-2,4-imidazolidinedione	Werbel LM, Elslager EF, Islip PJ and Closier MD, Antischistosomal effects of 5-(2,4,5-trichlorophenyl)hydantoin and related compounds, J Med Chem. 20 (1977):1569-1572.
55	no	(Patent) Preparation of imidazolidinedione compounds containing substituted carbinol moiety as LXR modulators for treatment and prevention of arteriosclerosis, inflammation, diabetes, etc.<br>Matsuda, Takayuki; Okuda, Ayumu; Koura, Minoru; Yamaguchi, Yuki; Kurobuchi, Sayaka; Watanabe, Yuuichirou; Shibuya, Kimiyuki<br>Assignee: Kowa Company, Ltd., Japan<br>2008

56	23186-96-1<br>5-methyl-5-(4-methylphenyl)-2,4-imidazolidinedione	J. Linol and G. Coquerel, Influence of high energy milling on the kinetics of the polymorphic transition from the monoclinic form to the orthorhombic form of (±)5-methyl-5-(4'-methylphenyl)hydantoin. J Therm Anal Calorim 90 (2007) 367-370.
57	82752-67-8<br>5-methyl-5-(1-naphthyl)-2,4-imidazolidinedione	M. L. KeshtovA. L. RusanovN. M. Belomoina and A. K. Mikitaev, Improved synthesis of bis[*p*-(phenylethynyl)phenyl]hetarylenes, Russ Chem Bull. 46 (1997) 1794-1796
58	78772-74-4<br>5-Methyl-5-(2-naphthyl)-2,4-imidazolidinedione	M. L. KeshtovA. L. RusanovN. M. Belomoina and A. K. Mikitaev, Improved synthesis of bis[*p*-(phenylethynyl)phenyl]hetarylenes, Russ Chem Bull. 46 (1997) 1794-1796
63	1372008-89-3, Aurora Building Blocks, United States<br>Methyl 2-(4-(2,4-dichlorophenyl)-4-methyl-2,5-dioxoimidazolidin-1-yl)acetate	Aurora Building Blocks
65	1371767-82-6, Aurora Building Blocks, United States<br>Methyl 2-(4-methyl-2,5-dioxo-4-p-tolylimidazolidin-1-yl)acetate	Aurora Building Blocks
68	104-88-1,<br>4-Chlorobenzaldehyde	sigma
69	88372-92-3 china<br>1,2-bis(4-chlorophenyl)ethane-1,2-dione	88372-92-3 China
70	23186-92-7, Atomax Chemicals Product List<br>China<br>5,5-bis(4-chlorophenyl)imidazolidine-2,4-dione	Electrochemical characterization of phenytoin and its derivatives on bare gold electrode.<br>Trisovic, Nemanja P.; Bozic, Bojan Dj.; Lovic, Jelena D.; Vitnik, Vesna D.; Vitnik, Zeljko J.; Petrovic, Slobodan D.; Ivic, Milka L. Avramov.<br>Electrochimica Acta<br>Volume 161<br>Pages 378-387<br>Journal; Online Computer File. 2015

^{1}H-NMR, ^{13}C-N-NMR

for final products 5-27

This report was created by ACD/NMR Processor Academic Edition. For more information go to www.acdlabs.com/nmrproc/

1H-NMR

2017-10-10 19:24:29

Acquisition Time (sec)	1.7064	***Date***	Oct 10 2017	***Date Stamp***	Oct 10 2017		
File Name	C:\Users\Dorota\Desktop\ANALIZY\NMR\2017\17-10-10_DJ24_dlaz\17-10-10_DJ24_dlaz_PROTON_01.fid\fid					***Frequency (MHz)***	300.08
Nucleus	1H	***Number of Transients***	32	***Original Points Count***	8192	***Points Count***	8192
Pulse Sequence	s2pul	***Receiver Gain***	34.00	***Solvent***	DMSO-d6	***Spectrum Offset (Hz)***	1800.4814
Spectrum Type	STANDARD	***Sweep Width (Hz)***	4800.77	***Temperature (degree C)***	AMBIENT TEMPERATURE		

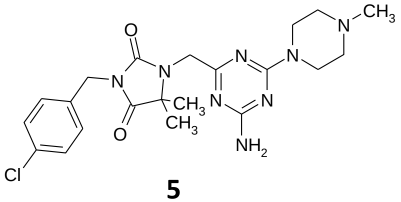

5

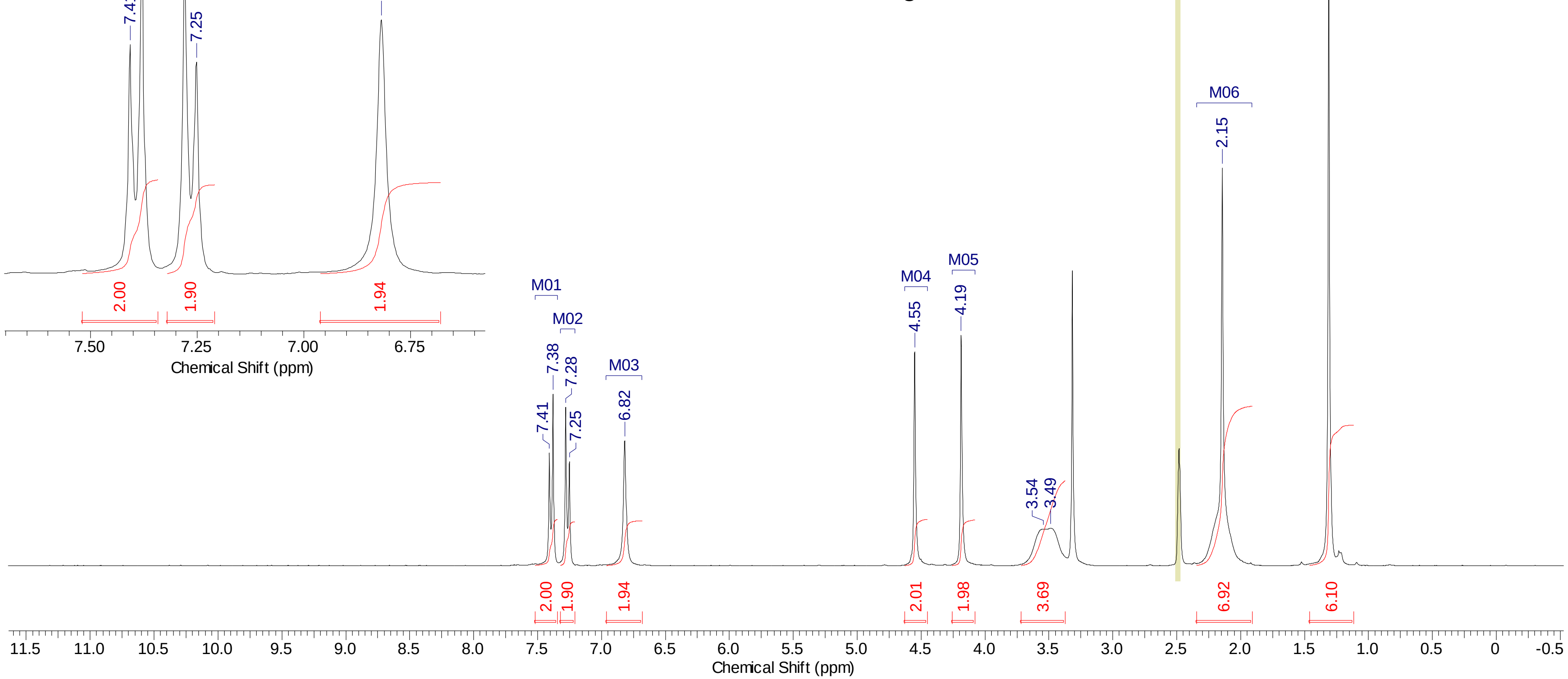

This report was created by ACD/NMR Processor Academic Edition. For more information go to www.acdlabs.com/nmrproc/

C13-NMR

**Acquisition Time (sec)**	0.8684	**Date**	Oct 17 2017	**Date Stamp**	Oct 17 2017		2017-10-18 11:39:50
**File Name**	C:\Users\Dorota\Desktop\ANALIZY\NMR\2017\17-10-17_DJ24_dlaz\17-10-17_DJ24_dlaz_CARBON_01.fid\fid						
**Frequency (MHz)**	75.46	**Nucleus**	13C	**Number of Transients**	3000	**Original Points Count**	16384
**Points Count**	16384	**Pulse Sequence**	s2pul	**Receiver Gain**	34.00	**Solvent**	DMSO-d6
**Spectrum Offset (Hz)**	8300.0879	**Spectrum Type**	STANDARD	**Sweep Width (Hz)**	18867.92	**Temperature (degree C)**	23.000

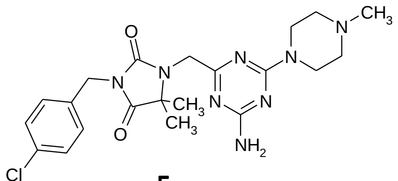

5

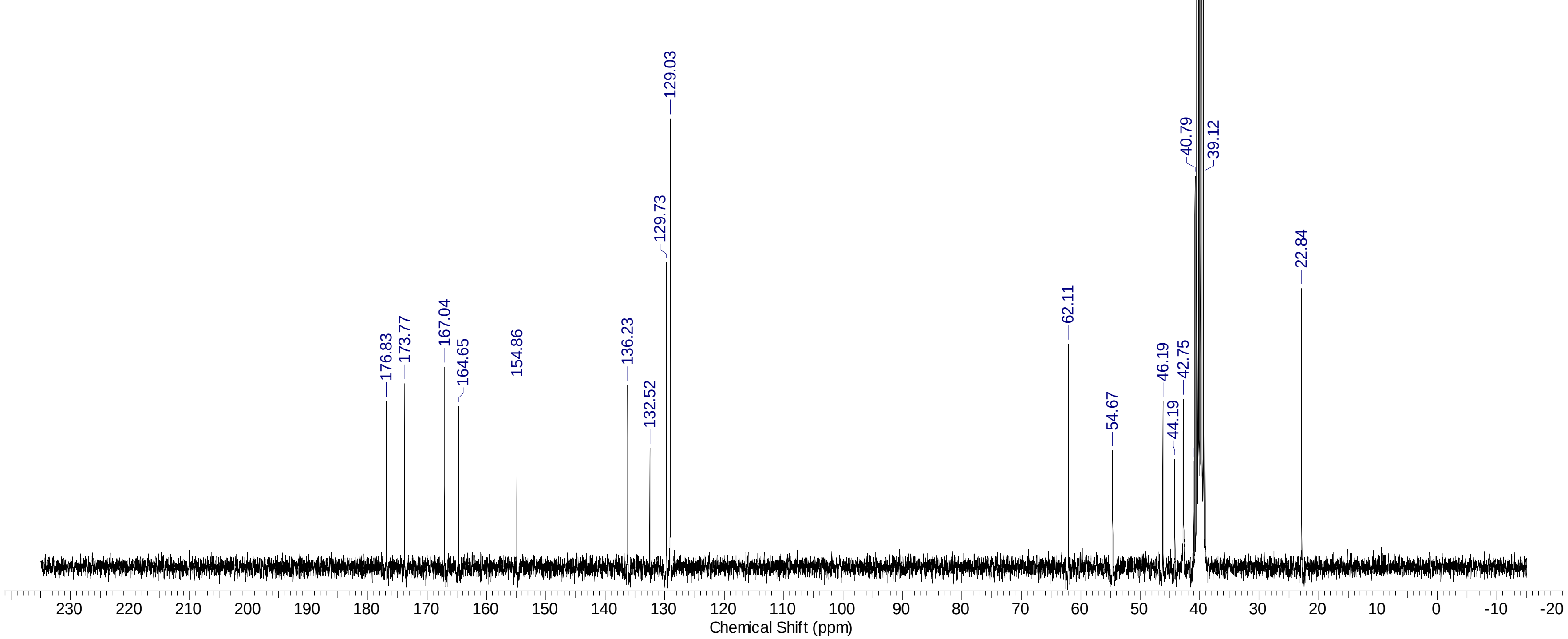

This report was created by ACD/NMR Processor Academic Edition. For more information go to www.acdlabs.com/nmrproc/

1H-NMR

2017-10-10 18:46:57

Acquisition Time (sec)	1.7064	***Date***	Oct 10 2017	***Date Stamp***	Oct 10 2017		
File Name	C:\Users\Dorota\Desktop\ANALIZY\NMR\2017\17-10-10_DJ18_dlaz\17-10-10_DJ18_dlaz_PROTON_01.fid\fid					***Frequency (MHz)***	300.08
Nucleus	1H	***Number of Transients***	32	***Original Points Count***	8192	***Points Count***	8192
Pulse Sequence	s2pul	***Receiver Gain***	38.00	***Solvent***	DMSO-d6	***Spectrum Offset (Hz)***	1800.4814
Spectrum Type	STANDARD	***Sweep Width (Hz)***	4800.77	***Temperature (degree C)***	AMBIENT TEMPERATURE		

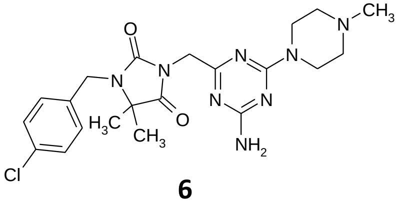

6

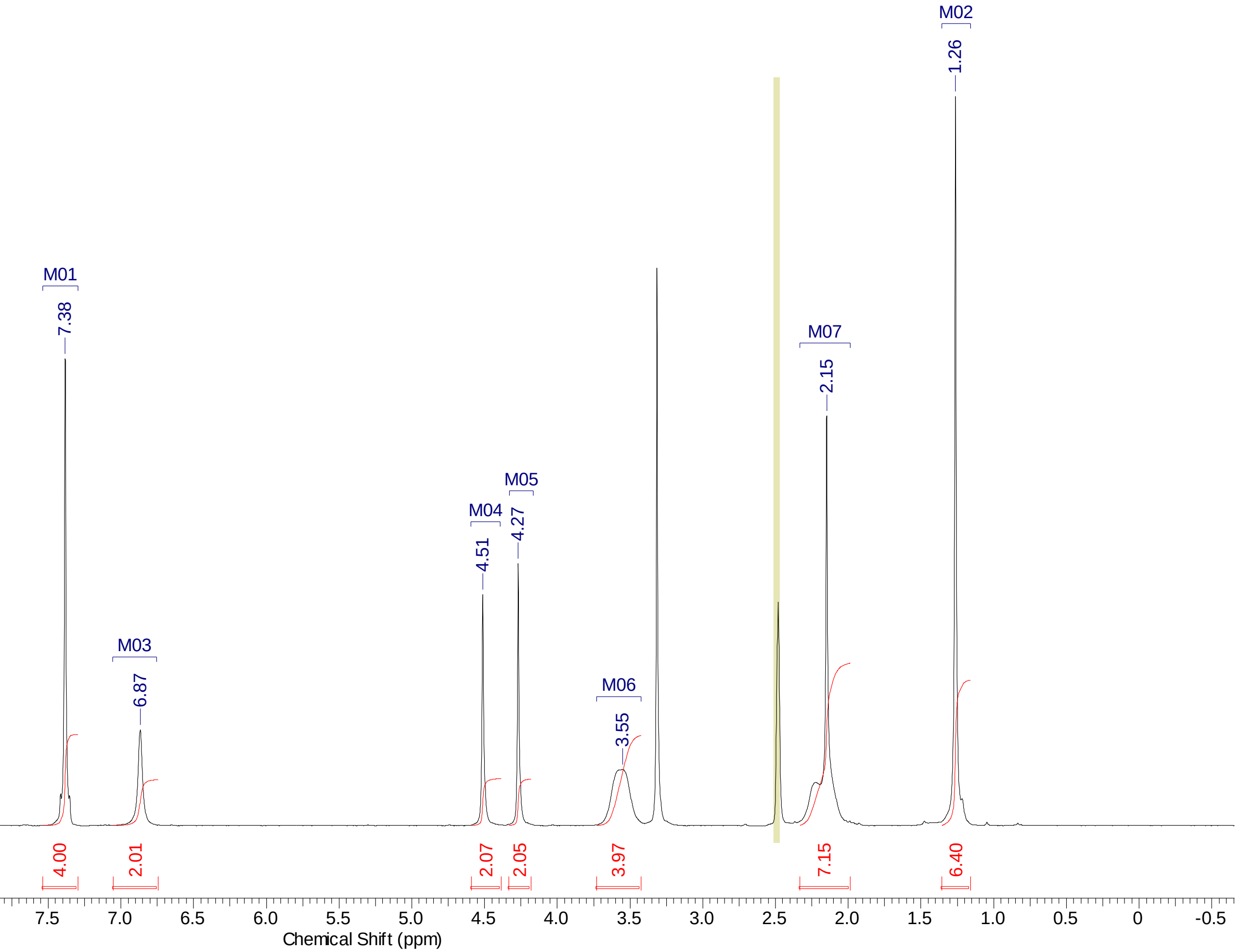

This report was created by ACD/NMR Processor Academic Edition. For more information go to www.acdlabs.com/nmrproc/

1H-NMR

2017-10-10 13:03:40

Acquisition Time (sec)	1.7064	***Date***	Oct 10 2017	***Date Stamp***	Oct 10 2017		
File Name	C:\Users\Dorota\Desktop\ANALIZY\NMR\2017\17-10-10_DJ2_dlaz\17-10-10_DJ2_dlaz_PROTON_01.fid\fid					***Frequency (MHz)***	300.08
Nucleus	1H	***Number of Transients***	32	***Original Points Count***	8192	***Points Count***	8192
Pulse Sequence	s2pul	***Receiver Gain***	38.00	***Solvent***	DMSO-d6	***Spectrum Offset (Hz)***	1800.4814
Spectrum Type	STANDARD	***Sweep Width (Hz)***	4800.77	***Temperature (degree C)***	AMBIENT TEMPERATURE		

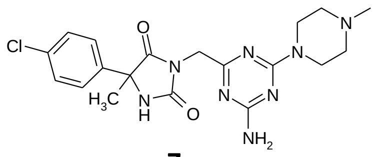

7

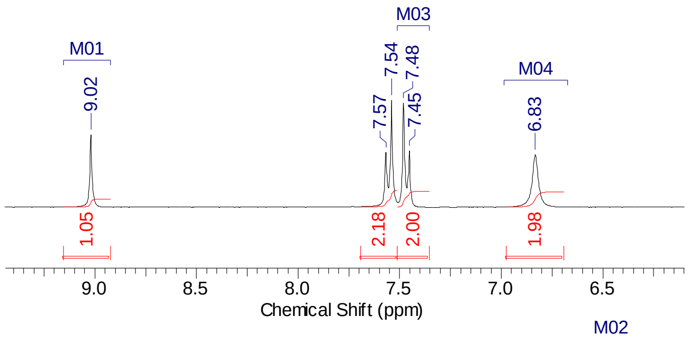

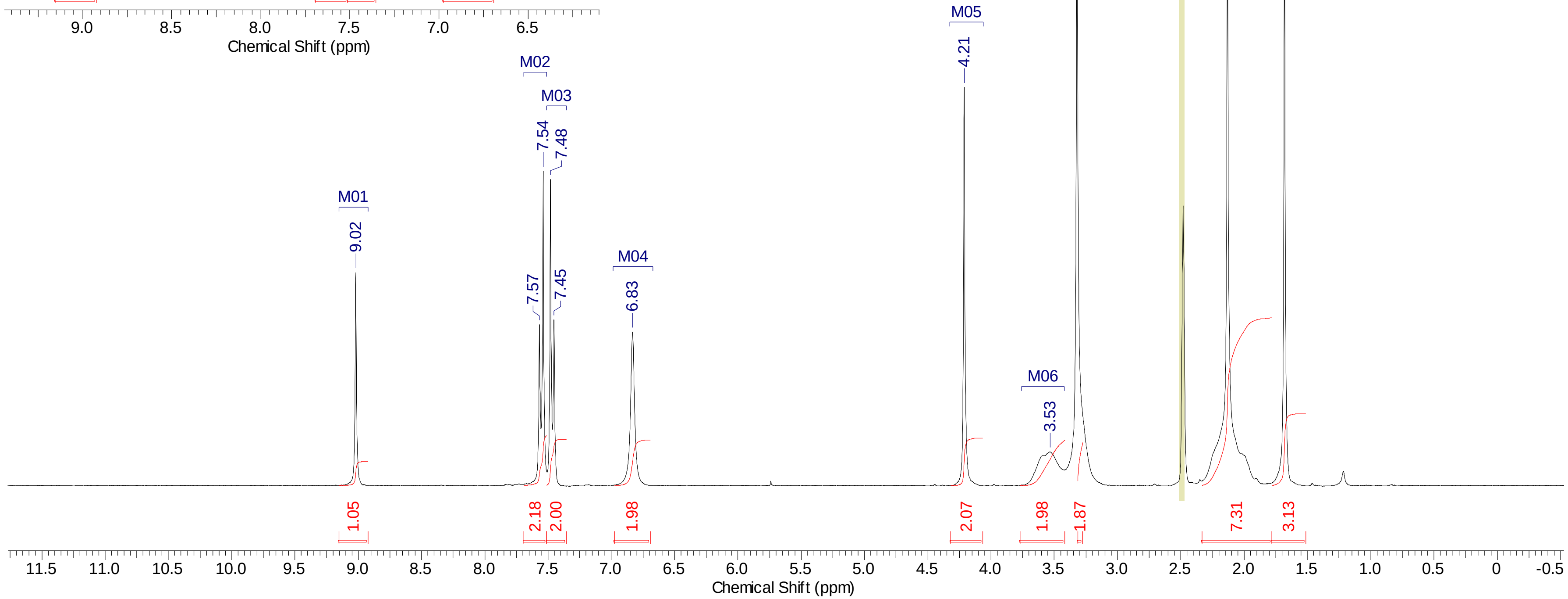

This report was created by ACD/NMR Processor Academic Edition. For more information go to www.acdlabs.com/nmrproc/

C13-NMR

2017-10-18 11:19:51

Acquisition Time (sec)	0.8684	***Date***	Oct 17 2017	***Date Stamp***	Oct 17 2017		
File Name	C:\Users\Dorota\Desktop\ANALIZY\NMR\2017\17-10-17_DJ2_dlaz\17-10-17_DJ2_dlaz_CARBON_01.fid\fid					***Frequency (MHz)***	75.46
Nucleus	13C	***Number of Transients***	1536	***Original Points Count***	16384	***Points Count***	16384
Pulse Sequence	s2pul	***Receiver Gain***	34.00	***Solvent***	DMSO-d6	***Spectrum Offset (Hz)***	8300.0879
Spectrum Type	STANDARD	***Sweep Width (Hz)***	18867.92	***Temperature (degree C)***	23.000		

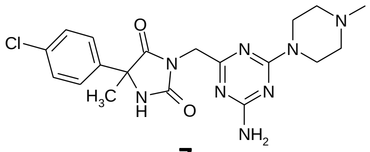

7

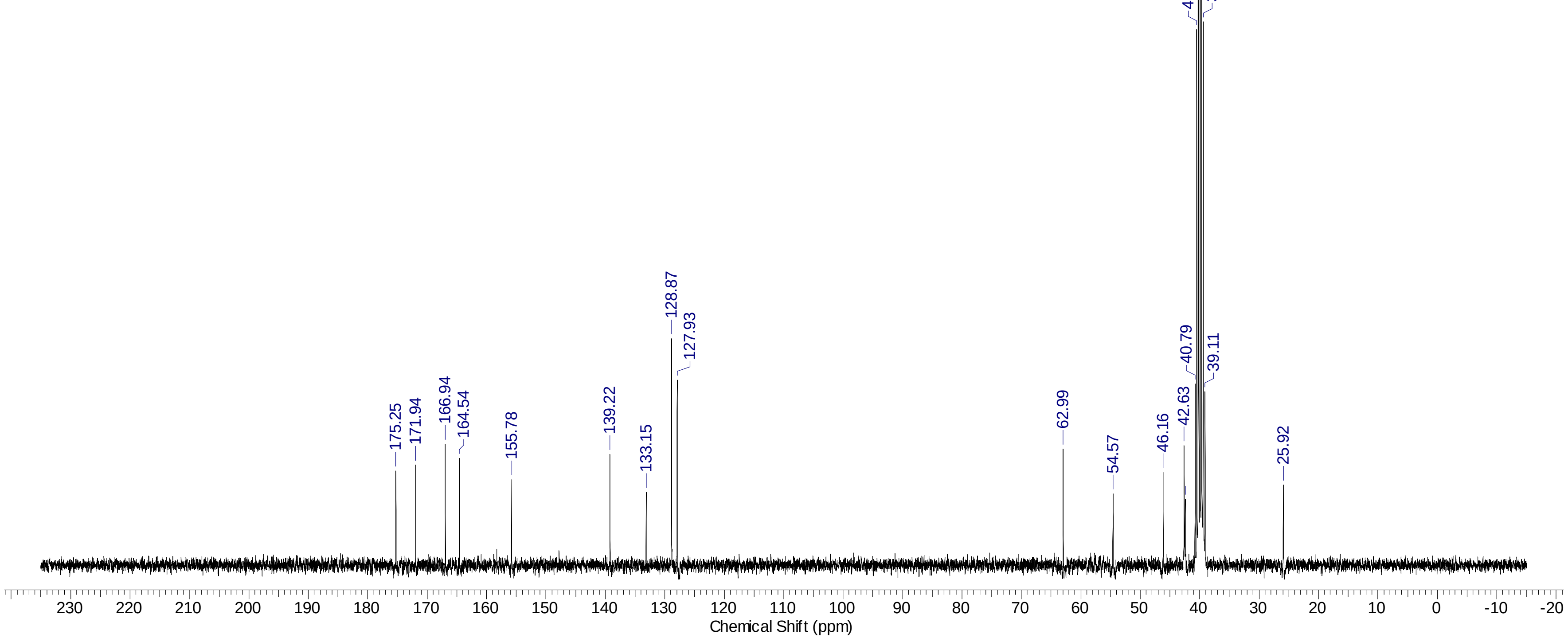

This report was created by ACD/NMR Processor Academic Edition. For more information go to www.acdlabs.com/nmrproc/

1H-NMR

2017-10-10 13:19:36

Acquisition Time (sec)	1.7064	***Date***	Oct 10 2017	***Date Stamp***	Oct 10 2017		
File Name	C:\Users\Dorota\Desktop\ANALIZY\NMR\2017\17-10-10_DJ6_dlaz\17-10-10_DJ6_dlaz_PROTON_01.fid\fid					***Frequency (MHz)***	300.08
Nucleus	1H	***Number of Transients***	32	***Original Points Count***	8192	***Points Count***	8192
Pulse Sequence	s2pul	***Receiver Gain***	38.00	***Solvent***	DMSO-d6	***Spectrum Offset (Hz)***	1800.4814
Spectrum Type	STANDARD	***Sweep Width (Hz)***	4800.77	***Temperature (degree C)***	AMBIENT TEMPERATURE		

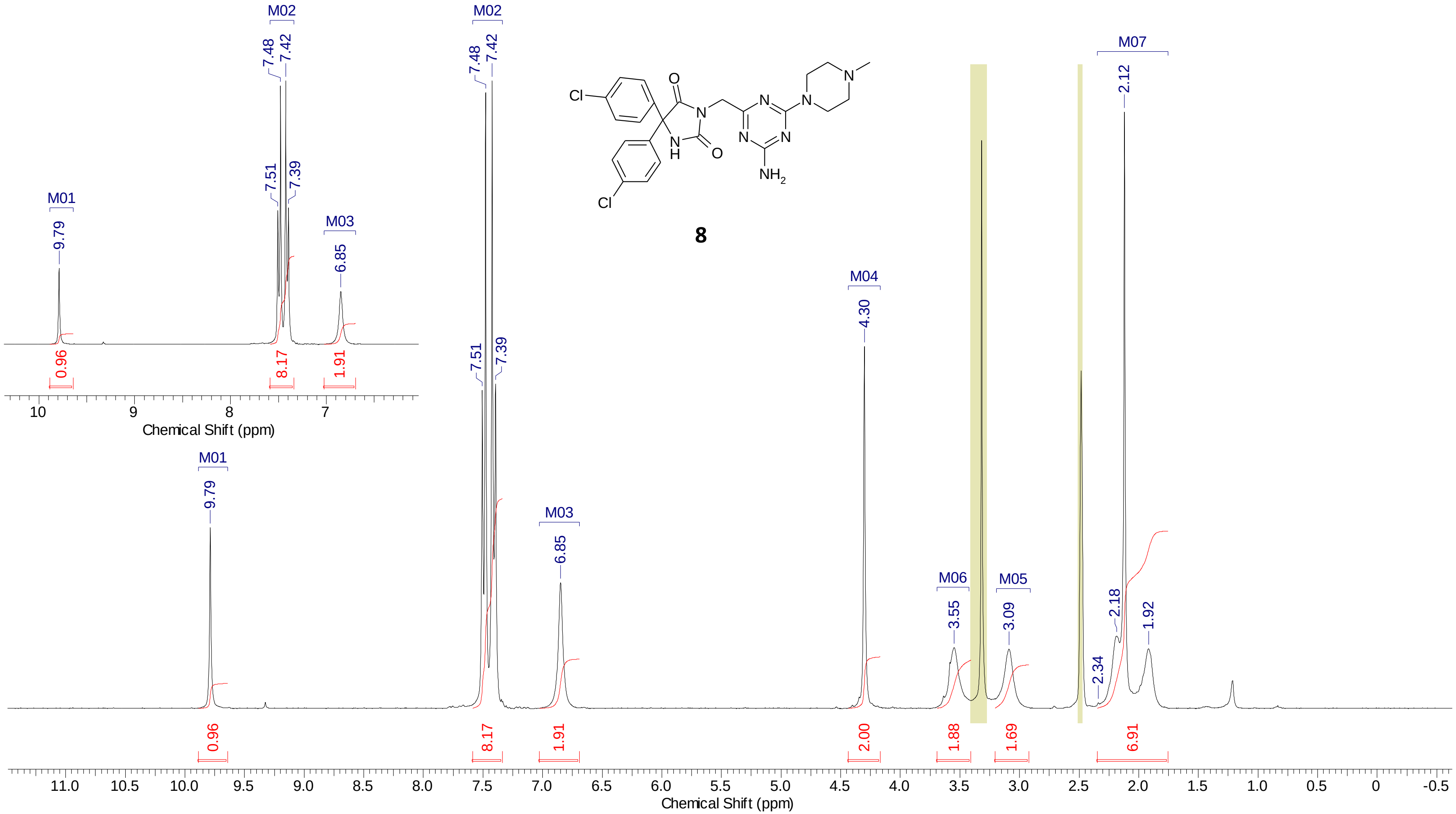

This report was created by ACD/NMR Processor Academic Edition. For more information go to www.acdlabs.com/nmrproc/

C13-NMR

2017-10-18 11:33:03

Acquisition Time (sec)	0.8684	***Date***	Oct 16 2017	***Date Stamp***	Oct 16 2017		
File Name	C:\Users\Dorota\Desktop\ANALIZY\NMR\2017\17-10-16_DJ6_dlaz\17-10-16_DJ6_dlaz_CARBON_01.fid\fid					***Frequency (MHz)***	75.46
Nucleus	13C	***Number of Transients***	2224	***Original Points Count***	16384	***Points Count***	16384
Pulse Sequence	s2pul	***Receiver Gain***	34.00	***Solvent***	DMSO-d6	***Spectrum Offset (Hz)***	8300.0879
Spectrum Type	STANDARD	***Sweep Width (Hz)***	18867.92	***Temperature (degree C)***	23.000		

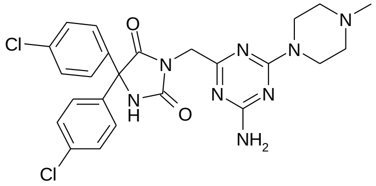

8

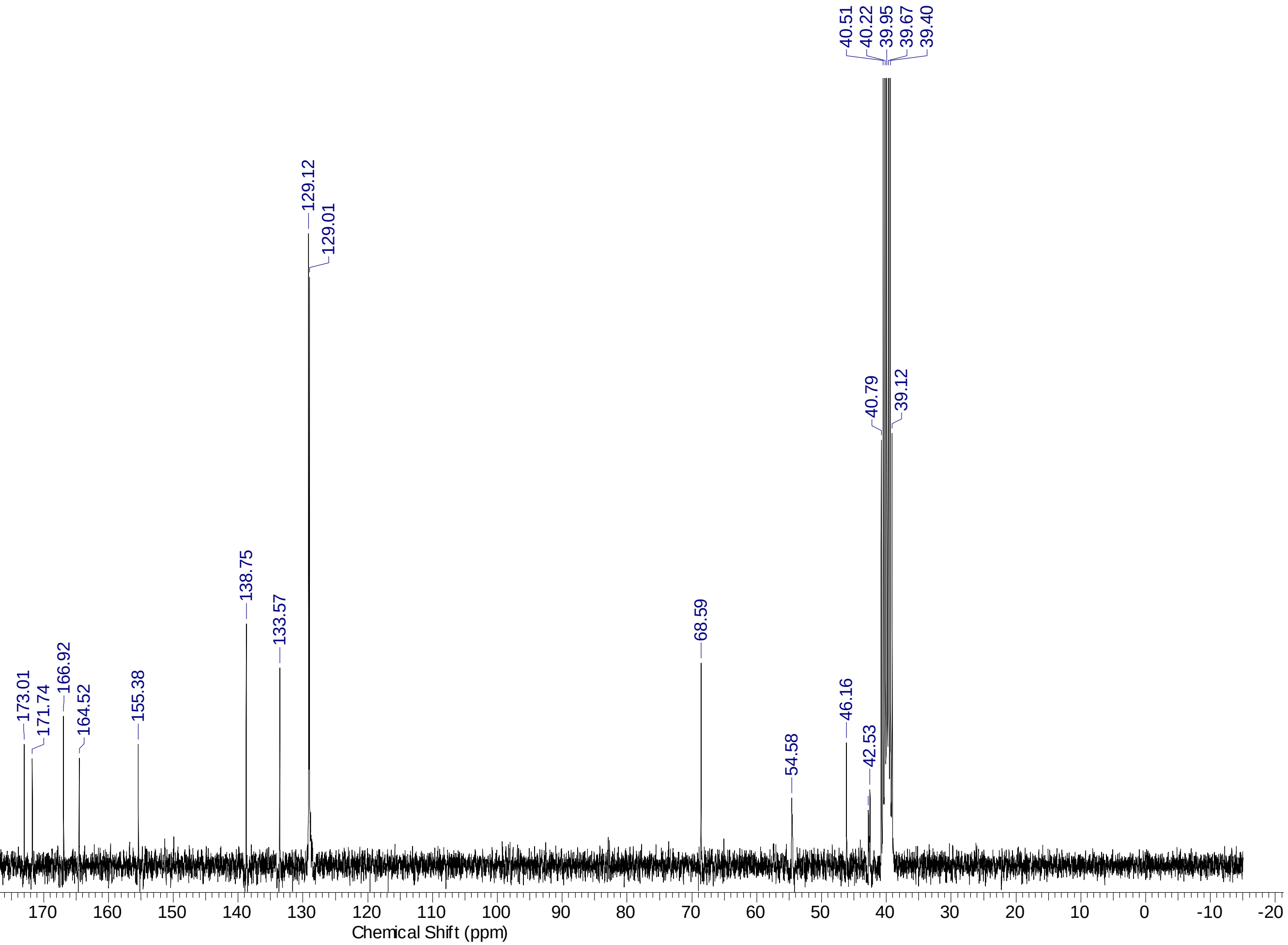

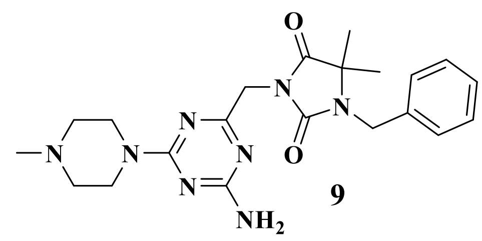

9

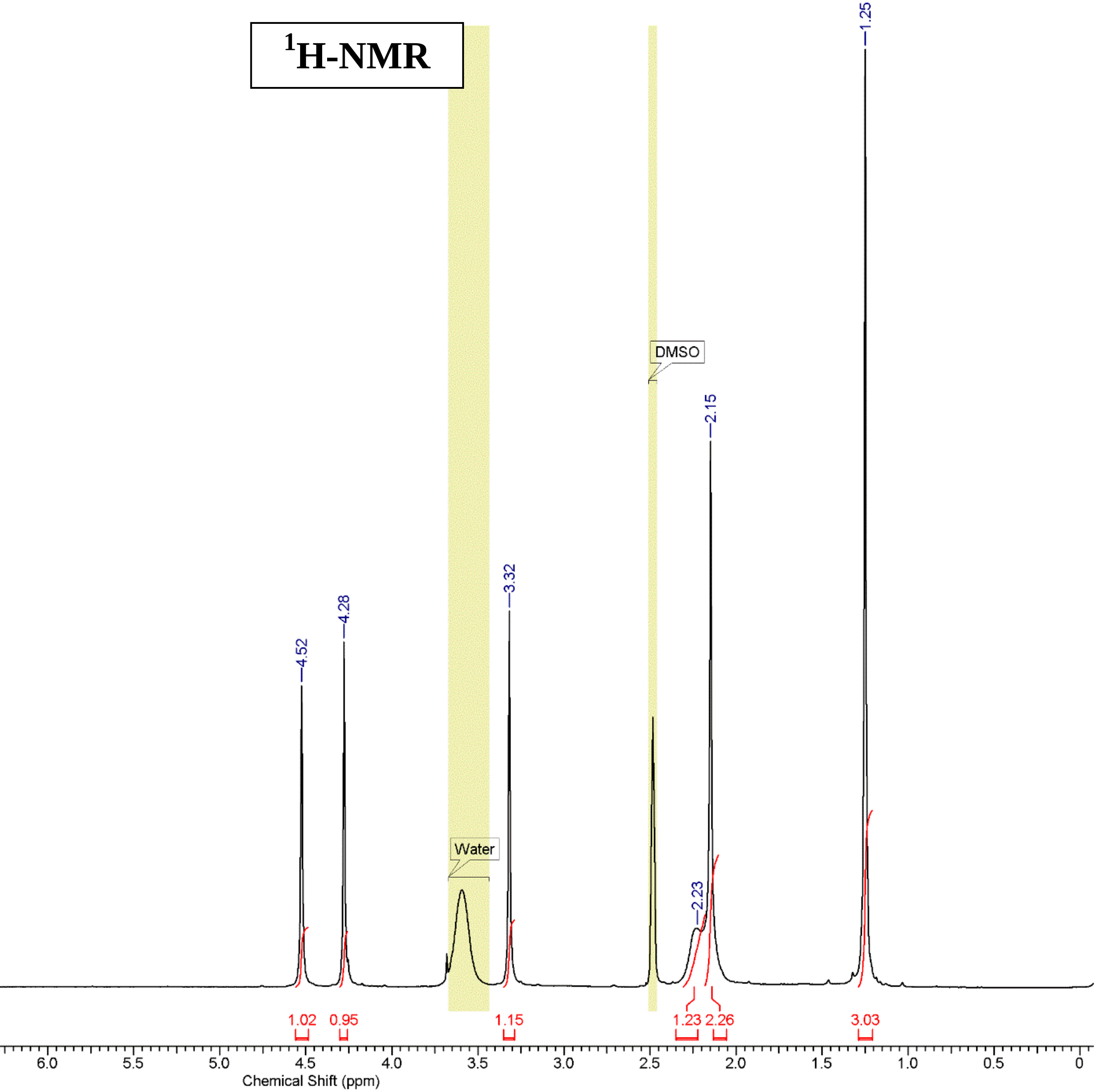

¹H-NMR
Water
DMSO
7.36
7.33
7.31
7.28
7.27
7.25
6.87
4.52
4.28
3.32
2.23
2.15
1.25
1.91
0.67
0.96
1.02
0.95
1.15
1.23
2.26
3.03
Chemical Shift (ppm)

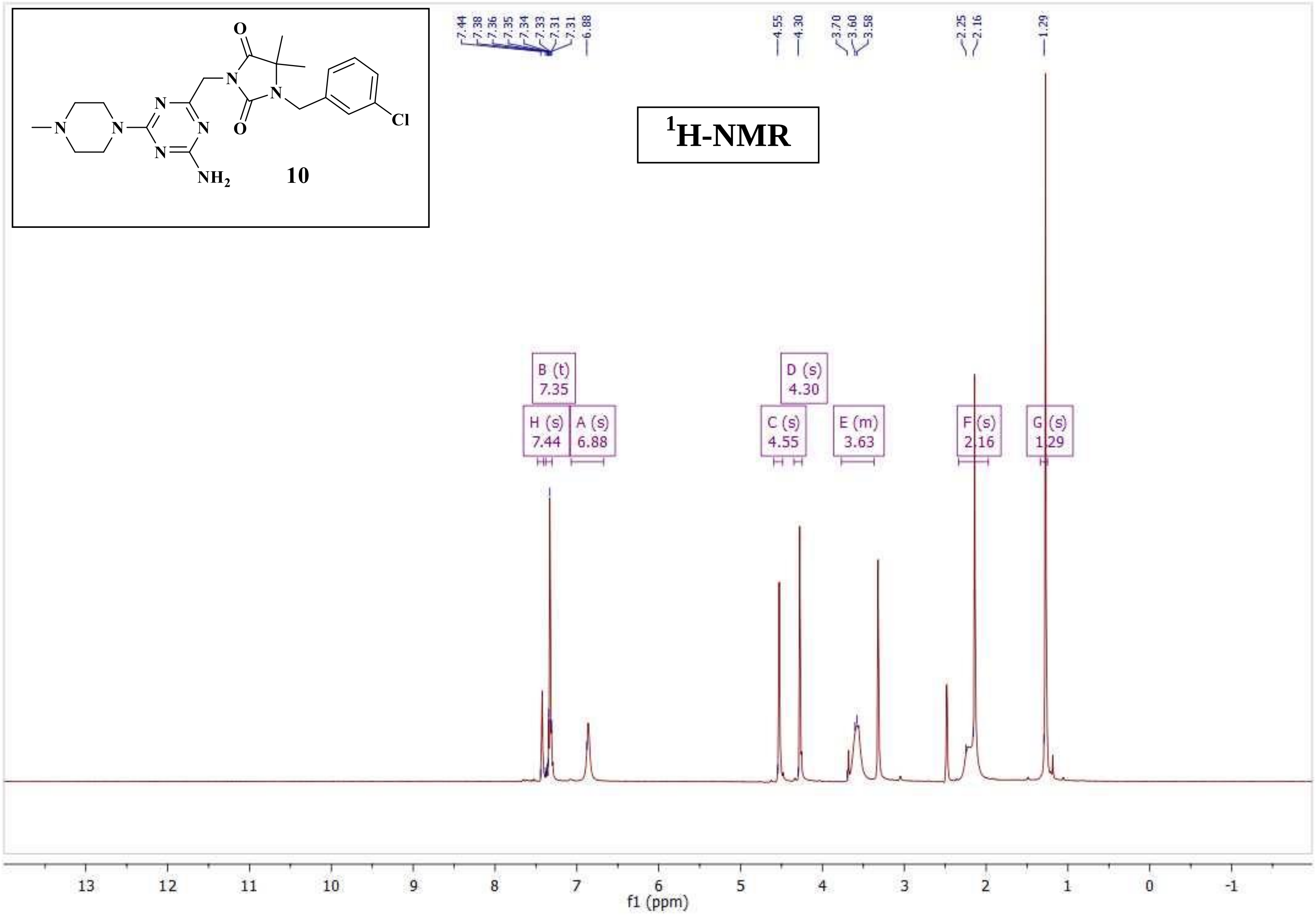

10
1H-NMR
7.44
7.38
7.36
7.35
7.34
7.33
7.31
7.31
6.88
4.55
4.30
3.70
3.60
3.58
2.25
2.16
1.29
B (t) 7.35
H (s) 7.44
A (s) 6.88
D (s) 4.30
C (s) 4.55
E (m) 3.63
F (s) 2.16
G (s) 1.29
13
12
11
10
9
8
7
6
5
4
3
2
1
0
-1
f1 (ppm)

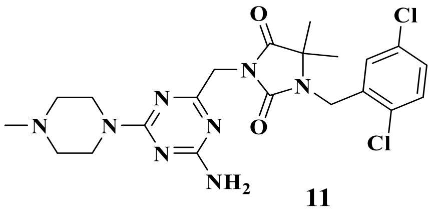
O
Cl
N
N
N
N
N
N
O
Cl
NH2

11

^{1}H-NMR

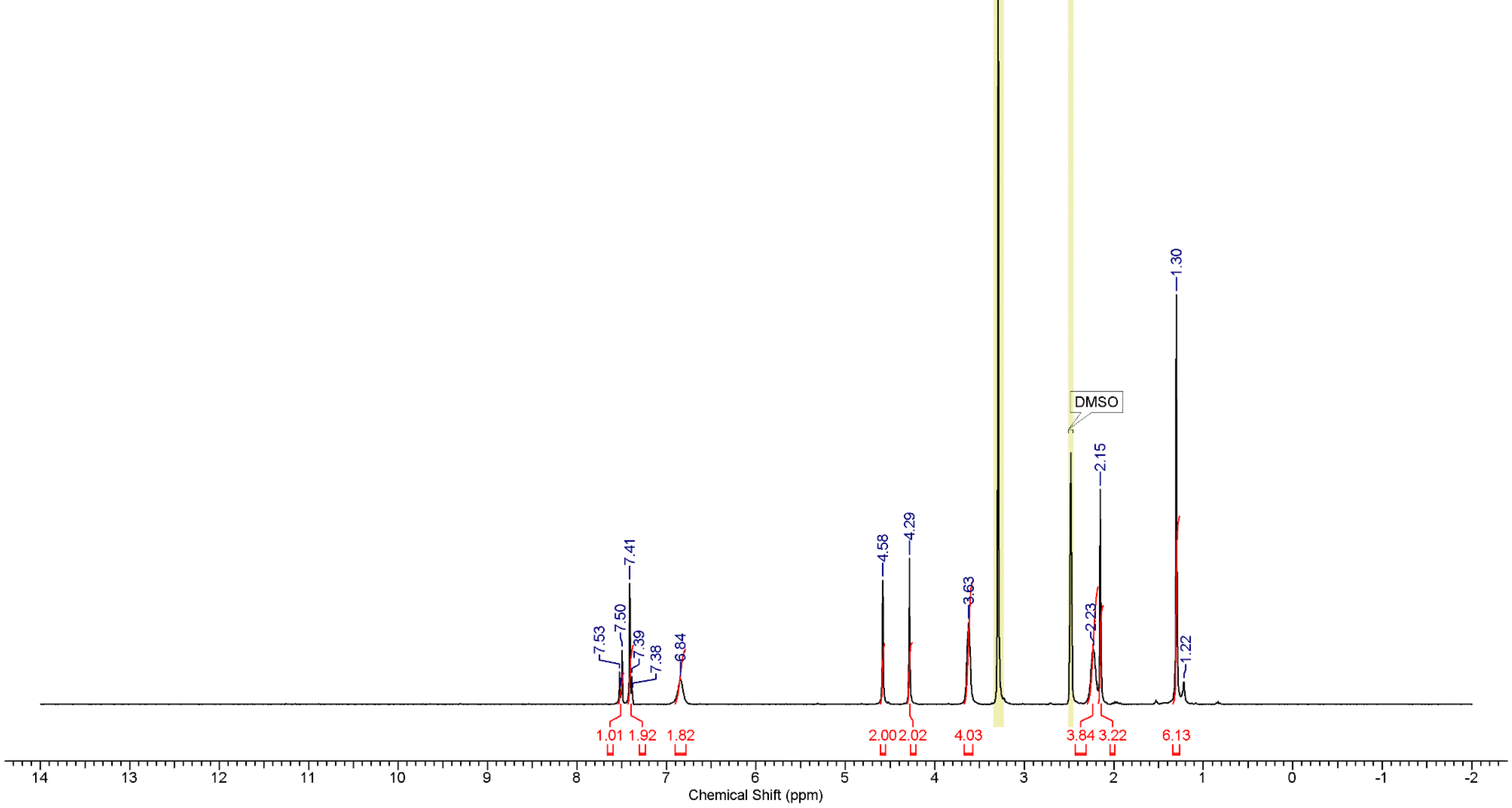
Water
DMSO
7.53
7.50
7.41
7.39
7.38
6.84
4.58
4.29
3.63
2.23
2.15
1.30
1.22
1.01
1.92
1.82
2.00
2.02
4.03
3.84
3.22
6.13
14
13
12
11
10
9
8
7
6
5
4
3
2
1
0
-1
-2
Chemical Shift (ppm)

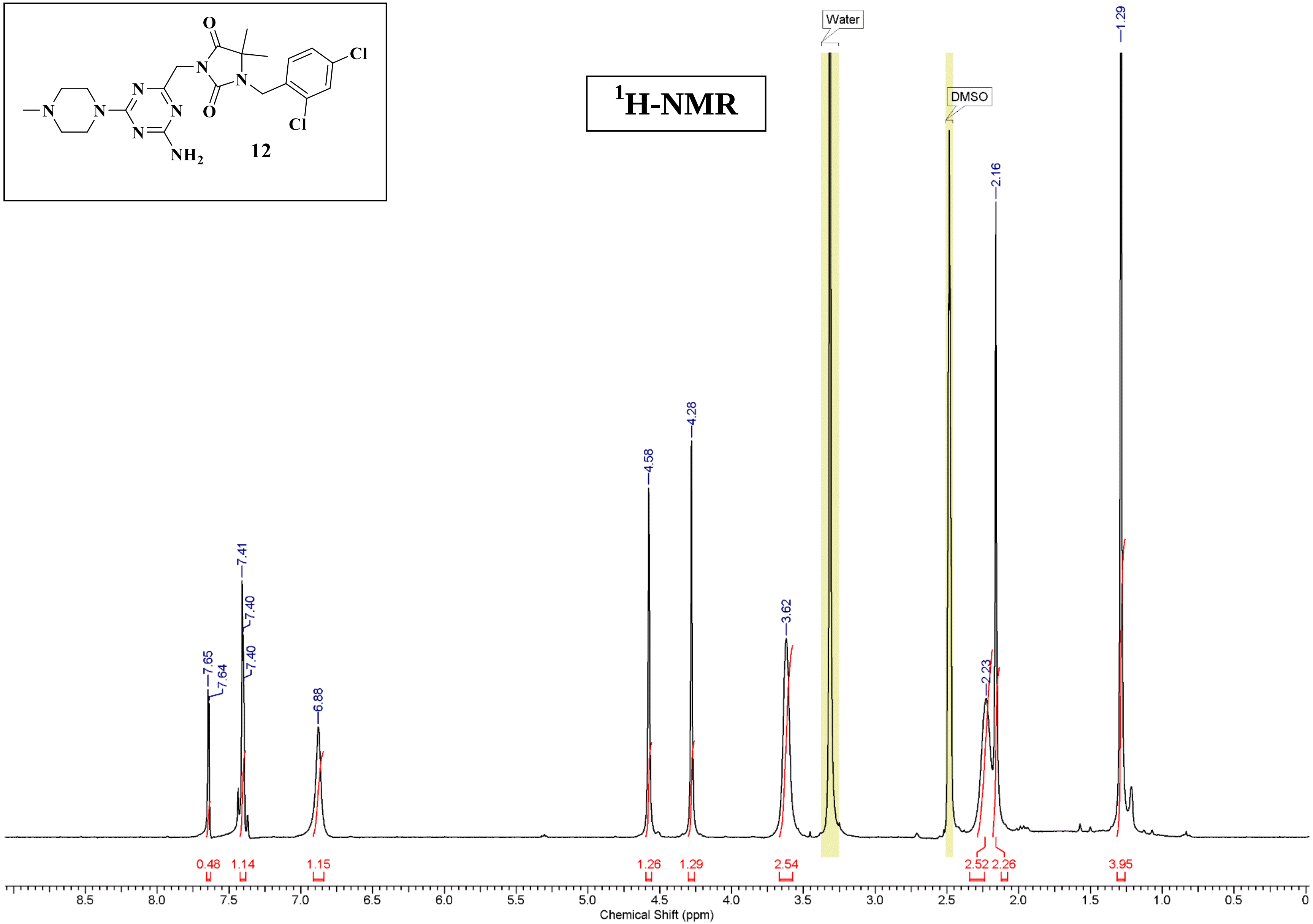

O
O
N
N
N
N
N
N
N
Cl
Cl
NH2
12
1H-NMR
Water
DMSO
7.65
7.64
7.41
7.40
7.40
6.88
4.58
4.28
3.62
2.23
2.16
1.29
0.48
1.14
1.15
1.26
1.29
2.54
2.52
2.26
3.95
8.5
8.0
7.5
7.0
6.5
6.0
5.5
5.0
4.5
4.0
3.5
3.0
2.5
2.0
1.5
1.0
0.5
0
Chemical Shift (ppm)

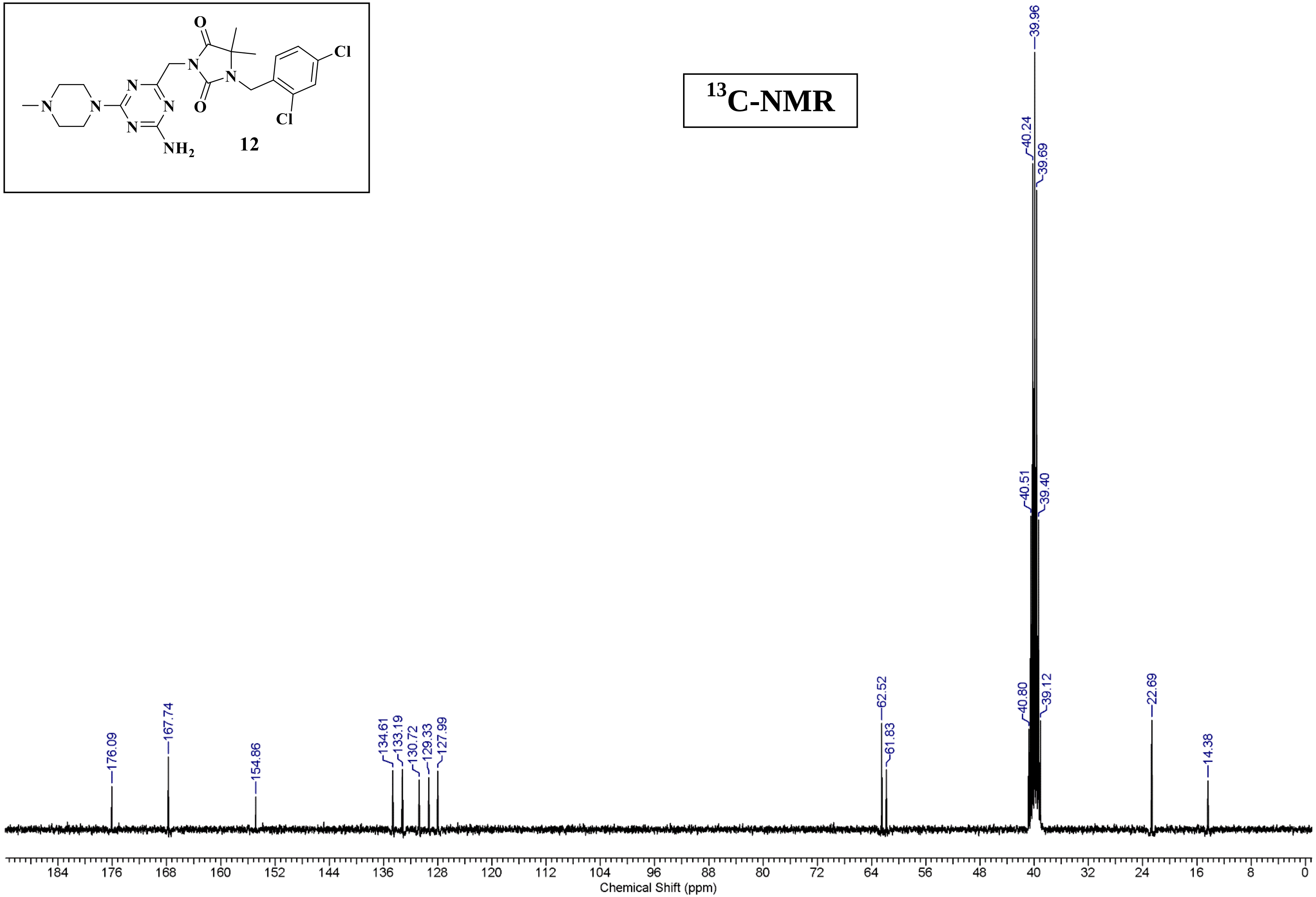
12
13C-NMR
176.09
167.74
154.86
134.61
133.19
130.72
129.33
127.99
62.52
61.83
40.80
40.51
40.24
39.96
39.69
39.40
39.12
22.69
14.38
Chemical Shift (ppm)

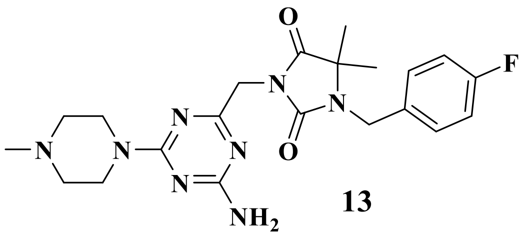
O
N
N
F
N
N
N
N
O
N
NH2

13

^{1}H-NMR

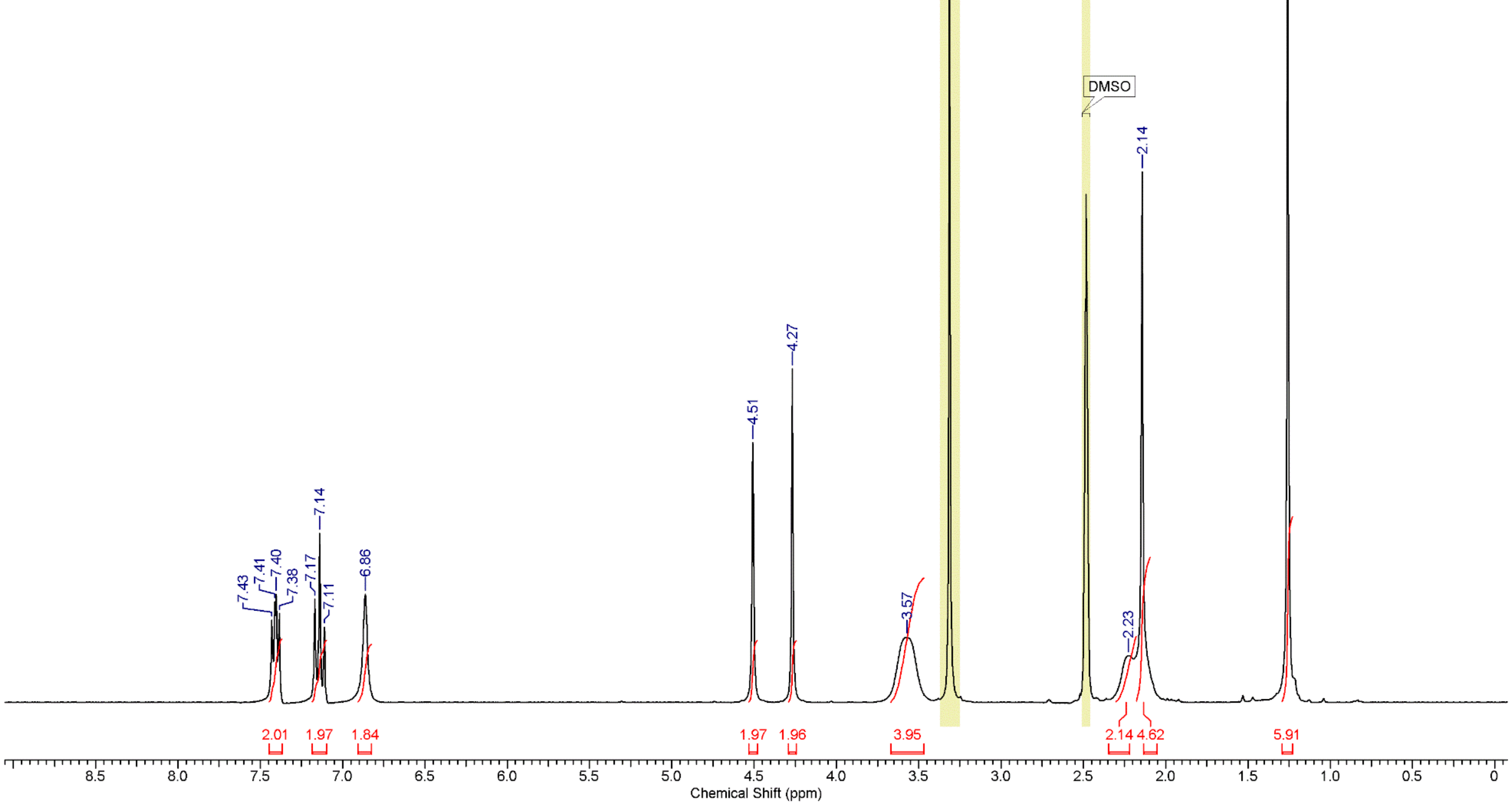
Water
DMSO
7.43
7.41
7.40
7.38
7.17
7.14
7.11
6.86
4.51
4.27
3.57
2.23
2.14
1.26
2.01
1.97
1.84
1.97
1.96
3.95
2.14
4.62
5.91
8.5
8.0
7.5
7.0
6.5
6.0
5.5
5.0
4.5
4.0
3.5
3.0
2.5
2.0
1.5
1.0
0.5
0
Chemical Shift (ppm)

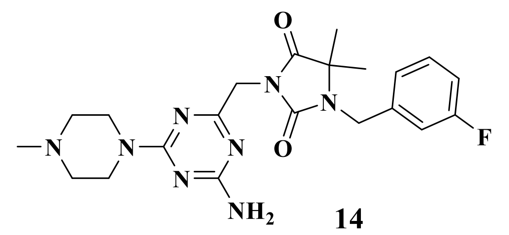

14

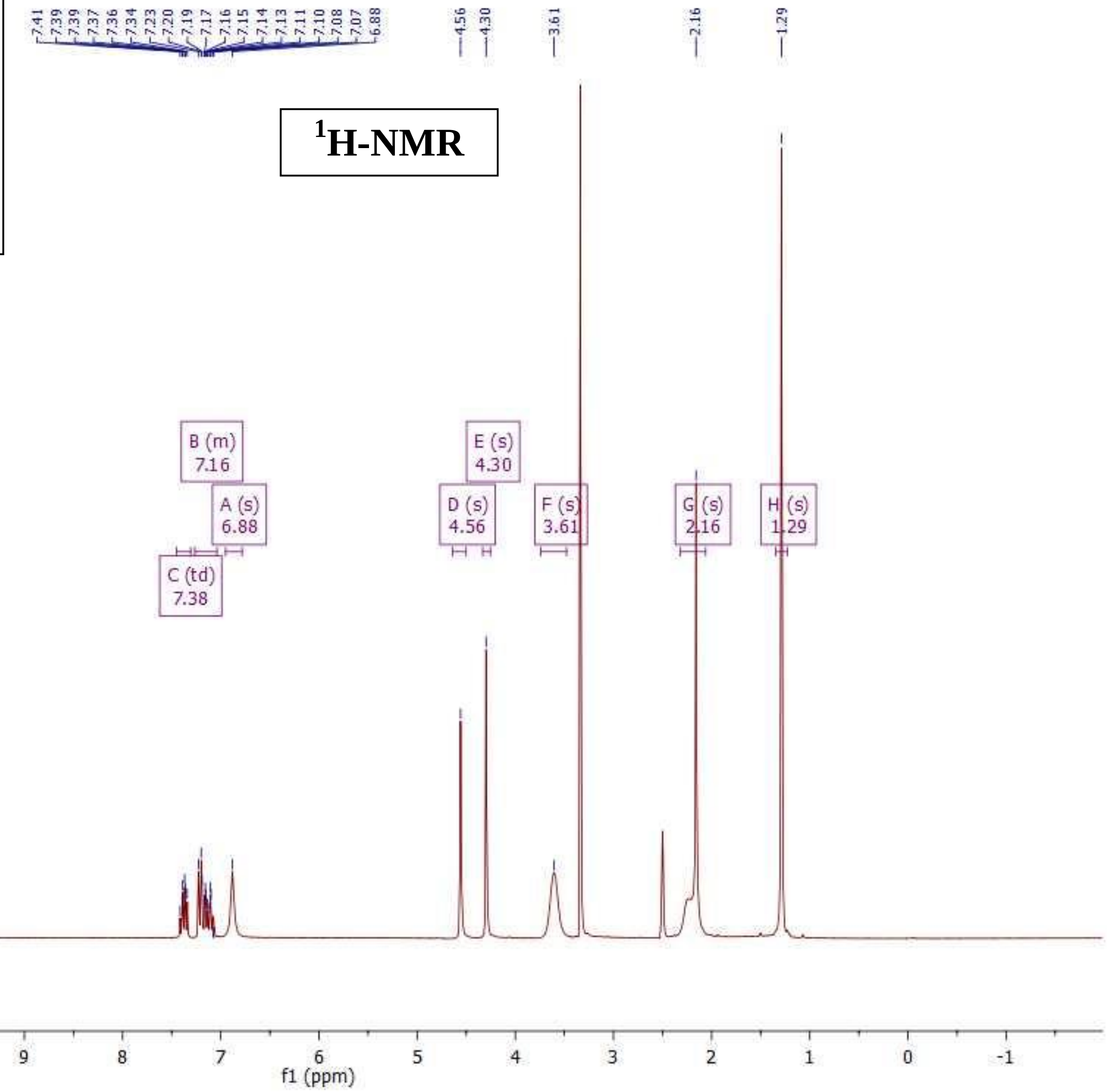

7.41
7.39
7.39
7.37
7.36
7.34
7.23
7.20
7.19
7.17
7.16
7.15
7.14
7.13
7.11
7.10
7.08
7.07
6.88
4.56
4.30
3.61
2.16
1.29
¹H-NMR
B (m) 7.16
A (s) 6.88
C (td) 7.38
E (s) 4.30
D (s) 4.56
F (s) 3.61
G (s) 2.16
H (s) 1.29
13
12
11
10
9
8
7
6
5
4
3
2
1
0
-1
f1 (ppm)

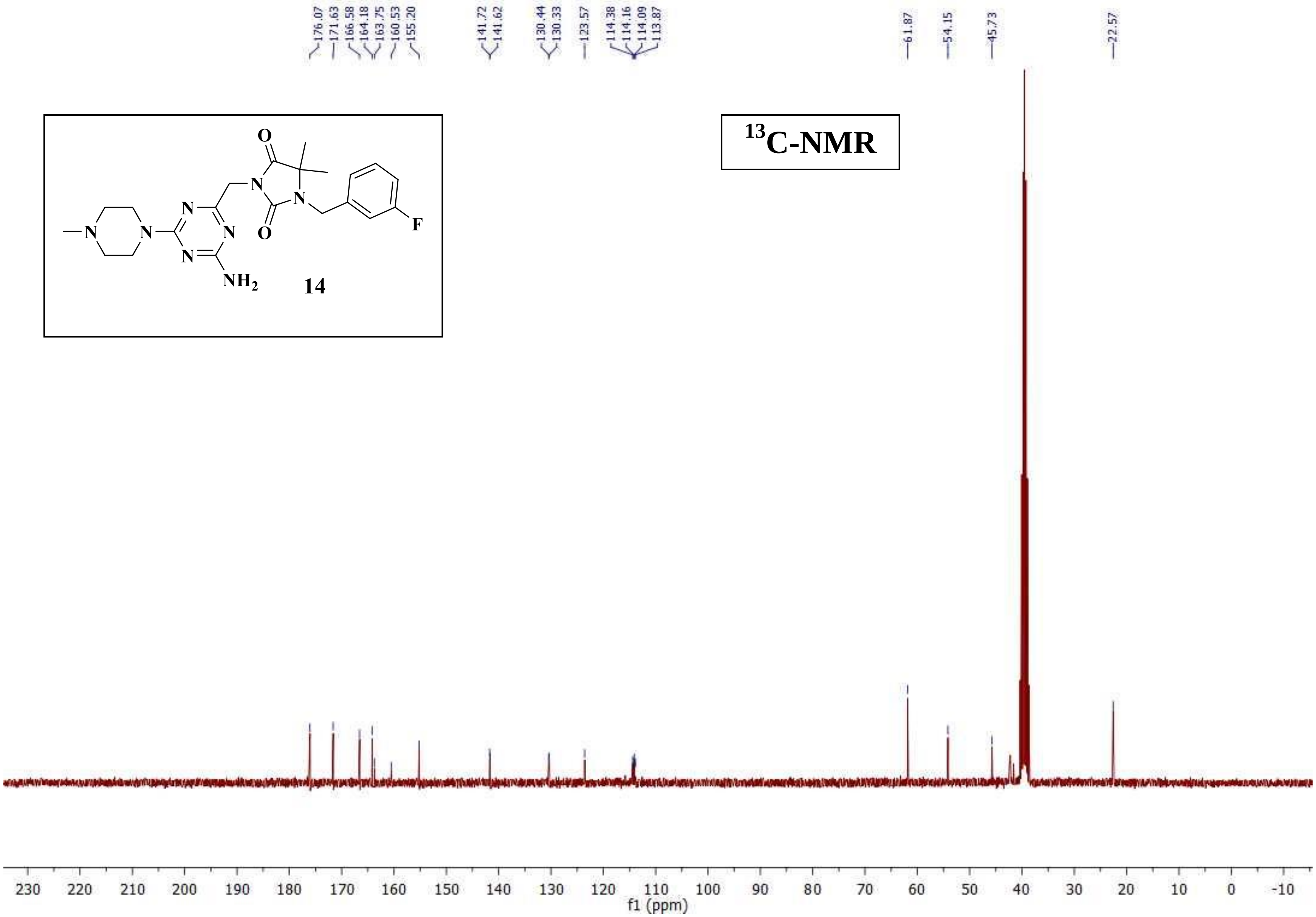
13C-NMR
14
176.07 171.63 166.58 164.18 163.75 160.53 155.20 141.72 141.62 130.44 130.33 123.57 114.38 114.16 114.09 113.87 61.87 54.15 45.73 22.57
f1 (ppm)

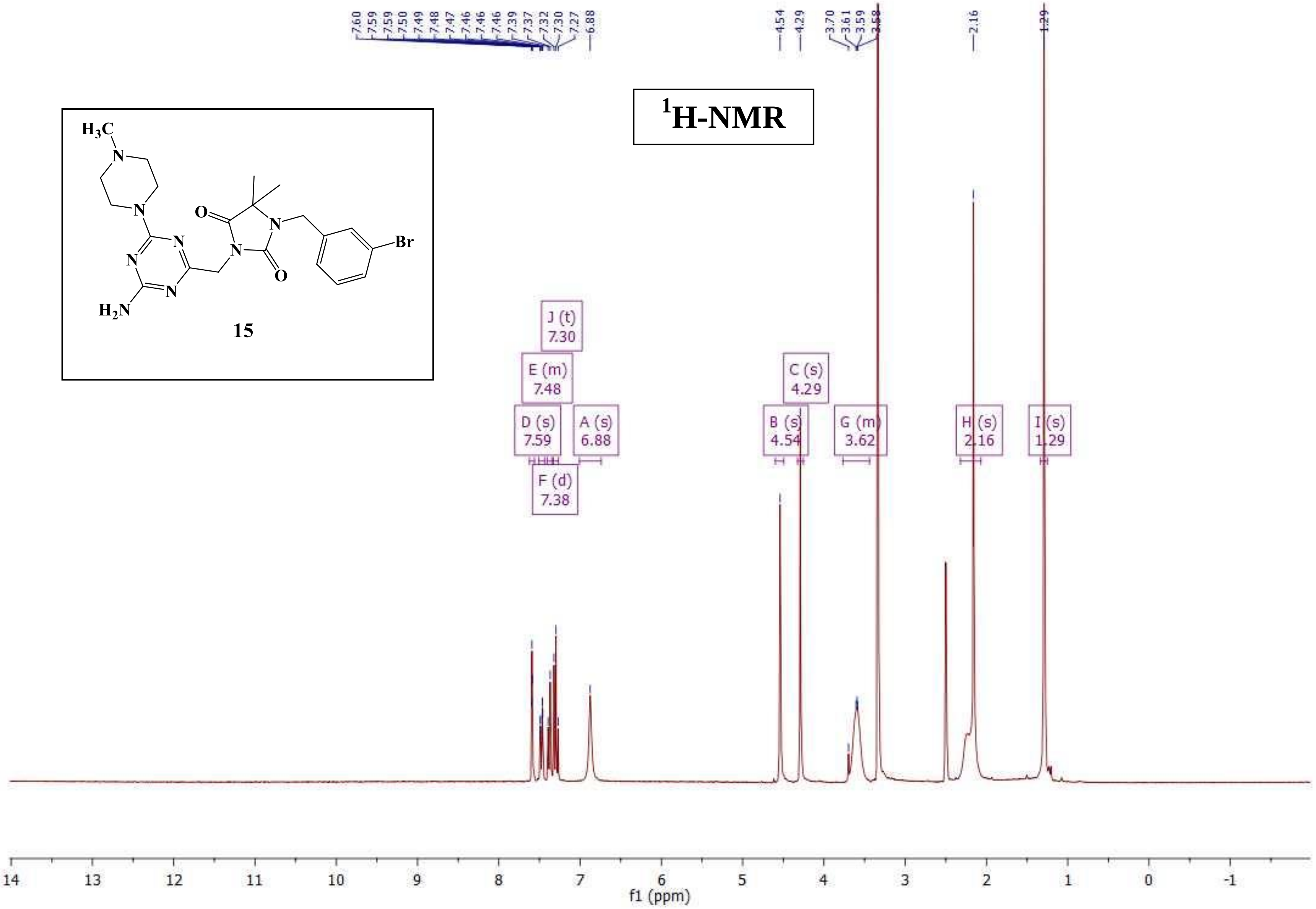

1H-NMR
7.60 7.59 7.59 7.50 7.49 7.48 7.47 7.46 7.46 7.46 7.39 7.37 7.32 7.30 7.27 6.88
4.54 4.29 3.70 3.61 3.59 3.58 2.16 1.29
J (t) 7.30
E (m) 7.48
D (s) 7.59
A (s) 6.88
F (d) 7.38
C (s) 4.29
B (s) 4.54
G (m) 3.62
H (s) 2.16
I (s) 1.29
H3C
N
O
Br
H2N
15
14 13 12 11 10 9 8 7 6 5 4 3 2 1 0 -1
f1 (ppm)

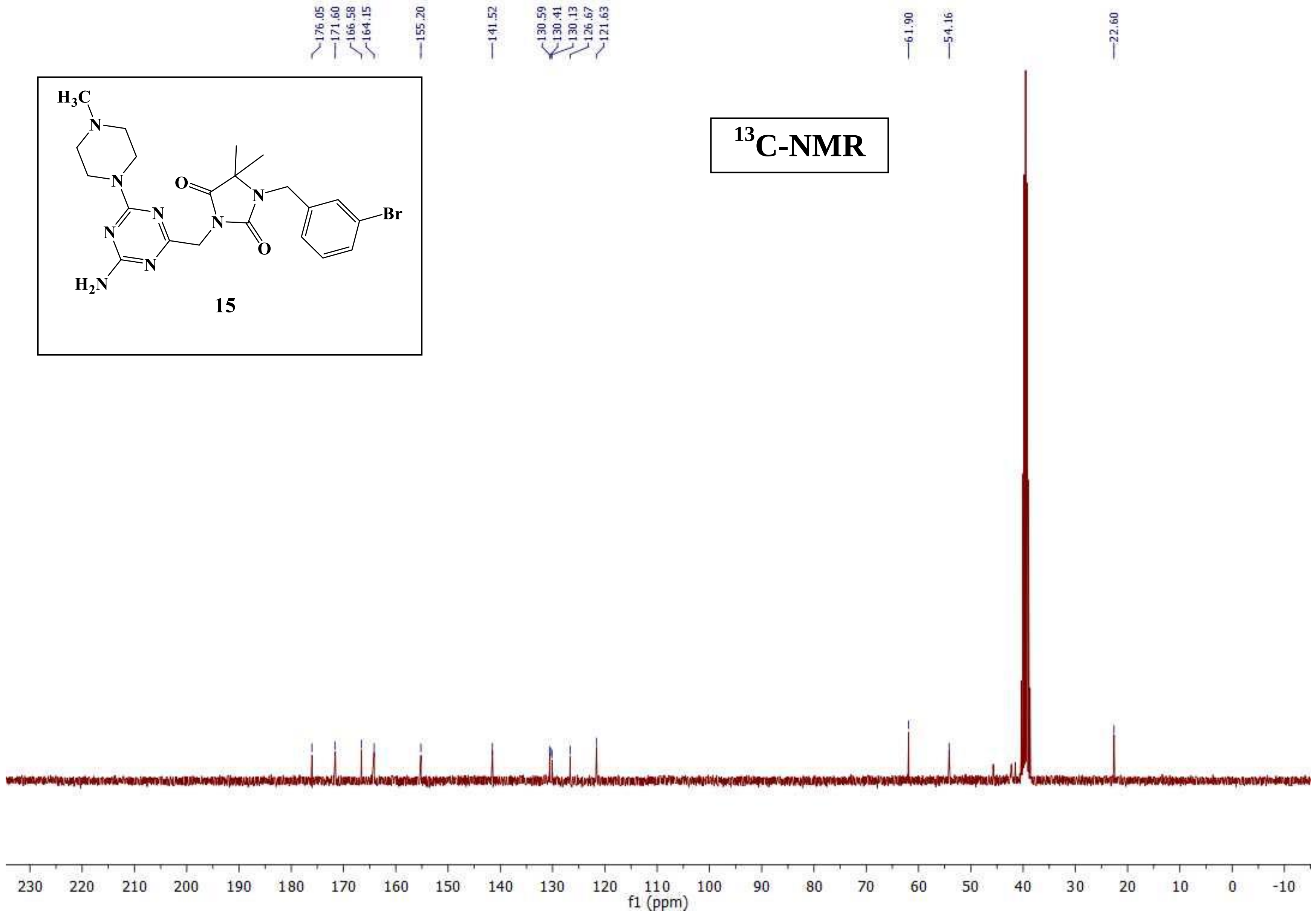
176.05
171.60
166.58
164.15
155.20
141.52
130.59
130.41
130.13
126.67
121.63
61.90
54.16
22.60
^{13}C-NMR
H_3C
N
N
N
N
N
H_2N
O
N
N
O
Br
15
230 220 210 200 190 180 170 160 150 140 130 120 110 100 90 80 70 60 50 40 30 20 10 0 -10
f1 (ppm)

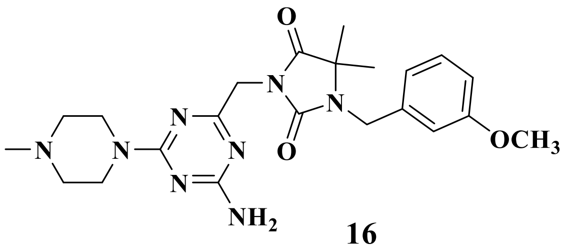
O
N
N
N
N
N
N
N
O
OCH3
NH2

16

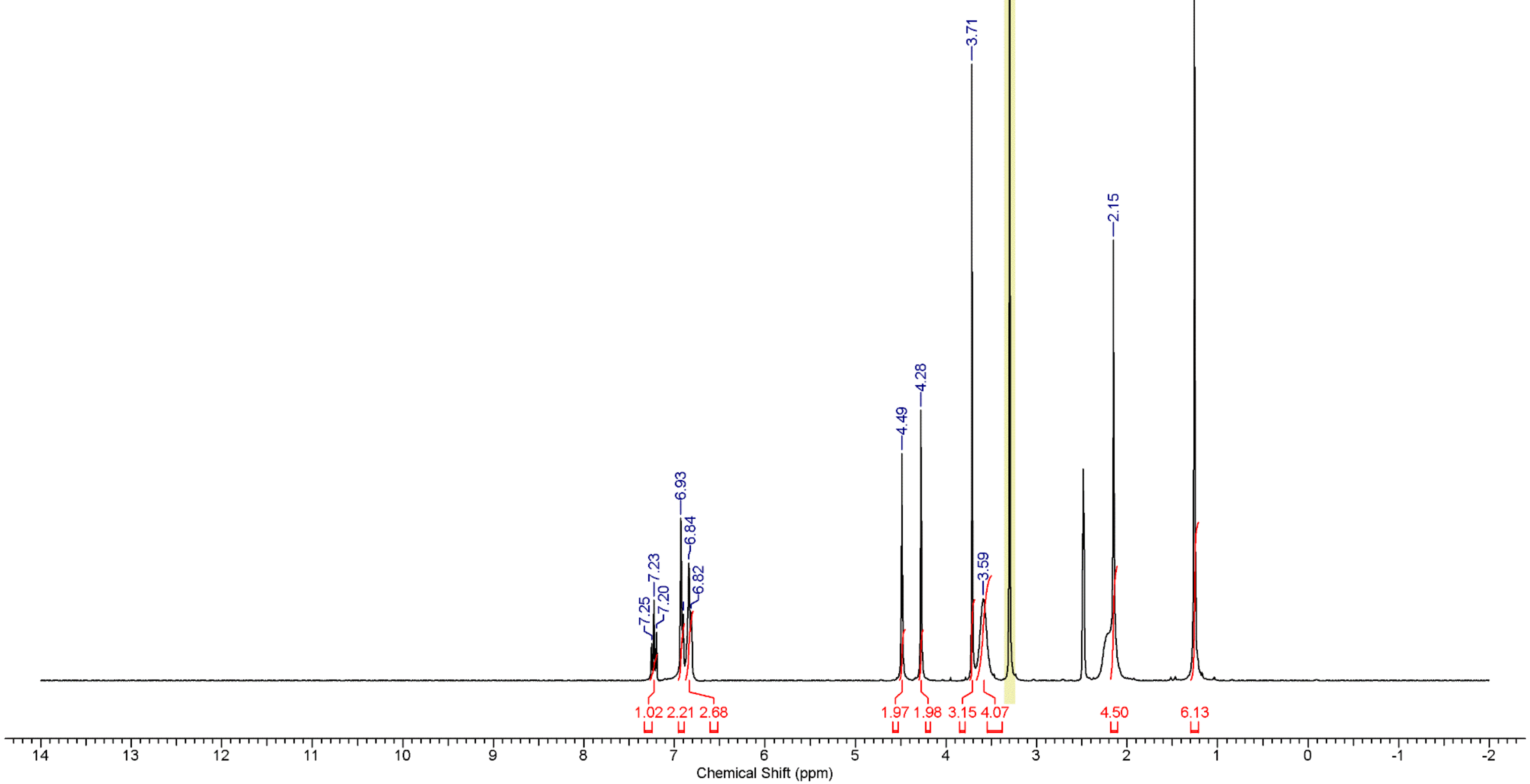
1H-NMR
Water
7.25
7.23
7.20
6.93
6.84
6.82
4.49
4.28
3.71
3.59
2.15
1.25
1.02
2.21
2.68
1.97
1.98
3.15
4.07
4.50
6.13
14
13
12
11
10
9
8
7
6
5
4
3
2
1
0
-1
-2
Chemical Shift (ppm)

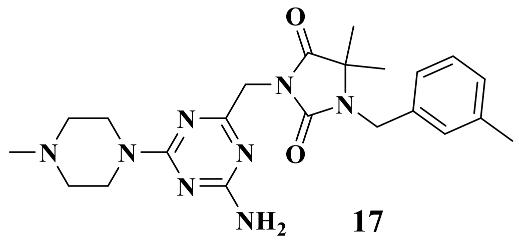

17

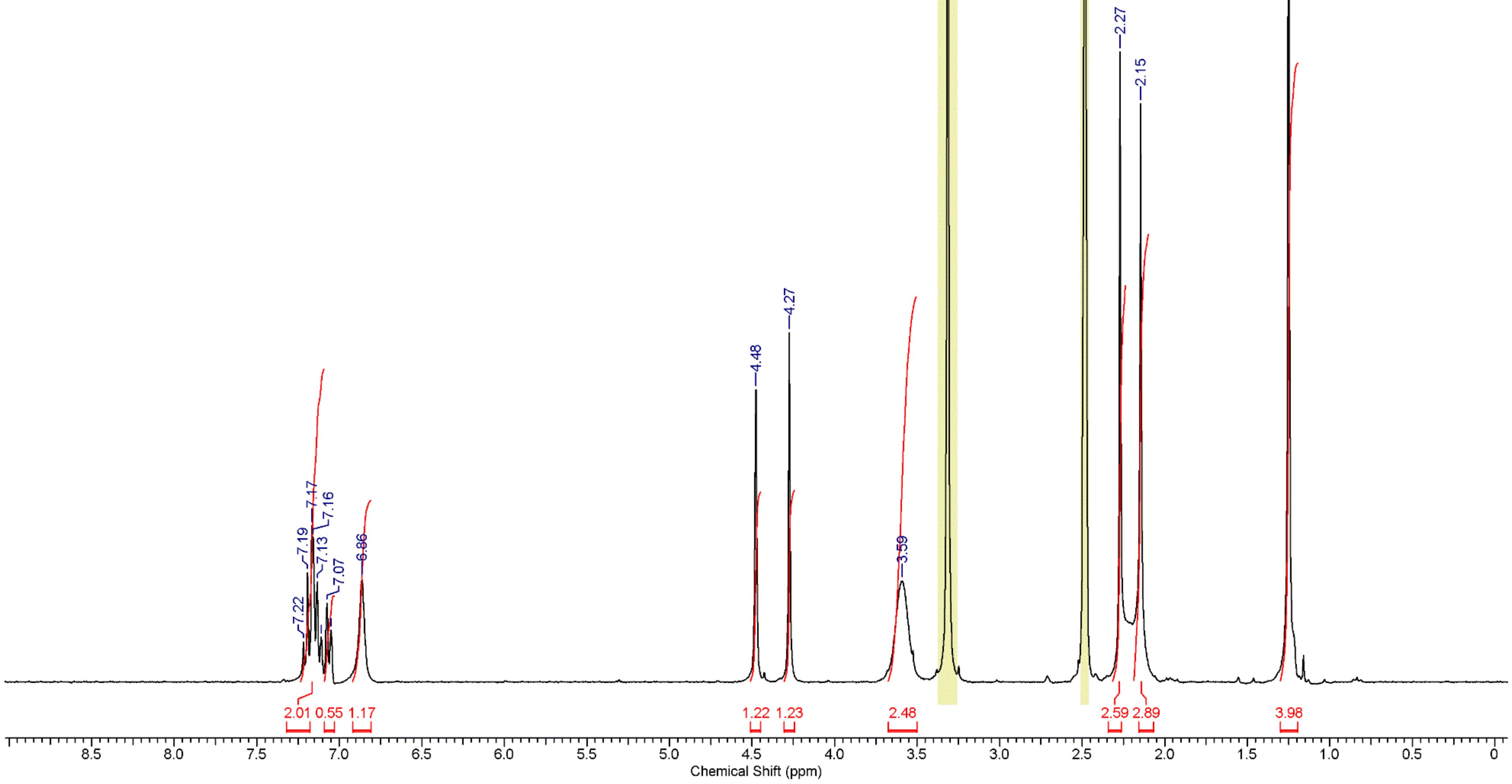

¹H-NMR
Water
DMSO
7.22
7.19
7.17
7.16
7.13
7.07
6.86
4.48
4.27
3.59
2.27
2.15
1.25
2.01
0.55
1.17
1.22
1.23
2.48
2.59
2.89
3.98
8.5
8.0
7.5
7.0
6.5
6.0
5.5
5.0
4.5
4.0
3.5
3.0
2.5
2.0
1.5
1.0
0.5
0
Chemical Shift (ppm)

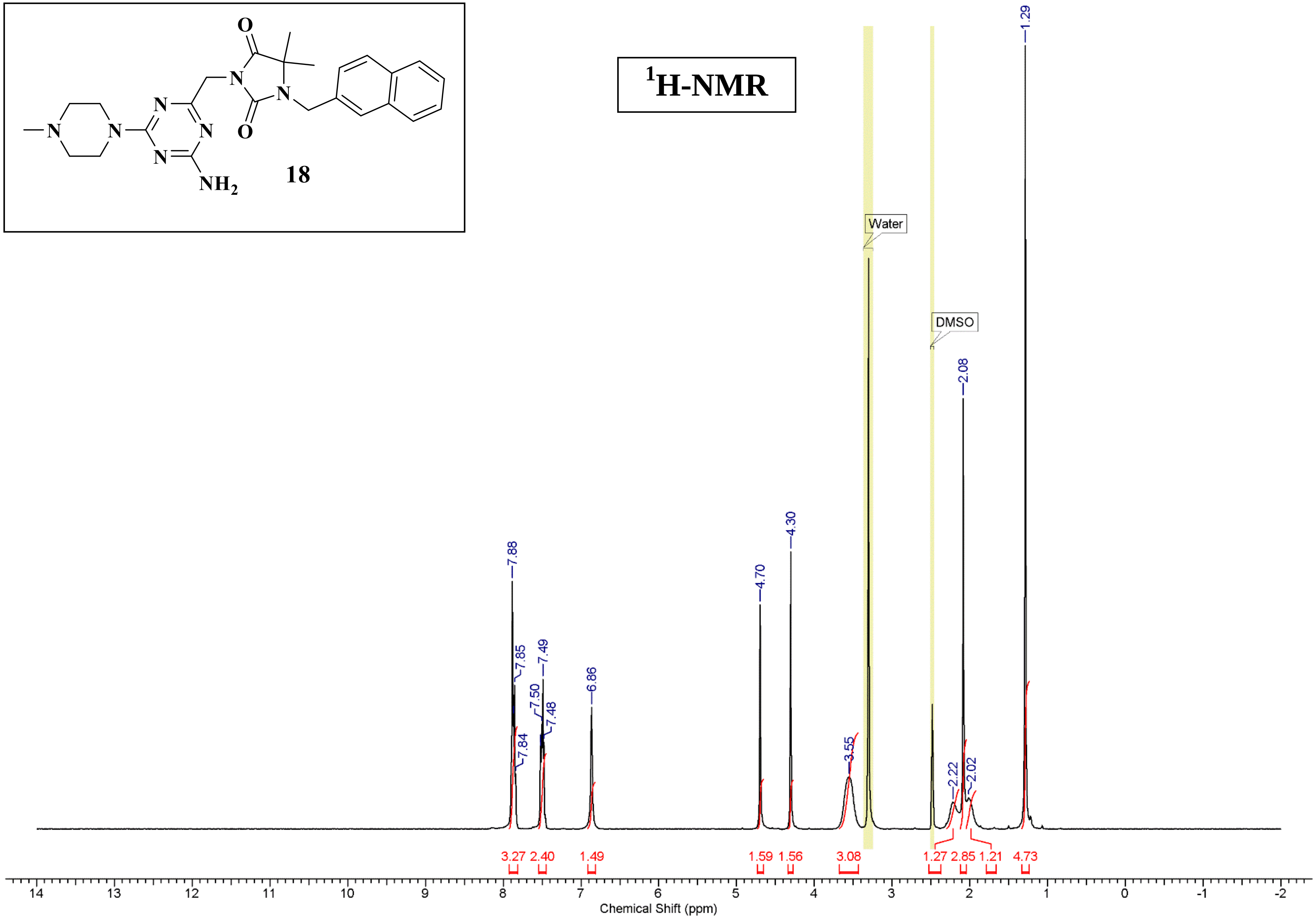
O
N
N
O
N
N
N
N
N
NH2
18
^{1}H-NMR
Water
DMSO
7.88
7.85
7.84
7.50
7.49
7.48
6.86
4.70
4.30
3.55
2.22
2.08
2.02
1.29
3.27 2.40 1.49 1.59 1.56 3.08 1.27 2.85 1.21 4.73
14 13 12 11 10 9 8 7 6 5 4 3 2 1 0 -1 -2
Chemical Shift (ppm)

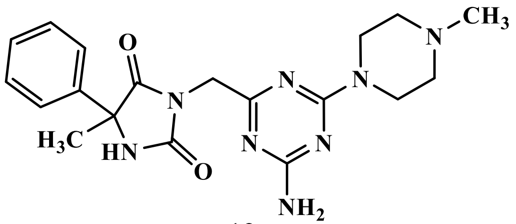
O
N
N
N
N
N
N
CH3
H3C
HN
O
NH2

19

^{1}H-NMR

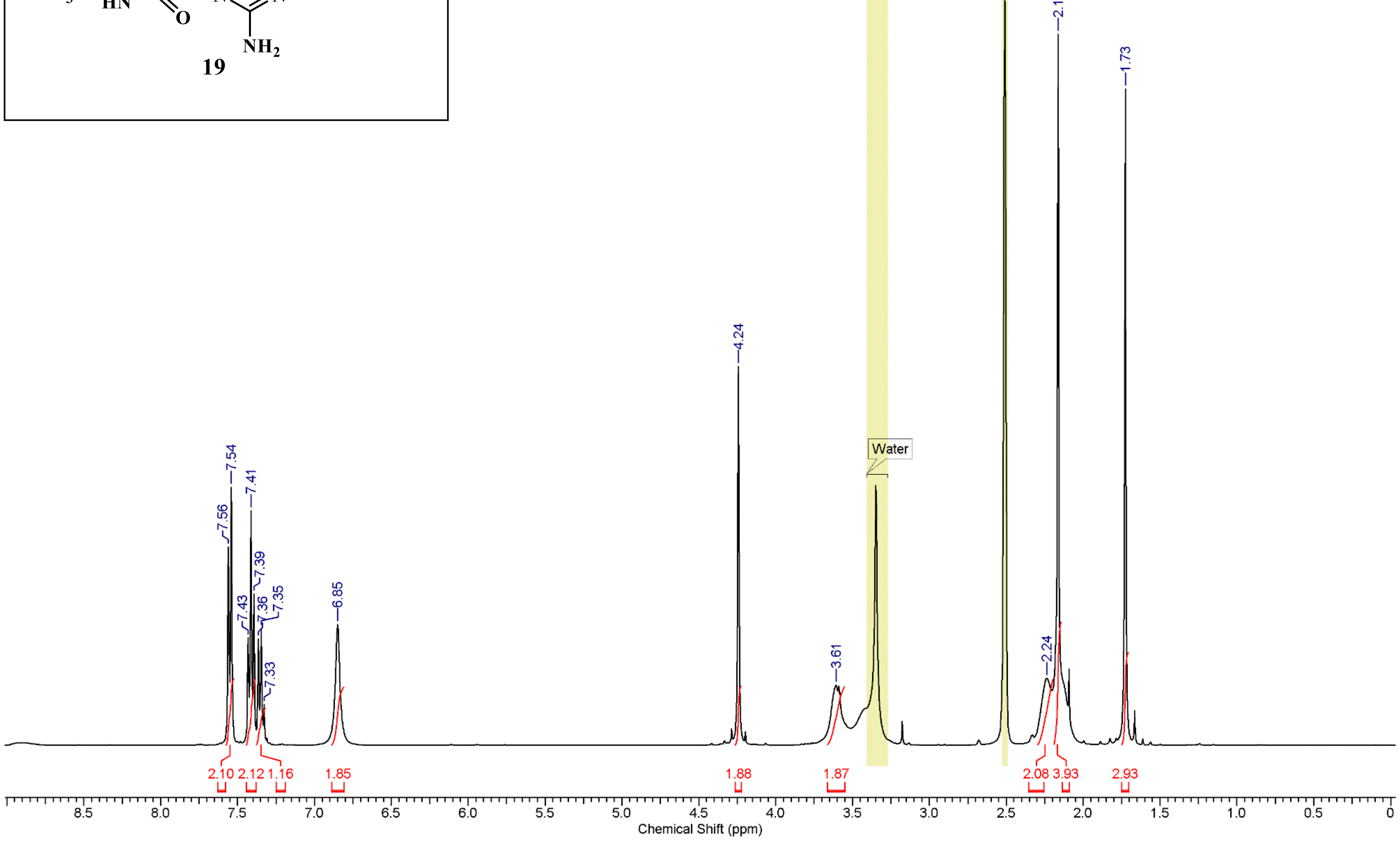
DMSO
Water
7.56
7.54
7.43
7.41
7.39
7.36
7.35
7.33
6.85
4.24
3.61
2.24
2.16
1.73
2.10
2.12
1.16
1.85
1.88
1.87
2.08
3.93
2.93
8.5
8.0
7.5
7.0
6.5
6.0
5.5
5.0
4.5
4.0
3.5
3.0
2.5
2.0
1.5
1.0
0.5
0
Chemical Shift (ppm)

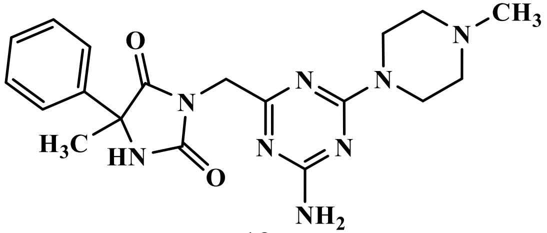
O
N
N
N
N
N
N
CH3
H3C
HN
O
NH2

19

^{13}C-NMR

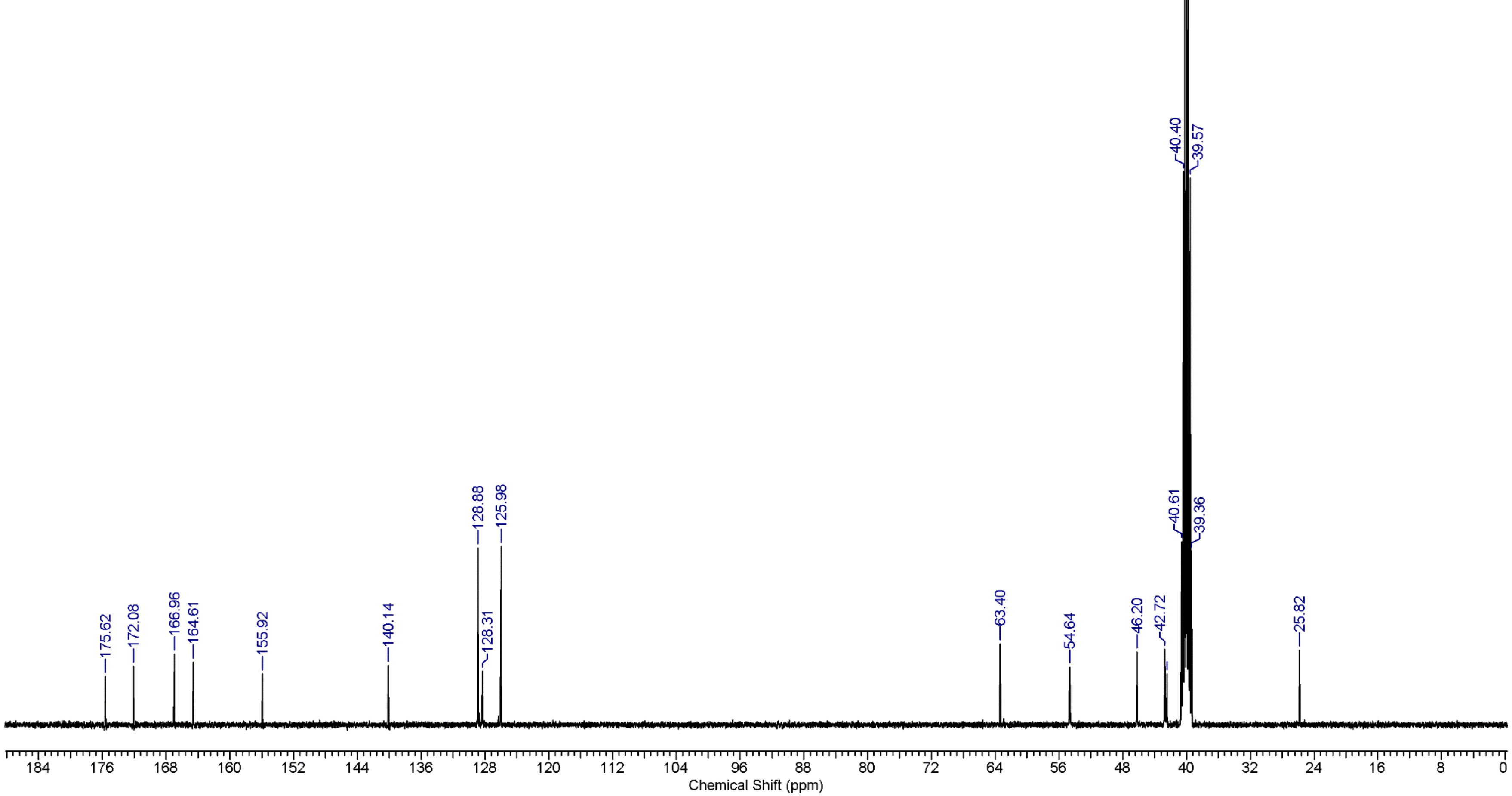
175.62
172.08
166.96
164.61
155.92
140.14
128.88
128.31
125.98
63.40
54.64
46.20
42.72
40.61
40.40
40.19
39.98
39.78
39.57
39.36
25.82
184 176 168 160 152 144 136 128 120 112 104 96 88 80 72 64 56 48 40 32 24 16 8 0
Chemical Shift (ppm)

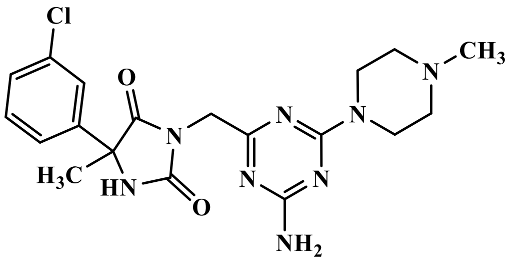
Cl
O
N
CH3
N
N
N
H3C
HN
N
N
O
NH2

20

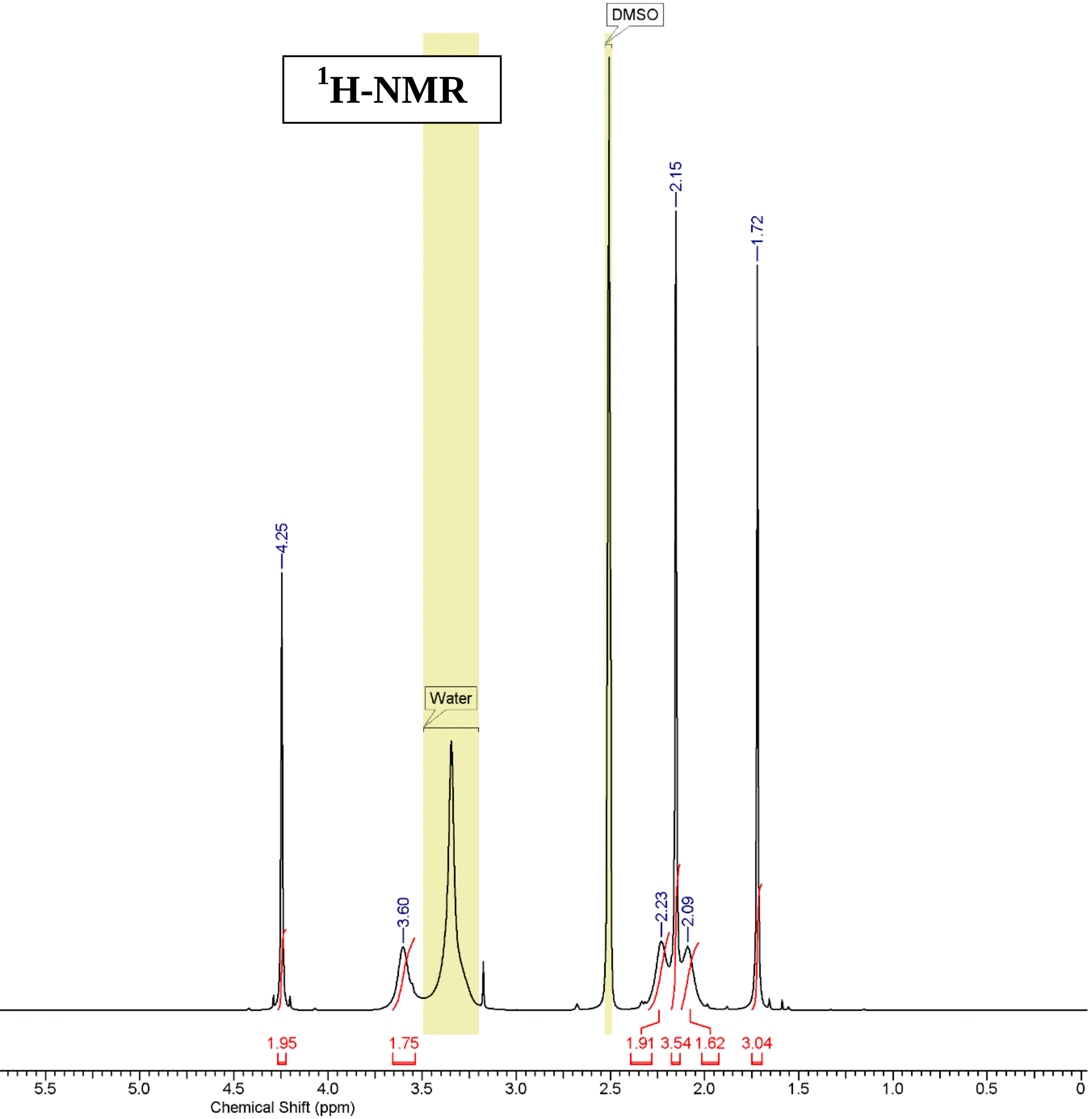
1H-NMR
DMSO
Water
7.58
7.56
7.47
7.45
7.45
6.85
4.25
3.60
2.23
2.15
2.09
1.72
2.08
2.09
1.94
1.95
1.75
1.91
3.54
1.62
3.04
8.0
7.5
7.0
6.5
6.0
5.5
5.0
4.5
4.0
3.5
3.0
2.5
2.0
1.5
1.0
0.5
0
Chemical Shift (ppm)

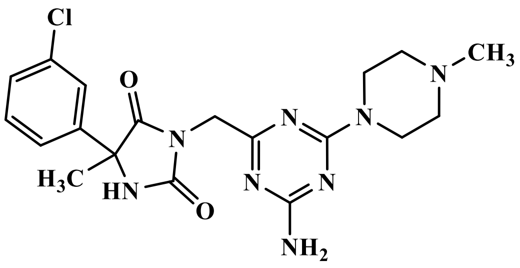
Cl
O
N
N
N
N
N
CH3
H3C
HN
O
N
N
NH2

20

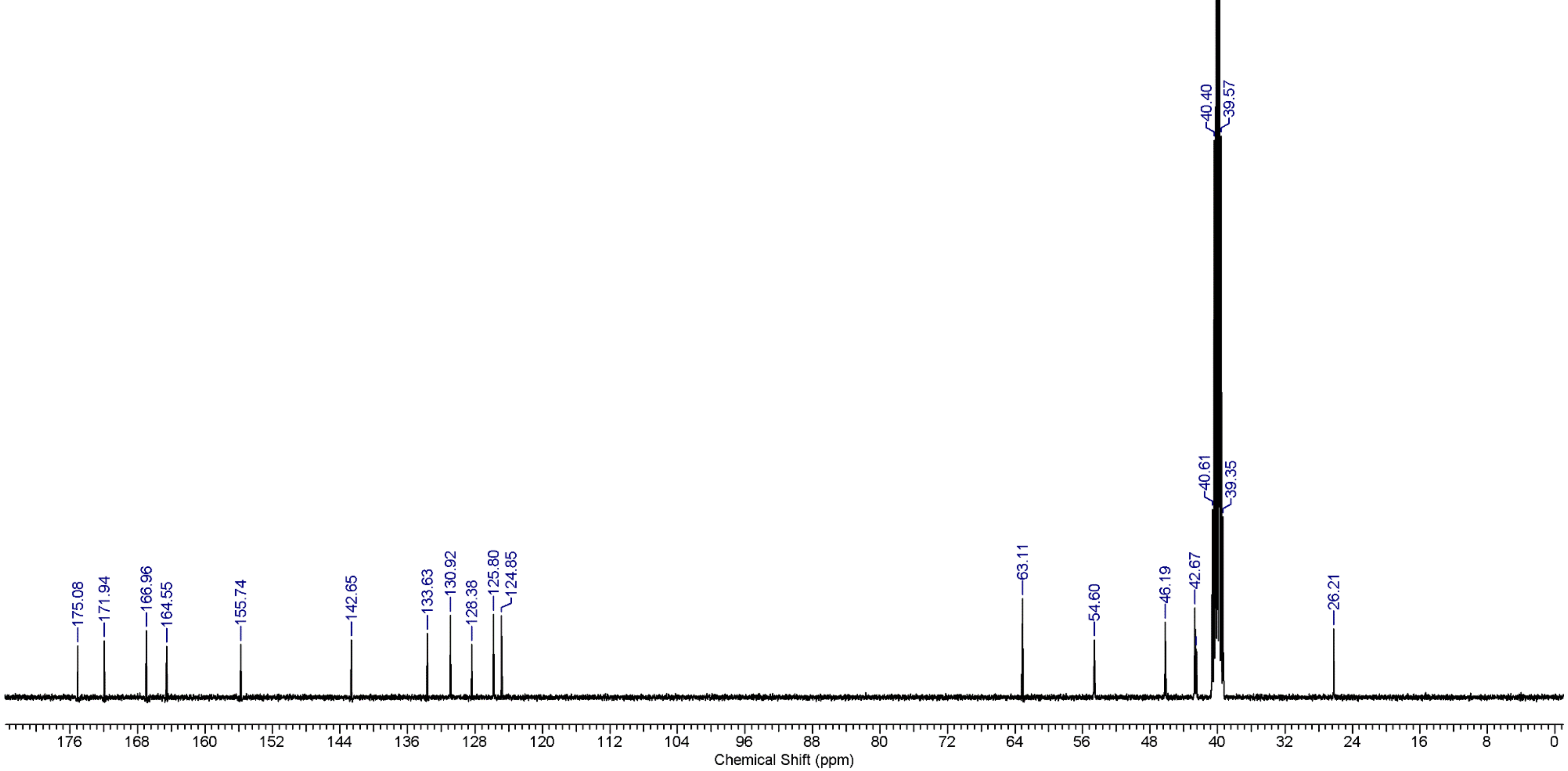
13C-NMR
40.19
39.98
39.77
40.40
39.57
40.61
39.35
175.08
171.94
166.96
164.55
155.74
142.65
133.63
130.92
128.38
125.80
124.85
63.11
54.60
46.19
42.67
26.21
176
168
160
152
144
136
128
120
112
104
96
88
80
72
64
56
48
40
32
24
16
8
0
Chemical Shift (ppm)

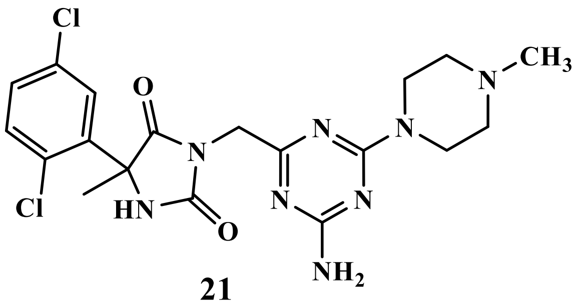
Cl
Cl
O
O
N
HN
N
N
N
N
N
CH3
NH2

21

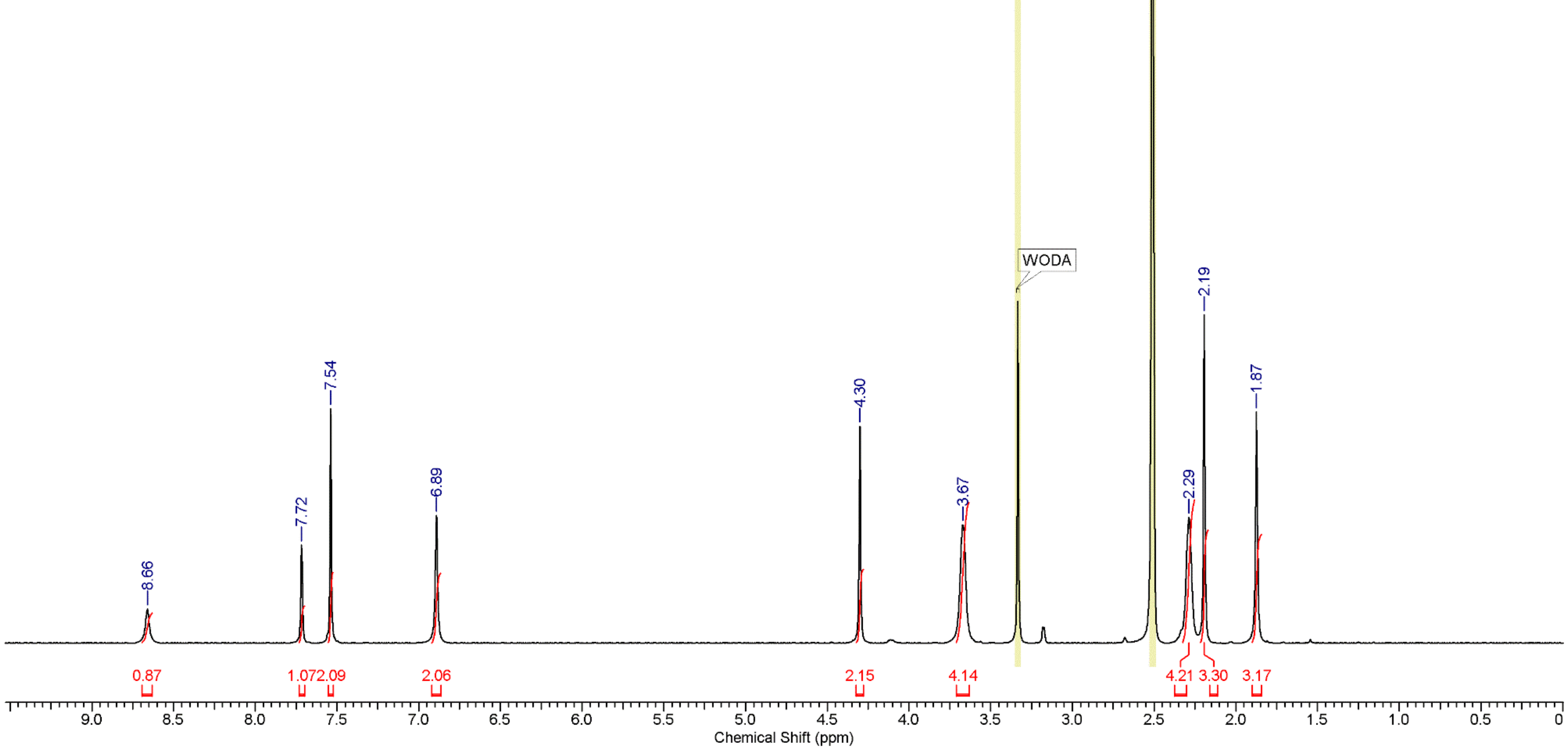
1H-NMR
DMSO
WODA
8.66
7.72
7.54
6.89
4.30
3.67
2.29
2.19
1.87
0.87
1.07
2.09
2.06
2.15
4.14
4.21
3.30
3.17
9.0
8.5
8.0
7.5
7.0
6.5
6.0
5.5
5.0
4.5
4.0
3.5
3.0
2.5
2.0
1.5
1.0
0.5
0
Chemical Shift (ppm)

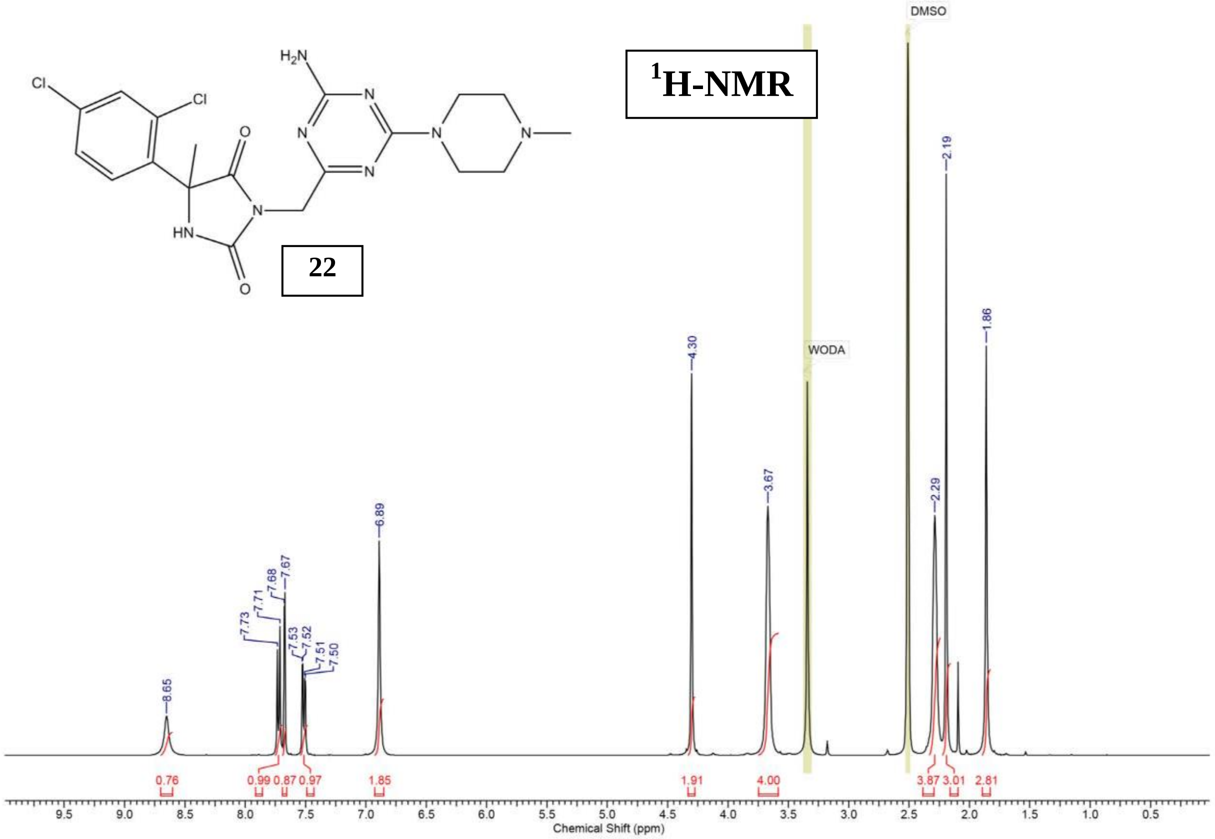

Cl
Cl
O
H2N
N
N
N
N
N
N
N
HN
O
22
1H-NMR
DMSO
WODA
8.65
7.73
7.71
7.68
7.67
7.53
7.52
7.51
7.50
6.89
4.30
3.67
2.29
2.19
1.86
0.76
0.99
0.87
0.97
1.85
1.91
4.00
3.87
3.01
2.81
Chemical Shift (ppm)

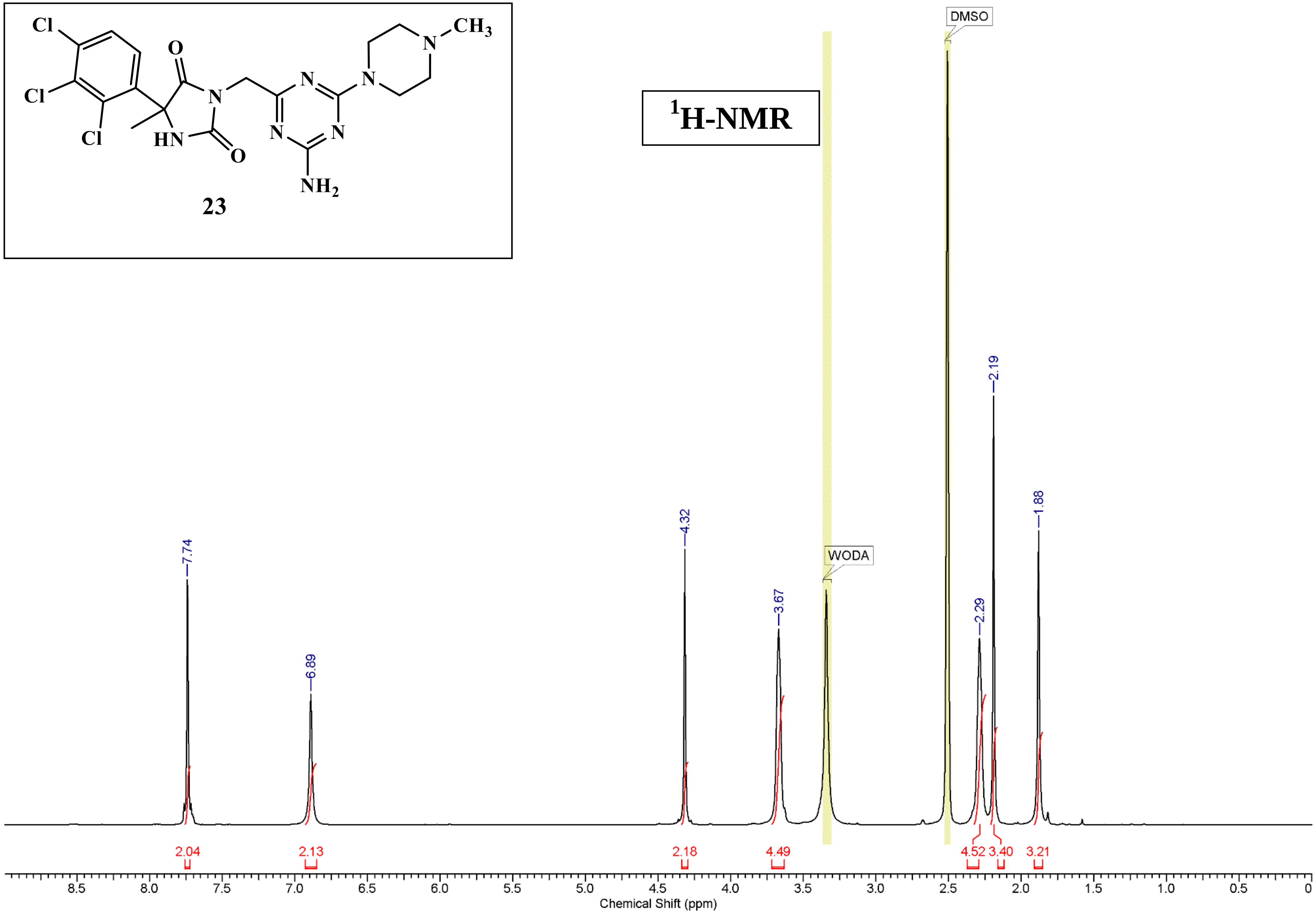

23
¹H-NMR
DMSO
WODA
7.74
6.89
4.32
3.67
2.29
2.19
1.88
2.04
2.13
2.18
4.49
4.52
3.40
3.21
8.5 8.0 7.5 7.0 6.5 6.0 5.5 5.0 4.5 4.0 3.5 3.0 2.5 2.0 1.5 1.0 0.5 0
Chemical Shift (ppm)

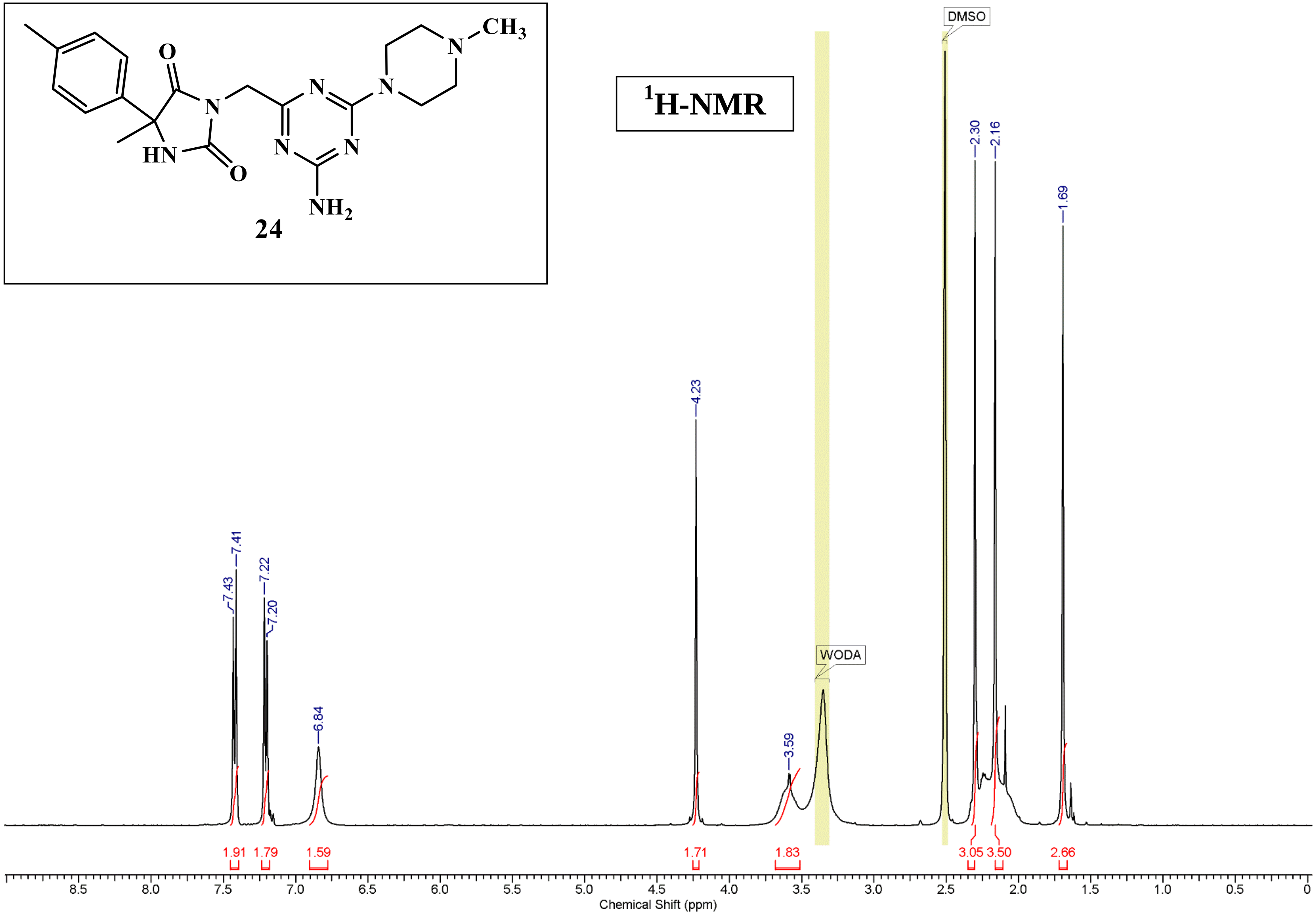

O
N
CH3
N
N
N
HN
O
N
N
NH2
24
1H-NMR
DMSO
WODA
7.43
7.41
7.22
7.20
6.84
4.23
3.59
2.30
2.16
1.69
1.91
1.79
1.59
1.71
1.83
3.05
3.50
2.66
8.5
8.0
7.5
7.0
6.5
6.0
5.5
5.0
4.5
4.0
3.5
3.0
2.5
2.0
1.5
1.0
0.5
0
Chemical Shift (ppm)

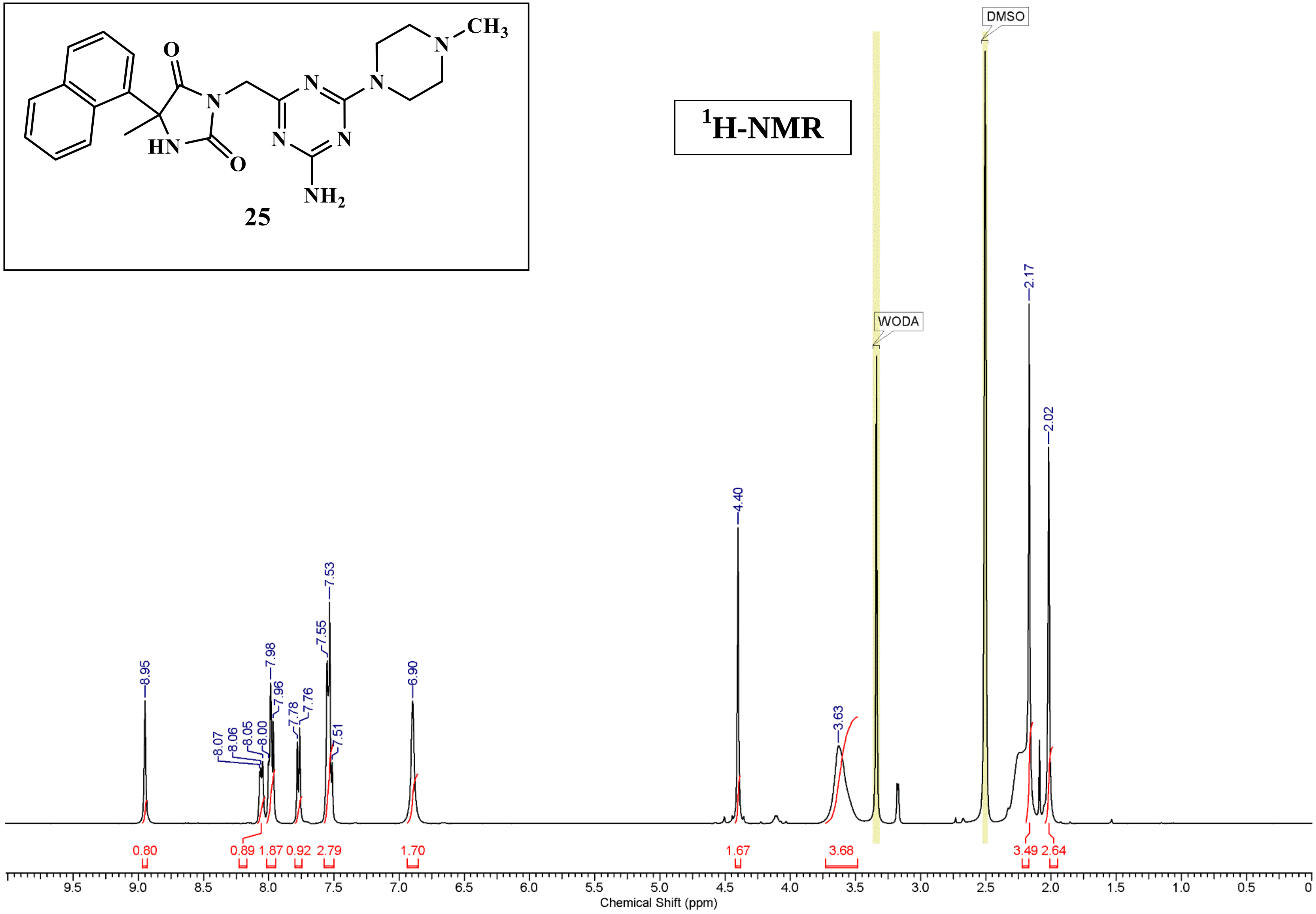

25
1H-NMR
WODA
DMSO
8.95
8.07
8.06
8.05
8.00
7.98
7.96
7.78
7.76
7.55
7.53
7.51
6.90
4.40
3.63
2.17
2.02
0.80
0.89
1.87
0.92
2.79
1.70
1.67
3.68
3.49
2.64
Chemical Shift (ppm)

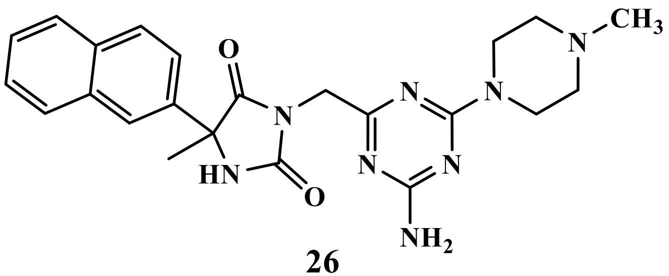

26

^{1}H-NMR

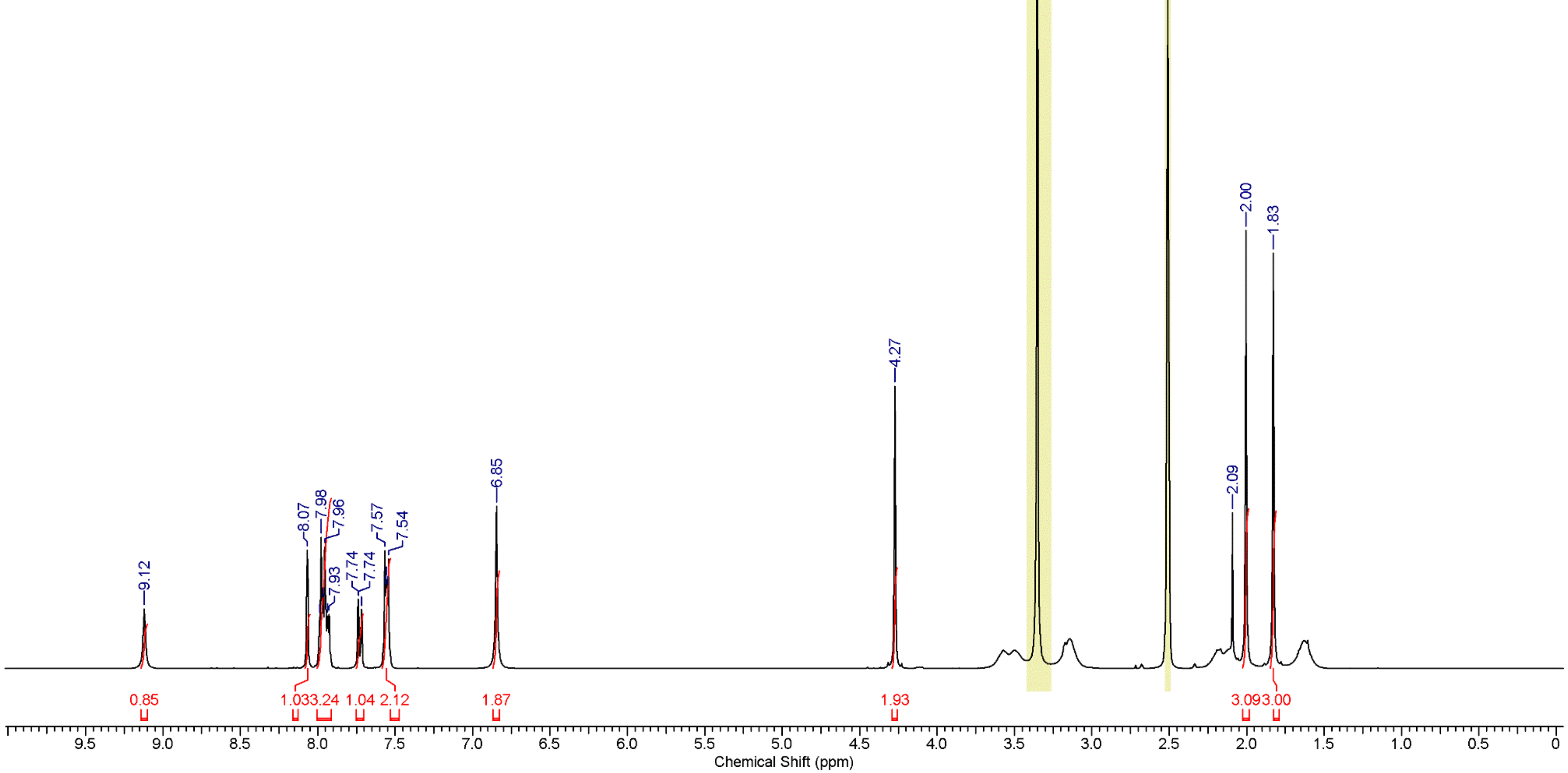
Water
DMSO
9.12
8.07
7.98
7.96
7.93
7.74
7.74
7.57
7.54
6.85
4.27
2.09
2.00
1.83
0.85
1.03
3.24
1.04
2.12
1.87
1.93
3.09
3.00
Chemical Shift (ppm)

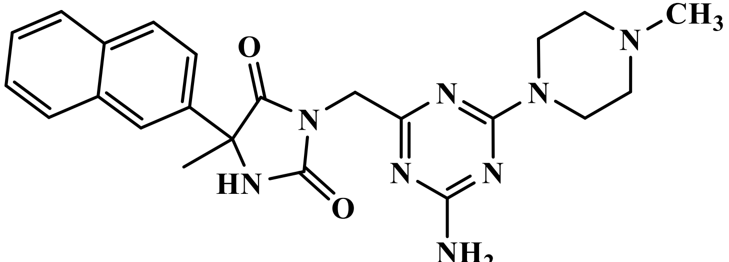
O
N
HN
O
N
N
N
N
N
CH3
NH2

26

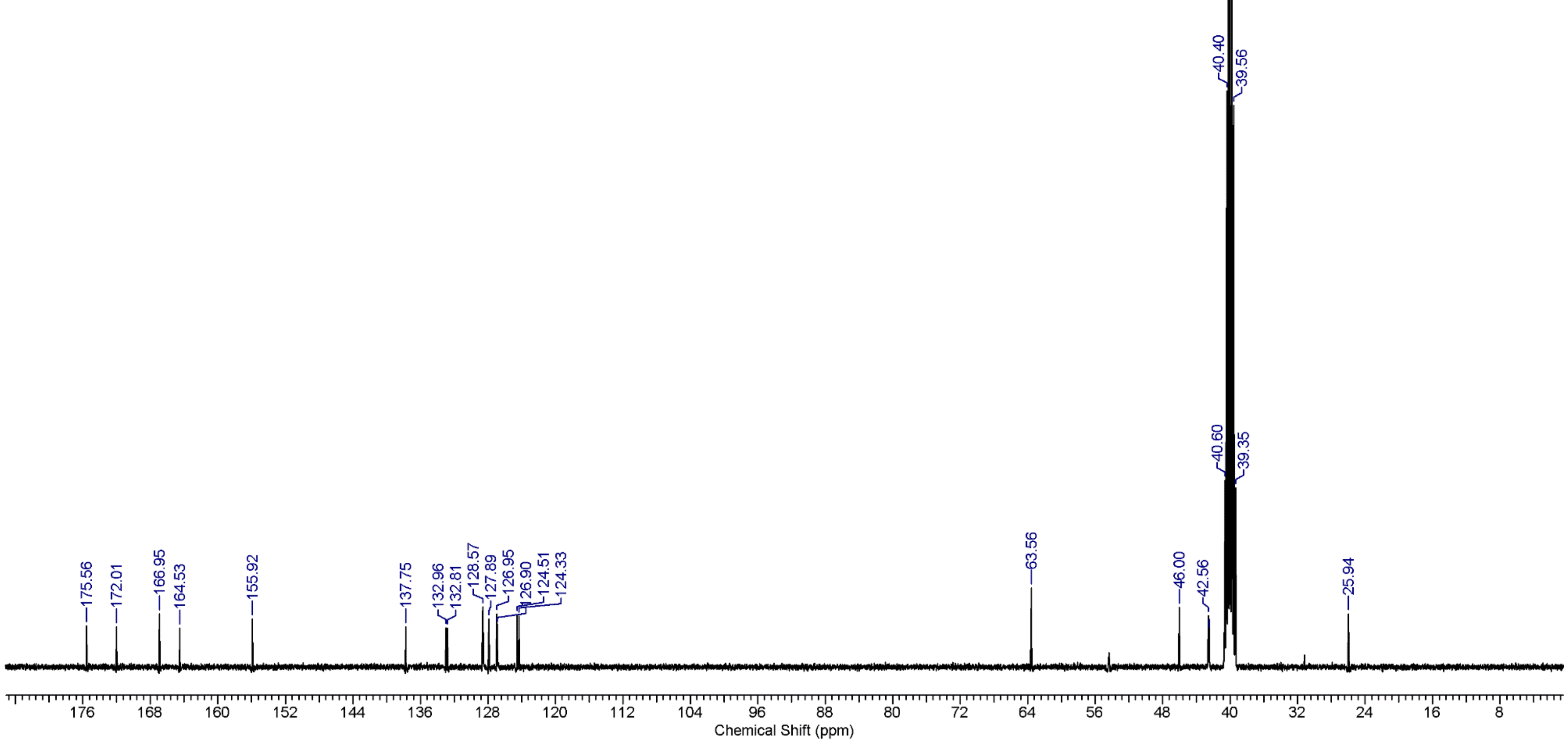
13C-NMR
175.56
172.01
166.95
164.53
155.92
137.75
132.96
132.81
128.57
127.89
126.95
126.90
124.51
124.33
63.56
46.00
42.56
40.60
40.40
40.19
39.98
39.77
39.56
39.35
25.94
176
168
160
152
144
136
128
120
112
104
96
88
80
72
64
56
48
40
32
24
16
8
Chemical Shift (ppm)
